# IMplementation of the Preterm Birth Surveillance PAthway: a RealisT evaluation (The IMPART Study)

**DOI:** 10.1186/s43058-024-00594-9

**Published:** 2024-05-21

**Authors:** Naomi Carlisle, Sonia Dalkin, Andrew H Shennan, Jane Sandall

**Affiliations:** 1 Department of Women and Children’s Health, The School of Life Course & Population Sciences, King’s College London, 10th Floor North Wing, St Thomas’ Hospital, Westminster Bridge Road, London, SE1 7EH UK; 2 Faculty of Health and Life Sciences, Northumbria University, Newcastle upon Tyne, UK

**Keywords:** Preterm, Pathway, Implementation, Realist, Evaluation

## Abstract

**Background:**

In the UK, 7.6% of babies are born preterm, which the Department of Health aims to decrease to 6% by 2025. To advance this, NHS England released Saving Babies Lives Care Bundle Version 2 Element 5, recommending the Preterm Birth Pathway for women at risk of preterm birth. The success of this new pathway depends on its implementation. The IMPART (IMplementation of the Preterm Birth Surveillance PAthway: a RealisT evaluation) study aimed to research how, why, for whom, to what extent and in what contexts the prediction and prevention aspects of Preterm Birth Surveillance Pathway is implemented through a realist evaluation. Realist implementation studies are growing in popularity.

**Methods:**

Initial programme theories were developed through a realist informed literature scope, interviews with developers of the NHS England guidance, and a national questionnaire of current practice. Implementation theory was utilised in developing the programme theories. Data (interviews and observations with staff and women) were undertaken in 3 case sites in England to ‘test’ the programme theories. Substantive theory was utilised during data analysis to interpret and refine the theories on how implementation could be improved.

**Results:**

Three explanatory areas were developed: risk assessing and referral; the preterm birth surveillance clinic; and women centred care. Explanatory area 1 dealt with the problems in correct risk assessment and referral to a preterm clinic. Explanatory area 2 focused on how once a correct referral has been made to a preterm clinic, knowledgeable and supported clinicians can deliver a well-functioning clinic. Explanatory area 3 concentrated on how the pathway delivers appropriate care to women.

**Conclusions:**

The IMPART study provides several areas where implementation could be improved. These include educating clinicians on knowledge of risk factors and the purpose of the preterm clinic, having a multidisciplinary preterm team (including a preterm midwife) with specialist preterm knowledge and skills (including transvaginal cervical scanning skills), and sites actively working with their local network. This multidisciplinary preterm team are placed to deliver continuity of care for women at high-risk of preterm birth, being attentive to their history but also ensuring they are not defined by their risk status.

**Trial registration:**

ISRCTN57127874.

**Supplementary Information:**

The online version contains supplementary material available at 10.1186/s43058-024-00594-9.

Contributions to the literature

- A realist evaluation was undertaken to understand implementation of the preterm birth surveillance pathway.
- Substantive implementation theory was utilised during data analysis to interpret and refine the theories on how implementation could be improved.
- Lack of knowledge and practical difficulties can lead to incorrect preterm birth risk assessment occurring.
- A core specialist multidisciplinary preterm team can develop concentrated knowledge and expertise.
- If ordinary aspects of a women’s pregnancy are not overshadowed, she will not feel defined by her high-risk status.

## Background

In the UK, 7.6% of babies are born preterm (before 37 weeks’ gestation) [1]. Globally, preterm birth is accountable for around 40% of neonatal deaths [2], with survivors frequently suffering short and long-term sequelae [3, 4].

The Department of Health aims to decrease the preterm birth rate from 8 to 6% by 2025 [5, 6]. To help meet this, NHS England (which leads the National Health Service (NHS) health system in England) released Saving Babies Lives Care Bundle Version 2 [7], which recommended a preterm birth pathway for women at risk of preterm birth [8]. The pathway encompassed three aspects: predicting women at risk of preterm birth, preventing preterm birth, and preparation for imminent preterm births. The pathway was incorporated into the NHS standard contract for 2019/20 [8], meaning all hospitals in England providing NHS services should be providing this pathway. In England, less than 1% of women have exclusive private maternity care [9], meaning the vast majority of women are cared for by NHS staff in an NHS hospital.

The new pathway proposed midwives assessing all women at their booking (first) appointment based on her medical history. Women will be assessed as being at low, intermediate, or high-risk of preterm birth. Women considered as intermediate or high-risk ought to be referred to a Preterm Birth Surveillance Clinic. If referred, women are typically offered screening tests (e.g. Fetal Fibronectin biomarker swab, and transvaginal cervical length ultrasound scans [10, 11]), so appropriate medical interventions can be recommended (e.g., cervical cerclage).

This guidance aims to reduce variations in care for pregnant women at risk of preterm birth and is a substantial change for hospitals. Despite the vast majority of women in England being cared for by NHS staff in an NHS hospital for their pregnancy and birth, there are variations of care within the NHS. Before its publication, only 33 consultant-led hospitals had a Preterm Surveillance Clinic out of 187 hospitals offering obstetric care in the UK [12]. As most hospitals do not have a preterm clinic, implementing this pathway will require substantial re-organisation. The success of this pathway will be ascertained by how it is implemented in hospitals [7]. This study therefore aimed to research how, why, for whom, to what extent and in what contexts the prediction and prevention aspects of the Preterm Birth Surveillance Pathway are implemented through a realist evaluation [13].

## Methods

### Realist evaluation study design

The full methods have been reported elsewhere [13]. Realist implementation studies are growing in popularity to develop genuine knowledge about what works in implementation [14]. Evaluating national programmes aiming to standardise care through a realist evaluation is suited to understanding implementation of complex service interventions, such as the preterm pathway [15–17].

**Fig. 1:**
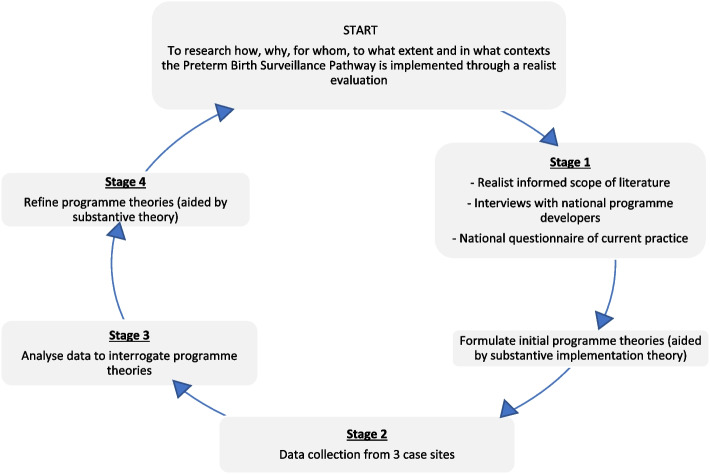
The IMPART study realist evaluation

The first stage of a realist evaluation is to elicit and formulate (initial) programme theories (Fig. 1). A programme (a service, intervention or policy) provides a resource, an opportunity, or a constraint – all of which can affect the decision-making process of its intended target group [15]. The interaction between what a programme provides, and the reasoning of its intended targets, is a mechanism [18, 19]. Mechanisms are encouraged in specific contexts, which leads to intended and unintended outcomes. A programme theory formulates a proposed relationship between a context (C) + mechanism (M) = outcome (O) – known as CMO configuration [18].

### Stages of a realist evaluation

Whilst realist evaluation is an iterative, non-linear process, Pawson and Tilley (1997) [20] outline research stages which this study utilised (Fig. 1):

- Stage 1*: *Formulating initial programme theories about implementation of the Preterm Birth Surveillance Pathway (using CMO hypotheses for how each programme component works) through a realist informed scope of the literature, interviews with national level programme developers and a national questionnaire of current preterm practice (published elsewhere [21]). This can be seen in Supplementary File 1.
- Stage 2: Collecting data (interviews, observations and hospital guidelines) from 3 case sites to ‘test’ the programme theories.
- Stage 3: Analysing data using a realist logic of analysis to interrogate programme theories.
- Stage 4: Synthesising and interpreting to refine initial theories, leading to theories of how implementation of the Preterm Birth Surveillance Pathway can be improved.

### Case sites and participants

 Data was collected from 3 case sites in Stage 2. The three case sites differed in size, clinical preterm expertise, and demographics of local service users (Table 1). The 3 case sites were based in different Local Maternity and Neonatal System (LMNS) areas across England, which was important because the Preterm Birth Surveillance Pathway specifies that women with very complex histories require referral to tertiary clinics within their LMNS area.

**Table 1:** Case sites and data collected

				TOTAL	MEAN
**Sites**	Site L	Site H	Site Y	3	-
**Staff interviews undertaken**	4	5	4	13	-
**Staff interviewed job title**	2 x Preterm midwives, 1 x Obstetric Registrar (preterm specialist), 1 x Consultant Obstetrician (preterm lead)	1 x Maternity Support Worker, 1 x Consultant Obstetrician, 1 x Consultant Obstetrician (preterm lead), 1 x Community midwife, 1 x Antenatal clinic midwife	1 x Maternity Matron, 1 x Superintendent sonographer, 1 x Lead midwife antenatal clinic, 1 x Obstetric Senior House Officer		
**Women interviews undertaken**	4	5	2	11	-
Mean IMD decile based on postcode of women interviewed. Ranked 1–10 (most-least deprived)	7	3	5	-	5
**Appointments observed**	56 (comprising 56 women and 6 staff members)	25 (comprising 25 women and 4 staff members)	6 (comprising of 6 women and 5 staff members)	87	-
Mean IMD decile based on postcode of women observed.	5	3	6	-	5
**Hours spent observing**	28	16	12	56	-
**Number of births per year**	10,000 across the trust	6,000	1,400	-	
**Preterm birth surveillance clinic?**	Yes. Well established tertiary clinic. Two consultant obstetrician leads and two preterm midwives.	Yes. New clinic with one consultant obstetrician lead. No preterm midwives (but are in process of recruiting).	No. ‘Preterm clinic’ runs within the antenatal clinic, with a named consultant only.		
**Frequency of preterm surveillance clinic**	Two clinics a week across the trust.	Once a fortnight.	Ad-hoc, women are seen as required in antenatal clinic.		
**Preterm surveillance screening tests available**	Fetal Fibronectin and cervical length scanning well established.	Fetal Fibronectin (recently commenced) and cervical length scanning.	Cervical length scanning undertaken separately by the ultrasound department.		

A purposive realist sample of participants was selected, using participants demographic, medical and obstetric history details to ensure all of the relevant programme theories were ‘tested’ [22] (Table 1). Staff at the 3 case sites were interviewed between July to November 2022. Women at the 3 case sites were interviewed between July 2022 to February 2023. Ethnographic observations at the 3 case sites took place between July 2022 to November 2022.

### Substantive theory to aid development of the initial programme theories

Realist evaluation is an iterative process, drawing on substantive theory throughout to enhance explanatory potential [19]. As the IMPART study was evaluating implementation of the preterm pathway, a suitable implementation theory, framework or model was sought when developing the initial programme theories [23–25] to ensure they were effective and inclusive of the knowledge already in the field [26]. Normalization Process Theory was determined as the most appropriate due to its focus on implementing a complex intervention [27, 28] which the preterm pathway is (score of 2, ‘good fit’ [29]).

### Data analysis

The initial programme theories (Supplementary File 1) were inputted as NVivo nodes [30, 31]. The raw data from the three case sites (qualitative transcripts from interviews with staff and women, and field notes from observations) was iteratively analysed using a realist CMO lens to make sense of, test and refine the programme theory nodes.

## Results

**Fig. 2:**
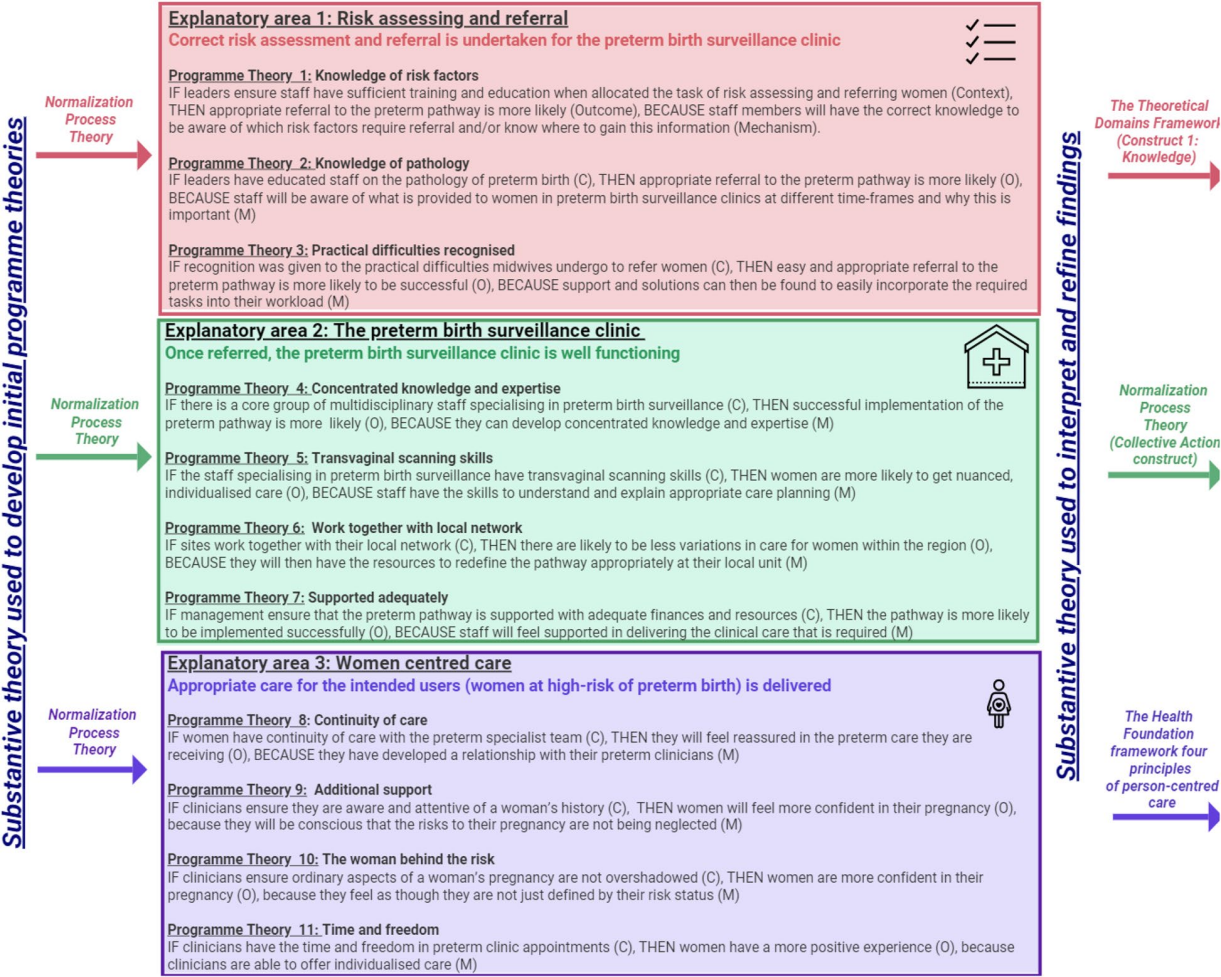
The three explanatory areas developed after data analysis, and the programme theories they encompass. Substantive theory was utilised in developing the initial programme theories, and in interpreting and explaining the findings

Three final explanatory areas were developed: risk assessing and referral; the preterm birth surveillance clinic; and women centred care (Fig. 2). Full results and data analysis can be seen in Supplementary File 2.

### Substantive theory to aid interpretation and refinement of the findings

Substantive theory is used in realist evaluation to provide deeper insight into findings and consider transferability [26]. Normalization Process Theory was utilised in developing the initial programme theories, and moderately explained the data (Supplementary File 1). Normalization Process Theory particularly clarified explanatory area 2, ‘the preterm surveillance clinic’. Two additional substantive theories were therefore sought to aid explanation of all the data. The Theoretical Domains Framework aided explanation of explanatory area 1, ‘risk assessing and referral’. Meanwhile The Health Foundation framework, which comprises of four principles of person-centred care, clarified explanatory area 3, ‘women centred care’.

### Explanatory area 1: risk assessing and referral

The first step of the pathway hinges on correct risk assessment, and if appropriate, a referral to a preterm clinic [7].

Three programme theories were refined to describe what led to appropriate risk assessment and referral (Fig. 2). *Programme Theory 1* identifies why risk assessment and referral could be undertaken incorrectly due to a lack of knowledge of preterm birth risk factors. Across all three sites, a lack of midwifery knowledge on risk factors was noted by midwives themselves, other clinicians, and by women. Even if midwives knew what ‘tick box’ risk factors required referral, they did not know or understand the aetiology behind why they required referral.

*Midwife YSO004 in Observation 077: Being honest…no I wouldn’t know why a fully dilated c-section makes you high risk.*

Although not aware of the aetiology, staff thought this knowledge was important to feel confident during appointments. While the focus was on midwives, this lack of knowledge extended to junior obstetricians too. A lack of education before qualification at undergraduate level, and insufficient mandatory training were noted.

Programme Theory 2 identifies that even if there is knowledge of risk factors, a lack of knowledge on the purpose of the pathway can also result in incorrect referral. Rather than staff simply being aware of what preterm birth risk factors were, they also required an understanding of preterm birth pathology, to understand why particular care was offered at certain timeframes.

Leads at all sites recognised the importance of this education on pathology to junior doctors and midwives. However, they were not expecting all staff to be preterm experts.

Consultant obstetrician LSI004: *“…it comes back to trying to educate them about the pathology of preterm birth and I think that’s all they need to know. I don’t think they desperately need to know every ins and outs of what we do in the clinic and when we treat someone and when we wouldn’t because that is nuance…”*.

All sites had tried to navigate this lack of knowledge by double vetting the referrals made to the preterm clinic. While educating staff on the pathology of preterm birth takes time and effort, it should only need to be undertaken once. The double vetting of referrals takes time and effort that continues indefinitely. While it prevents inappropriate referrals that have been made and are not required, it does not prevent referrals not being made even when they would be appropriate. When staff are aware of double vetting, it also undermines them taking responsibility as they can rely on the safety net of an informed person checking their referral.

Programme Theory 3 highlights that practical difficulties can prevent easy and appropriate referral, even if knowledge of risk factors and the purpose of the pathway is present. Practical difficulties, including computer systems, appointments being undertaken in the community, and time, were all highlighted.

Midwife HSI003: *“… the problem with guidelines and our intranet is that if I’m using a computer that is not a [hospital trust] computer, which it’s not, [laughs] I can’t have that at a click of my finger.”*

Many of the practical issues relate to wider problems within the NHS, such as lack of funding and a lack of pragmatic electronic systems. Maternity risk assessment systems that lack consistency and logic can compromise clinicians’ ability in clearly communicating [21]. While it is infeasible for individual staff members to change this, they tried to make small improvements where they could. The lack of wider, national support over these practical issues was raised as particularly frustrating.

### Using substantive theory to aid explanation of explanatory area 1

Domain 1 of The Theoretical Domains Framework involves three constructs (knowledge; procedural knowledge; and knowledge of task environment) which were mapped to the three programme theories for explanatory area 1 on risk assessing and referral.

The first construct, knowledge, is regarding the importance of clinician’s knowledge of a condition and its scientific rationale when implementing the intervention or pathway. This is highlighted in Programme Theory 1, which recognises that if clinicians have the correct knowledge to be aware of risk factors, then appropriate referral to the preterm pathway is more likely. Programme Theory 2 also highlighted the importance of not just knowing the risk factors, but also knowing the pathology of preterm birth.

The second construct, procedural knowledge, concerns the knowledge of how to undertake a given task. While declarative knowledge explains the methods and procedures, procedural knowledge describes translating this into practical skills [32–34]. Examples in Programme Theory 2 were given where a clinician’s lack of knowledge meant they did not know what an appropriate timeframe was for arranging a preterm birth clinic appointment, impeding their ability to translate this knowledge into the practical skill of undertaking a referral appropriately.

The third construct, knowledge of task environment, regards the working environment which can be unfit for purpose. Programme theory 3 highlighted the practical difficulties can prevent easy and appropriate referral, even if knowledge of risk factors the pathway is present.

### Explanatory area 2: the preterm birth surveillance clinic

Explanatory area 1 dealt with the problems in correct risk assessment and referral to preterm clinics. Explanatory area 2 focuses on once a correct referral has been made, ensuring clinicians can deliver a well-functioning clinic.

Four programme theories described what led to a well-functioning clinic (Fig. 2). Programme Theory 4 identifies how if there is a core group of multidisciplinary preterm specialists, they can develop concentrated knowledge and expertise. This allowed them to hone skills in nuanced decision making, while also becoming experts in practical skills such as transvaginal cervical length scans and cerclages.

Consultant obstetrician LSI004: *“…if someone showed me a scan and it said a cervical at 25[mm], or 24[mm], what does that mean? Well it means different in everyone, doesn’t it? If at 24 we’ve massive funnelling and I can see the cervix is under real strain then I’m likely to think oh she’s 20 weeks, she’s delivered at 24 weeks before, she needs a stitch, but if it’s 24 and strong and she’s had a couple of LLETZ procedures, well actually that’s fine… She might not need anything, that’s normal. I know the national guidance talk about 25 as a cut-off, but it’s 25 to consider treatment, it doesn’t mean you have to do something. But that nuanced care only comes with experience…”*.

In units where there is only one preterm specialist who can undertake necessary requirements of the pathway (such as transvaginal scans or cerclages), complications arise when they are sick or take annual leave.

Consultant obstetrician HSI005: *“…currently it’s a one-woman show and that is always a problem. I pretty much run the clinic …it’s not enough. …So that is also the next step, whether we have the Specialist Midwife who I train to do cervical lengths. Because currently…I have to choose my annual leave around the Preterm [laughs] Birth Clinic… it’s become quite niche”*.

In units where there was a core group of staff, they fully appreciated each other’s strengths and trusted in each other’s work.

Preterm specialist midwife LSI001: *“They [the preterm consultant obstetricians] appreciate our input and ability to support these women… There’s a natural respect… the communication is very good between us…we play to each other’s strengths…definitely.”*

A core group of staff with concentrated knowledge and expertise led to more sustainable implementation of the pathway through numerous ways. Firstly, it reduced variation of care for women within that hospital. Secondly, it was easier for a specialist team to impart their knowledge to junior staff members. Thirdly, it was easier for other staff to know who to contact for preterm advice.

Programme Theory 5 highlights that nuanced and individualised care could be missing if transvaginal cervical length scans were not undertaken by the specialist preterm birth team. Sites could have a named consultant for preterm birth, but transvaginal scans could be undertaken by the sonography department (not by them). If standard operating procedure guidance was followed in a black and white manner because of a sonographer’s report, then nuanced and individualised care cannot be given. Numerous conclusions from undertaking a scan oneself were deemed important, rather than a two-dimensional image and a cervical length figure (where a cervix length of < 25 mm is considered short).

Consultant obstetrician LSI004: *“…a scan report of 24 mm means nothing…I want to see the picture and therefore that’s why I think it’s really important for me to be doing the scan. …the first thing I’ll do is repeat the scan myself…because ultrasound, ultimately, is a dynamic process, isn’t it? It’s still images of rubbish really. You need to see exactly what’s happening.”*

While staff at all sites agreed on the importance of transvaginal cervical length scanning, barriers were identified. These included the time and appropriate mentorship required to learn, it not being a requirement for Royal College of Obstetricians and Gynaecologists (RCOG) trainees, and transient staff. While learning transvaginal scanning skills took time, staff did not consider them difficult.

Programme Theory 6 identifies that if sites work together with their local network, they can provide resources to support clinicians in developing knowledge and expertise (such as scanning), to reduce variations of care. However, the personalities of those at tertiary units with expertise could hinder this. If they responded warmly, developing a connection was easy.

Consultant obstetrician HSI005: *“But most importantly I think it’s the personality, and the team were very keen to help. So she was very open with sending me all the [laughs] guidelines and even how to keep an audit, and I still actually once in four months catch up with her, so I would go to a Wednesday morning clinic and sit with her, and I’ve actually learnt a lot doing that…I think it was the fact that they were keen to help.”*

Once sites were in touch with others in their local network, many resources could be provided. These included more experienced sites offering their guidelines, direct help on specific clinical cases, introducing them to others/networking and helping them with shadowing/training to develop confidence in offering preterm surveillance care. Feeling supported by those with specialist preterm knowledge meant smaller units did not feel isolated, were able to develop confidence, and promoted less variations of care.

Programme Theory 7 highlights that even if clinicians have the clinical knowledge, expertise and skills, the pathway can be ineffective if they are not supported with adequate finances and resources. Staff at all sites commented on the pressure of lack of funding and resources in the current NHS [35–41].

Consultant obstetrician HSI002: *“It is difficult, the NHS, for all of us at the moment. It is a complete nightmare.”*

While they could undertake their job in the preterm clinic, it was a difficult place to work. Jobs took longer than they otherwise would, and/or staff stretched themselves to ensure the women did not miss out. Some felt this was compounded by the differing priorities of managers, meanwhile management felt little leeway in controlling resources to enable staff to feel supported.

### Using substantive theory to aid explanation of explanatory area 2

The Collective Action construct in Normalization Process Theory consists of four components (Interactional Workability; Relational Integration; Skill set Workability; and Contextual Integration) which illustrated the four programme theories for explanatory area 2 on the preterm birth surveillance clinic.

Interactional Workability is asking what the physical actions are taken by clinicians to perform the required task, and [23] and if clinicians can easily integrate the intervention or programme into their existing work [24]. An example in Programme Theory 4 demonstrated how it was harder to undertake the necessary requirements of the preterm pathway when there was not a core group of multidisciplinary preterm birth specialists.

Relational Integration is asking about what is undertaken by clinicians to work with colleagues and if they have confidence and trust their colleagues’ conclusions [23, 24]. Programme Theory 4 highlighted how when there was a core group of multidisciplinary preterm birth specialists, the team appreciated each other’s strengths, and trusted in each other’s work.

Skill set Workability questions if clinicians have the appropriate training, and therefore the appropriate skills, to support the intervention or programme [24]. Programme Theory 4 highlighted the importance of having a core group of multidisciplinary preterm birth specialists as they could then develop specialised knowledge and skills. Meanwhile Programme Theory 5 demonstrated the value of developing these specialised knowledge and skills, such as transvaginal scanning skills, as it enabled appropriate care planning.

Contextual Integration examines if there are sufficient resources to support the intervention or programme, and if management adequately support the intervention or programme [24]. Programme Theory 6 highlighted the importance of sites working together with their local network, and how resources (such as shadowing, developing skills, copies of protocols etc.) could then be provided to them. Meanwhile Programme Theory 7 emphasized the significance of ensuring the preterm birth pathway is supported by management with adequate finances and resources.

### Explanatory area 3: women centred care

Explanatory area 1 dealt with the problems in correct risk assessment and referral. Explanatory area 2 focused on how once a correct referral has been made, knowledgeable and supported clinicians can deliver a well-functioning clinic. Explanatory area 3 concentrates on ensuring how the pathway delivers appropriate care to the intended users it is trying to reach (women at high-risk of preterm birth) (Fig. 2).

Programme Theory 8 illustrated the benefits of continuity of care. If there is a small group of multidisciplinary preterm specialists, then women referred to the preterm clinic will have continuity of care, and the associated positive benefits.

Service user LWI001: *“…. she has been there consistently…so that has been reassuring that it is the same person every week. And we have built up a bit of a relationship.”*

These benefits included not needing to unnecessarily repeat their medical history, consistency (including clinicians utilising uniform language and devising consistent management plans), and an environment where openness was valued. Seeing familiar clinicians brought reassurance to woman that they were being cared and looked after.

Programme Theory 9 highlighted the importance of clinicians being aware and attentive to a woman’s history.

Service user LWI004: *“…you’re just not another pregnant mum, you’ve got a little bit of history and maybe you might need a little bit more [laughs] TLC [Tender Loving Care]…”*.

While regular clinical appointments with the specialist team may be helpful to women, it was not the regularity of appointments but the acknowledgment of their clinical history within those appointments that was important, as it ensured women did not feel neglected. It helped if women felt the clinicians had experience and a reputation of looking after those with a similar medical history.

Programme Theory 10 centred on clinicians acknowledging that women were experiencing a pregnancy at high risk of preterm birth but ensuring that this did not overshadow the ordinary aspects of their pregnancy.

*Consultant obstetrician LSO003 in observation 014: ‘… will talk about what’s normal for any mum. Sometimes it’s difficult when you had issues in the start of the pregnancy to then remember to focus on the usual pregnancy things.’*

If women are not solely defined by their ‘high-risk’ status, they felt they had agency over their care and choices available to them (such as exploring appropriate activities, such as aquanatal classes), meaning they felt more positive and confident in their pregnancy.

Service user LWI001: *“I always come away from my [preterm clinic] appointments feeling like I am just a normal pregnant person, even though everything that has happened….”*

Programme Theories 8,9 and 10 focused on ensuring a consistent overall baseline of care. Programme Theory 11 highlighted that clinicians require the time and freedom to provide flexible and individualised care.

Obstetric registrar LSI003: *“…gives us the freedom to individualise care …we’re quite keen to scan women quite frequently around the time that they lost their baby before or had an early [birth]… which certainly wouldn’t fit in…strict criteria.”*

If clinicians did not have this time and freedom, then appointments were rushed, and details not explained. In these cases, women had a more negative experience of the preterm clinic, were not sure what was the point of the appointment was, or their future care plan.

### Using substantive theory to aid explanation of explanatory area 3

The Health Foundation framework of four principles of person-centred care was based on an elderly hypothetical patient with numerous long-term conditions, however the parallels drawn helped inform the IMPART study results of explanatory area 3 on women at high-risk of preterm birth. When objective system data is collected (such as cost, or number of admissions [42]), our delivery systems are organised accordingly, meaning what matters to the individuals accessing care can be overlooked [43] and outcomes are prioritised.

Principle 1 involves *always* affording people dignity, respect and compassion [43]. This is easier for clinicians to achieve if they have time and freedom in preterm birth clinic appointments, which was seen in Programme Theory 11.

Principle 2 entails offering coordinated care, support or treatment. Someone should be responsible for coordinating a woman’s care, support or treatment [43]. If continuity of care is offered by the specialist preterm birth team, which is highlighted in Programme Theory 8, this can be easier to accomplish.

Principle 3 requires offering personalised care, support or treatment. This means treating the woman as a person, not a diagnosis, and paying attention to what matters individually to her [43]. This is echoed in Programme Theory 9, which focuses on clinicians being aware and attentive of a woman’s history to ensure aspects are not neglected.

Principle 4 concerns enabling. This term means the woman feels supported to develop her own unique range of capabilities [44]. Often hospitals focus on providing clinical services, when they should also focus on supporting women to recognise and build upon their own strengths to enable them to live an independent, fulfilling life [43]. This is highlighted in Programme Theory 10, which focuses on ensuring women are not defined by their high-risk status.

The Health Foundation framework of four principles provides person-centred activities to aid implementation of their principles. This has been utilised to aid interpretation and implementation of explanatory area 3, similarly to The Theoretical Domains Framework for explanatory area 1 and Normalization Process Theory for explanatory area 2.

## Discussion

Through using realist evaluation and substantive implementation science theory, the IMPART study has highlighted favourable conditions and recommendations for staff, the specialist preterm birth team, hospitals and external agencies [14].

Explanatory area 1 ‘risk assessing and referral’ highlighted the importance of clinicians having the appropriate knowledge. It is known that maternity risk assessments rely on clinicians having appropriate knowledge (which includes training, skills and competencies) to undertake them [45]. If that appropriate knowledge is not in place, as seen in the IMPART study, then it means they cannot be undertaken effectively.

Poor knowledge amongst clinicians has been found in other areas of maternity [46–49]. Studies demonstrate an improvement in knowledge in these areas after appropriate training [50, 51]. Including preterm birth in mandatory training and undergraduate teaching would improve this knowledge.

Even if clinicians have the correct knowledge, practical issues could still deter appropriate referral and risk assessment. Many of the practical issues relate to wider problems within the NHS, such as lack of funding and unpragmatic electronic systems. Maternity risk assessment systems that lack consistency and logic compromise clinicians’ ability in clearly communicating [45], which the IMPART study noted. Detrimental working environments and barriers for clinicians can lead to increased errors [31] and affect timely care [32].

Explanatory area 2 ‘the preterm birth surveillance clinic’ focused on what led to a well-functioning preterm birth clinic. NHS England’s Saving Babies Lives Care Bundle [7, 52] recommend that care is offered within a preterm clinic with access to screening tests including transvaginal cervical scanning. However, rather than just having *access* to scans (potentially undertaken elsewhere), the IMPART study demonstrates the benefits of transvaginal cervical scanning being undertaken by clinicians *within* the clinic. In England 19% of hospitals highlighted a lack of clinicians able to scan and/or lack of scanning equipment as hindering preterm clinic implementation [21]. Until more qualified midwives are trained, and/or RCOG includes transvaginal scanning in their curriculum [53, 54], this will be difficult to change.

The benefits that networks can provide when implementing a new pathway are well documented [55–57], and were echoed by the IMPART study and Saving Babies Lives Care Bundle [7, 52].

Clinicians highlighted the preterm clinic as being a difficult place to work with inadequate finances and resources, compounded by the differing priorities of managers. Clinician-manager discordance has been acknowledged elsewhere [58].

The IMPART study found that successful implementation was more likely if there was a core group of multidisciplinary preterm clinicians as they could develop concentrated knowledge and expertise. Multidisciplinary teams have been found to improve patient outcomes [59] and are actively encouraged [60, 61]. The IMPART study recommends that sites should not only having a named preterm consultant, but also a named preterm midwife [52] to ensure a strong multidisciplinary aspect. Developing concentrated knowledge and expertise has notable benefits such as encouraging the specialists to develop nuanced decision making, reducing variations of care, allowing others to develop preterm skills from them and to ask preterm questions and advice. It has been argued that care provided by specialist clinicians can become fragmented and less holistic [62]. Ensuring holistic, non-fragmented care was still provided was explored in explanatory area 3.

Explanatory area 3 ‘women centred care’ investigated antenatal continuity of care that can be offered by a multidisciplinary preterm team. This new contribution has not been studied elsewhere; however, midwifery continuity of care has been and is known to reduce preterm birth [63, 64]. Regularly attending a preterm clinic with the same multidisciplinary team may provide a relational continuity effect. Potential mechanisms for midwifery continuity of care have been explored elsewhere [65] and found similar results to the IMPART study (for example, women not repeating their medical history and open discussions, leading to women feeling cared for and safer).

Women at risk of preterm birth who experienced midwifery continuity of care welcomed the extra midwifery time offered when they required additional support [66]. Similarly, the IMPART study found that when clinicians have that flexibility and time, they are more likely to be able to provide individualised care and support.

Studies have highlighted how women feel supported if they receive specialist, enhanced antenatal care after perinatal loss [67, 68], such as that offered by a team of specialist multidisciplinary preterm birth clinicians. The IMPART study extends this research by demonstrating that clinicians should acknowledging a woman’s history during these appointments, rather than simply offering regular, specialist appointments.

While acknowledging a woman’s history is important, evidence has demonstrated the negative effects women experience through being labelled ‘at risk’ during their pregnancy [69–73]. Maternal stress and anxiety are independently associated with preterm delivery [74–76]. The IMPART study found that clinicians can mitigate this through ensuring a woman’s pregnancy is not solely focused on her increased preterm risk. Crucially, risk is dynamic [45]; a woman at high-risk of preterm birth could reach a term gestation and be considered low risk, highlighting the importance of ordinary aspects of her pregnancy not being overlooked.

Self-management support should be promoted in person-centred care. A review regarding pregnant women who have a chronic condition highlighted that women found the treatment shift towards self-management overwhelming; however, women’s anxiety mostly stemmed from insufficient knowledge and understanding of their diagnosed condition [77]. If they have supportive friends and family, alongside supportive clinicians, they are more motivated to undertake self-management [77]. Peer support is known to be helpful to women with vulnerabilities during pregnancy [78–80] and in those who have experienced pregnancy loss [81], however specialist antenatal classes for women who are experiencing similar pregnancies are rarely provided [82].

The more participation service users have in implementation, the more patient-centred that pathway becomes [83]. The IMPART study has gained insight into what is important for women, therefore clinicians and hospitals should ensure that this is included in their pathway, alongside continual, ongoing service user engagement [84]. Incorporating ‘Whose Shoes?’ events [85, 86]or similar in mandatory training could ensure woman-centred care is promoted [87].

The updated Saving Babies Lives Care Bundle V3 [47] was published in mid-2023 includes new recommendations that corroborate with the findings from the IMPART study. These include sites actively working with their local network, having a multidisciplinary preterm team (included a named preterm midwife), and recommending that this team should (have the freedom) to provide the oversight to pathway implementation.

**Fig. 3:**
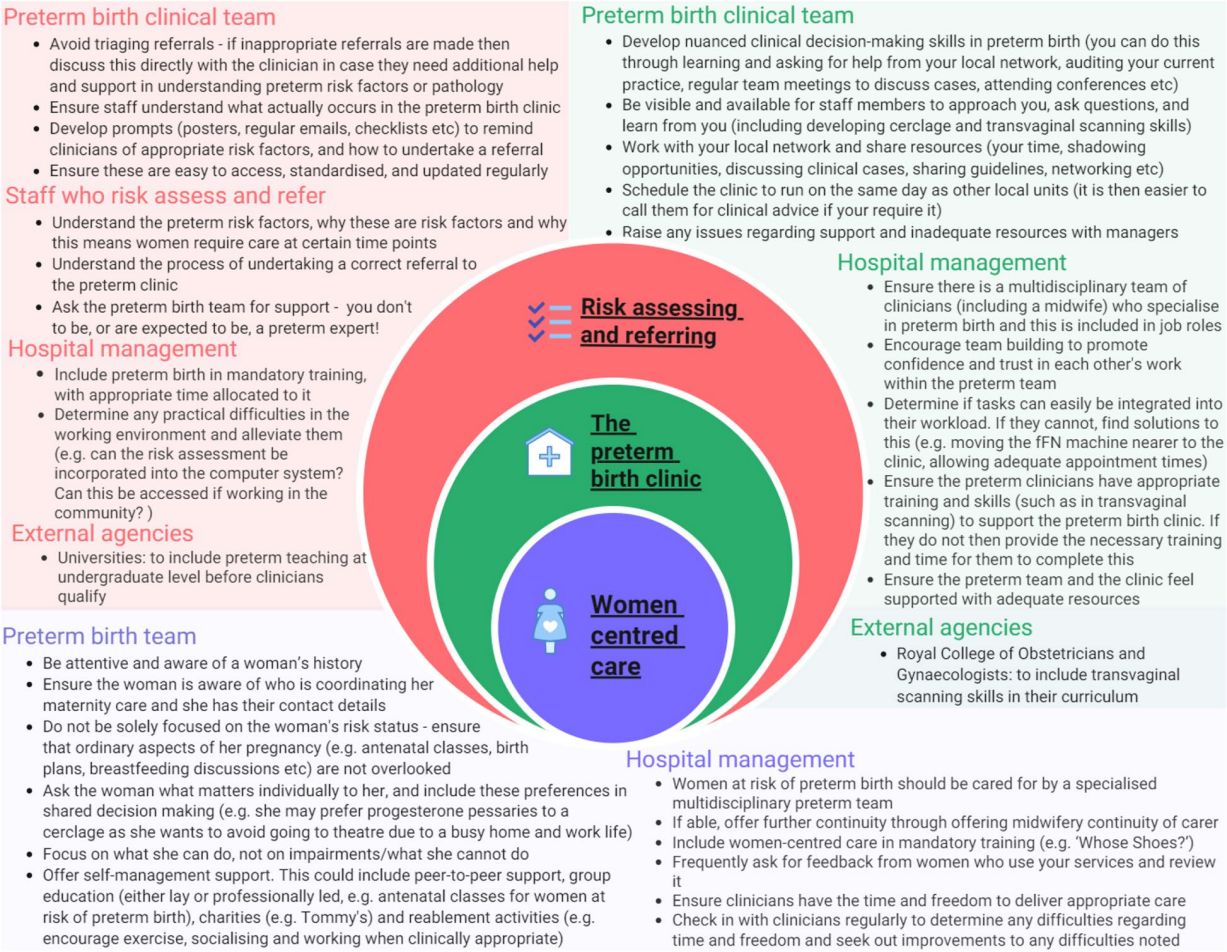
Infographic of the findings and recommendations from the IMPART study

A visual infographic was created to demonstrate how the findings from the three explanatory areas hung together to create clear recommendations for staff, the preterm birth multidisciplinary team, hospitals and external agencies on what can be undertaken to implement the preterm birth surveillance pathway effectively in their unit (Fig. 3).

### Strength and limitations

The IMPART study has identified the important areas to ensure optimal national implementation in the prediction and prevention aspects of the preterm pathway.

A strength includes the multiple methods (literature scope, interviews with national programme developers, and national questionnaire) utilised to develop the initial programme theories, combined with substantive implementation science theory. Interviews with those who developed the preterm element of Saving Babies Lives Care Bundle V2 ensured programme theories were created with awareness of their implementation vision.

While testing the programme theories occurred in only three case sites in England, the case sites were geographically spread, and represented a variety of maternity units, with varying preterm birth provisions serving a range of women demographically. The national questionnaire [21] utilised in developing the initial programme theories ensured a robust picture from a larger quantity of hospitals (96 units) was incorporated, before focusing on the three case sites, ensuring that the results of the IMPART study are transferrable nationwide.

The IMPART protocol [13] planned to also analyse routinely collected electronic data. While appropriate approvals were obtained (CAG reference: 21/CAG/0095), the data was not of sufficient quality, rendering it useless (e.g., unable to determine who had attended a preterm clinic). These issues have been highlighted by NHS Digital [88] and others [89].

## Conclusions

The IMPART study has provided several areas where implementation of the prediction and prevention aspects of Element 5 of the Saving Babies Lives Care Bundle could be improved. These include improving knowledge of risk factors and the purpose of the preterm surveillance clinic amongst clinicians. Sites should focus on training staff on preterm risk factors and pathology, while undergraduate midwifery courses should aim to integrate this into their teaching. Other areas to ensure optimal implementation include having a multidisciplinary preterm team (with a named preterm midwife) who have specialist preterm knowledge and skills (including transvaginal cervical length scanning skills), and sites actively working with their local network. This multidisciplinary preterm team are then placed to deliver continuity of care for women at high-risk of preterm birth, being attentive to their history but also ensuring they are not defined by their risk status.

## Supplementary Information

Supplementary Material 1.

Supplementary Material 2.

## Data Availability

Data analysis can be seen in Supplementary File 2. The transcripts from interviews and observational notes generated are not publicly available because consent to make data publicly available was not part of the consent by participants.

## Abbreviations

The IMPART study: IMplementation of the preterm birth surveillance PAthway – a RealisT evaluation
LMNS: Local Maternity and Neonatal System
NHS: National Health Service
RCOG: Royal College of Obstetricians and Gynaecologists
UK: United Kingdom
