# Correction: IMplementation of the Preterm Birth Surveillance PAthway: a RealisT evaluation (The IMPART Study)

**DOI:** 10.1186/s43058-024-00674-w

**Published:** 2024-11-29

**Authors:** Naomi Carlisle, Sonia Dalkin, Andrew H Shennan, Jane Sandall

**Affiliations:** 1 Department of Women and Children’s Health, The School of Life Course & Population Sciences, King’s College London, 10Th Floor North Wing, St Thomas’ Hospital, Westminster Bridge Road, London, SE1 7EH UK; 2 Faculty of Health and Life Sciences, Northumbria University, Newcastle Upon Tyne, UK

**Correction** **:** **Implement Sci Commun 5, 57 (2024)**

**https://doi.org/10.1186/s43058-024-00594-9**

The Original Article [1] contained incorrect Supplementary Materials due to typesetting errors. The original manuscript and a checklist were incorrectly processed as Supplementary Materials.

The correct Supplementary Materials are included with this Correction.

The Original Article has been corrected. The publishers apologize for this error and any inconvenience caused.

## Supplementary Information

Supplementary Material 1.

Supplementary Material 2.
