# Supplementary material for: IMplementation of the Preterm Birth Surveillance PAthway: a RealisT evaluation (The IMPART Study)
Source: Implement Sci Commun. 2024 May 21;5:57. doi: 10.1186/s43058-024-00594-9 (PMC11110199; doi:10.1186/s43058-024-00594-9)
Supplement: Supplementary file 1 — Supplementary Material 1. [file 43058_2024_594_MOESM1_ESM.docx]

# Supplementary file 1: Methods

### Developing initial programme theories

The realist literature scope, the interviews with the national level programme developers, and the national questionnaire all aided the formulation of the initial programme theories - which would then be ‘tested’ in the realist evaluation at the three case sites (in Stage 2).

Figure S1.1 Overview of the process undertaken to developing initial programme theories

### The initial programme theories to be tested

The initial programme theories, comprising of context, mechanism and outcome (C-M-O) configurations (see Table S1.1) were developed and ready to be ‘tested’ in the realist evaluation at the three case sites. They were utilised to refine the staff and women interview schedules and were applied when undertaking the observational analysis and analysing the routinely collected data.

Table S1.1 The initial programme theories to be tested, developed utilising Normalization Process Theory

| **Normalization Process Theory construct** | **Components of each construct** (1) | **Initial programme theories to be tested on staff** | **Initial programme theories to be tested on women** |
| --- | --- | --- | --- |
| NPT construct 1: sense-making (coherence)    Sense-making is the work that people  do individually and collectively when they are faced with the problem of operationalising some set of practices  ‘’What is it?’’ | - 1. **Differentiation**   Distinguish PTB pathway as discrete from other services  I can see how the [intervention] differs from usual ways of working | IF staff are given the time to undertake continual professional development training (education or exposure) to understand what the preterm pathway is and what it involves (C), THEN implementation of the pathway is more likely to be successful (O), BECAUSE staff will feel familiar and confident with distinguishing it from other services and therefore be aware of its distinct role (M) | IF women can access the information and are therefore aware of the different preterm resources provided at different hospitals (C), THEN they are likely to book their pregnancy at a hospital that has a good reputation for preterm care (even if this is not their nearest hospital) (M), BECAUSE they will want to everything in their power to ensure that they have the highest chance of a successful pregnancy (O). |
|  | **1.2** **Communal specification**  All agree about the purpose of the PTB pathway at their unit  Staff in this organisation have a shared understanding of the purpose of this [intervention] | IF leaders have taken the time to explain to staff so they are aware of why their site is implementing the preterm pathway and of its limitations (C), THEN implementation of the pathway is more likely to be successful (O), BECAUSE staff will be aware of the purpose of the pathway at their unit and feel satisfied with what is pragmatically possible (M) |  |
|  | - 1. **Individual specification**   Individually understand what the PTB pathway requires of them  I understand how the [intervention] affects the nature of my own work | IF the multidisciplinary team communicate with each other, staff will have knowledge of the pathway and feel confident in their role within the pathway (C), THEN implementation of the pathway is more likely to be successful (O), BECAUSE they are more likely to understand what responsibilities and functions are required of them to keep the pathway functioning effectively (M) | IF it has been communicated with women so they are aware of the process and purpose of the preterm pathway (C), THEN they will feel reassured and less anxious (O), BECAUSE when they attend appointments, they are more likely to understand without ambiguity what the service is trying to achieve (M) |
|  | **1.4 Internalization**  Construct potential value of the PTB pathway at their unit  I can see the potential value of the [intervention] for my work | IF staff have been given the resources to understand the advantages and benefits of the preterm pathway (C), THEN implementation of the pathway is more likely to be successful (O), BECAUSE staff will be more incentivised to implement something that has value and clear benefits for both them and patients (M) |  |
| NPT construct 2: buy-in (cognitive participation)  Buy-in is the relational work that people do to build and sustain a community of practice around a new technology or complex intervention  ‘’Who does it?’’ | **2.1** **Initiation**  Key individuals drive the PTB pathway forward  There are key people who drive the [intervention] forward and get others involved | IF sites have a motivated individual with dedicated time to be involved in implementation of the preterm pathway (C), THEN implementation of the pathway at that unit is more likely to be successful (O), BECAUSE that individual will take on the role of leading implementation and drive the pathway forward (M) |  |
|  | **2.2** **Enrolment**  Communal engagement to agree that the PTB pathway should be part of their work  I believe that participating in the [intervention] is a legitimate part of my role | IF staff have been involved in a two way dialogue with managers on how they need to reorganise the care that they deliver (C), THEN implementation of the pathway is more likely to be successful (O), BECAUSE staff will feel that they have a plan of what to follow in their role, to reach a clear goal (M) |  |
|  | **2.3** **Legitimation**  Commitment to the PTB pathway  I’m open to working with colleagues in new ways to use the [intervention] | IF staff have been involved in multidisciplinary discussions so they are aware of the contribution they can all make to the preterm pathway (C), THEN implementation of the pathway is more likely to be successful (O), BECAUSE they feel involved and therefore committed to the pathway (M) |  |
|  | **2.4** **Activation**  Continue to support the PTB pathway  I will continue to support the [intervention] | IF staff have communicated with each other so they are aware of what actions are needed to sustain and support the pathway (C), THEN implementation of the pathway is more likely to be successful (O), BECAUSE they will recognise when these actions are not occurring and then understand the required actions for a supported and sustained pathway (M) |  |
| NPT construct 3: doing (collective action)    Doing is the operational work that people do to enact a set of practices, whether these represent a new technology or complex healthcare intervention  “How does it get done?” | **3.1** **Interactional Workability**  Perform tasks required by the PTB pathway  I can easily integrate the [intervention] into my existing work | IF staff have had multidisciplinary discussions to recognize what tasks are required by the preterm pathway and how they can easily integrate them (C), THEN implementation of the pathway is more likely to be successful (O), BECAUSE they will be able to understand what tasks are not being undertaken and therefore correct this to ensure that they are performing these required tasks (M) |  |
|  | **3.2** **Relational Integration**  Maintain trust in each other's work and expertise in the PTB pathway  The [intervention] disrupts working relationships  I have confidence in other people’s ability to use the [intervention] | IF staff are given space to develop respect and trust in each other’s work and expertise (C), THEN implementation of the preterm pathway is more productive (O), BECAUSE as a multidisciplinary team they are more likely to understand each other’s, and therefore work to each other’s, strengths (M) | IF women are given the space to be offered individualised and holistic care (C), THEN they will feel reassured (O), BECAUSE their care will feel more personalised and inclusive, and they are therefore more likely to have trust in the clinician looking after them (M) |
|  | **3.3** **Skill set Workability**  The PTB pathway work is allocated appropriately  Work is assigned to those with skills appropriate to the [intervention]  Sufficient training is provided to enable staff to use the [intervention] | IF managers have the resources to ensure staff are allocated appropriate tasks (C), THEN implementation of the pathway is more productive (O), BECAUSE those staff members will have the correct skills to undertake their tasks effectively meaning they feel confident in their role (M) | IF women feel they have the resources so they can contact the right person at the right time (C), THEN they will feel confident and less anxious in their pregnancy (O), BECAUSE they will feel reassured in the knowledge that they can seek help whenever they require it (M) |
|  | **3.4** **Contextual Integration**  The PTB pathway is adequately supported by host organization  Sufficient resources are available to support the [intervention]  Management adequately support the [intervention] | IF management ensure that the preterm pathway is adequately supported with adequate finances and resources (C), THEN the pathway is more likely to be implemented successfully (O), BECAUSE staff will not have to worry about these periphery issues (such as funding, managerial support, time etc) and therefore can focus on delivering the pathway, feeling motivated and encouraged by their managers (M) | IF clinicians allow women remain in the clinic for emotional and mental support after they have been clinically discharged (C), THEN the service will be affected for other women who are coming through the clinic (O), BECAUSE resources of the clinic will be stretched too thinly (M) |
| NPT construct 4: appraisal (reflexive monitoring)  Appraisal is the monitoring work that people do to assess and understand the ways that a new set of practices affect them and others around them  “Why did it happen like that?” | **4.1** **Systematization**  Access information about the impact of the PTB pathway  I am aware of reports about the effects of the [intervention] | IF staff are given dedicated time to collate and monitor information about the preterm pathway (C), THEN the service will improve (O), BECAUSE they will be able to identify pinch points and current impacts of the pathway and therefore be better placed to address them (M) | IF the service actively asks women for their views, wishes and concerns about a service (C), THEN patient centred care will be promoted (O), BECAUSE the service can be developed/reshaped with their ideas which may not have previously been recognised alone by staff (M) |
|  | **4.2** **Communal appraisal**  Collectively assess PTB pathway as worthwhile  The staff agree that the [intervention] is worthwhile | IF staff have dedicated time to work together to assess the preterm pathway (C), THEN they can improve their service (O), BECAUSE they will be able to recognise what aspects are worthwhile collectively and what aspects require improvement (M) |  |
|  | **4.3** **Individual appraisal**  Individually assess the PTB pathway as worthwhile  I value the effects the [intervention] has had on my work | IF staff individually think and feel that the overall impact of the preterm pathway on them is beneficial (C), THEN implementation is more likely to be successful (O), BECAUSE they are likely to prioritise implementing a pathway that they consider to be worthwhile (M) |  |
|  | **4.4** **Reconfiguration**  Modify their work in response to appraisal of the PTB pathway  Feedback about the [intervention] can be used to improve it in the future  I can modify how I work with the [intervention] | IF staff feel comfortable to question modifying the preterm pathway as a result of their appraisals with their colleagues and managers (C), THEN implementation is more likely to be successful (O), BECAUSE they are likely to redefine the pathway to be more suitable and realistic for their hospital unit (M) |  |

Process and method of data collection

The mixed method data collection for Stage 2 (from the three case sites to test the initial programme theories developed during Stage 1 and seen in Table S1.1) included:

- Interviews with staff receiving care at the three case sites.
- Interviews with women receiving care at the three case sites.
- Observational analysis of staff working at the three case sites.
- Observational analysis of women receiving care at the three case sites.

Participants are chosen based on their ‘CMO investigation potential’ (2).

### Data analysis

As data collection from the three sites progressed, it became clear that the initial programme theories developed utilising Normalization Process Theory were not fully fitting the data. An online whiteboard ([www.miro.com](http://www.miro.com)) with sticky notes was created to help visually map what the data were illustrating. This helped protect against ‘theory-induced blindness’ (3–5), where data is disregarded as it does not fit initial assumptions (6). The online whiteboard can be seen in Figure S1.2 below, where the red tabs in the bottom of the sticky notes correspond to the initial programme theories developed utilising Normalization Process Theory.


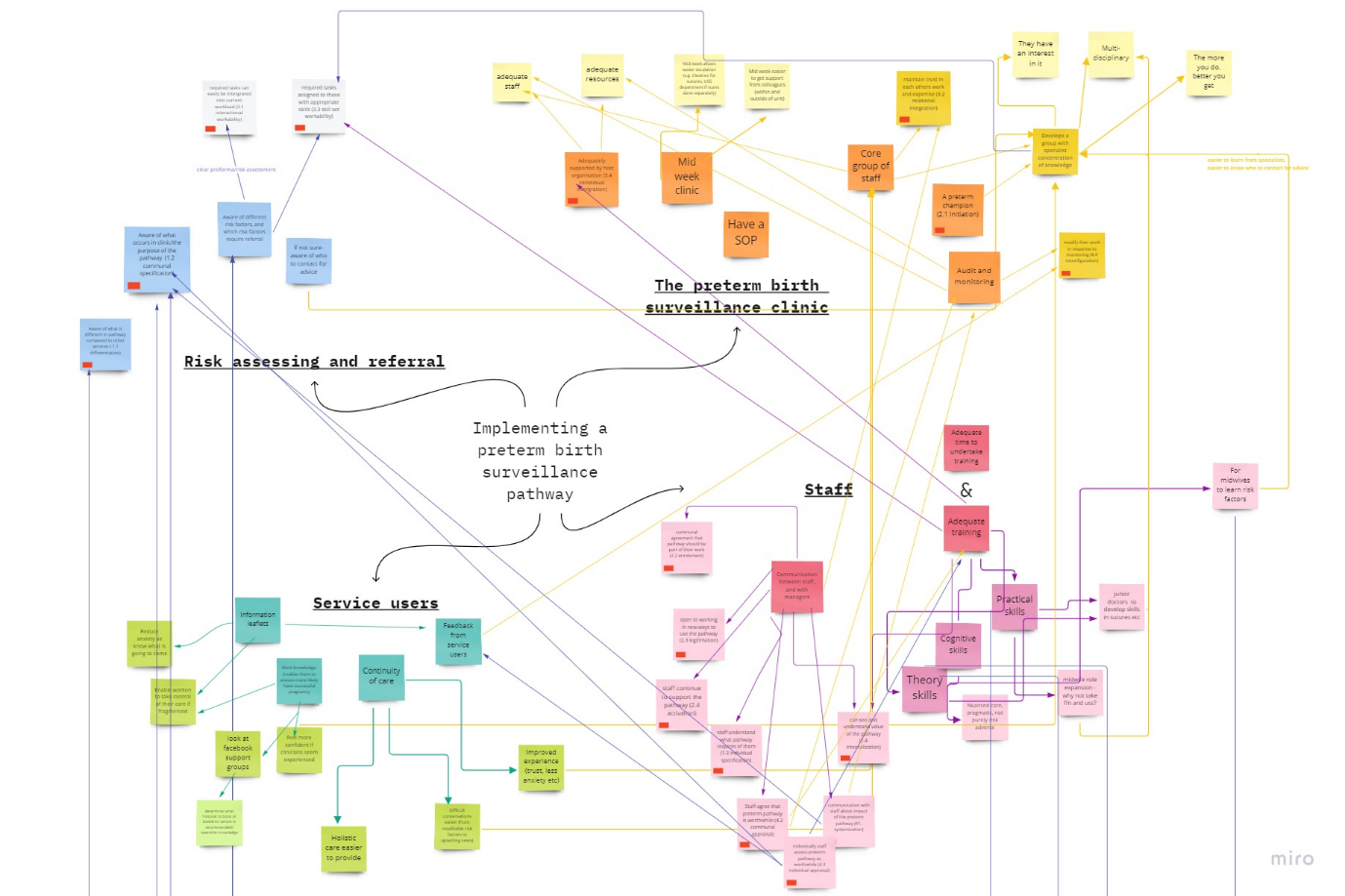


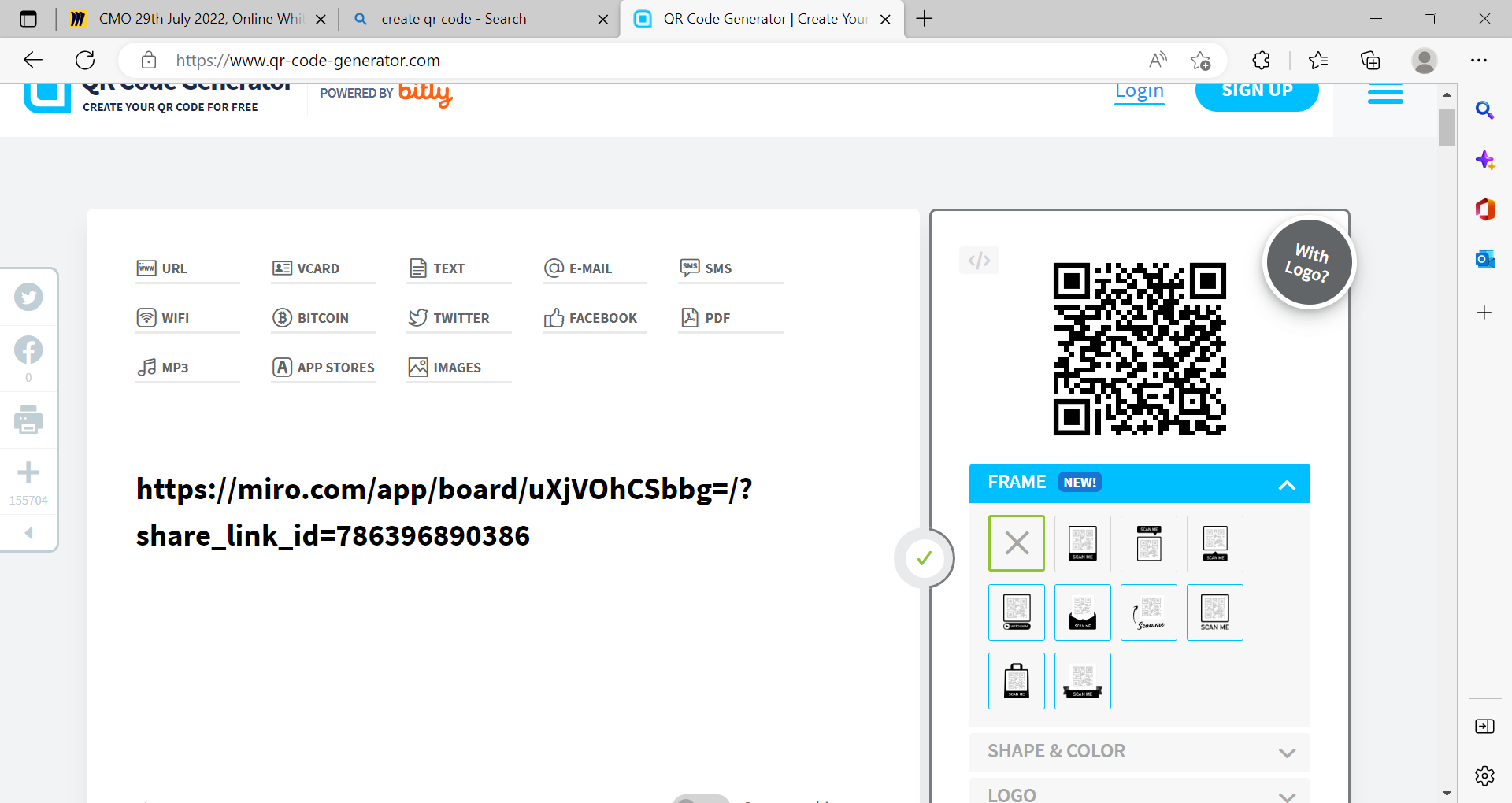


Figure S1.2 Whiteboard 29th July 2022

To view in detail please go to: <https://miro.com/app/board/uXjVOhCSbbg=/?share_link_id=786396890386>

Or scan this QR code:

The online whiteboard helped illustrate that while Normalization Process Theory was moderately explaining the data, it was not entirely explaining the data. As data collection progressed, the online whiteboard evolved to create more sticky notes and NVivo codes (denoted by cyan tabs in the corner of the sticky notes). These additional NVivo codes reflected initial programme theories that had been developed prior to data collection but were initially decided to not be taken forward for testing through data collection. However, the data was demonstrating that these were important, so these initial programme theories were then taken forward for testing in the three case sites. Using a combination of initial programme theories developed prior to data collection guided by Normalization Process Theory, and initial programme theories developed prior to data collection that were not guided by Normalization Process Theory, the data were fully explained.

As data collection continued, the online whiteboard was continually updated iteratively to reflect what the data was articulating. A later snapshot of the online whiteboard can be seen in Figure S1.3 and Figure S1.4, to demonstrate this change against the earlier Figure S1.2. As a specialist midwife in preterm birth, reflexivity was considered throughout data analysis (7).


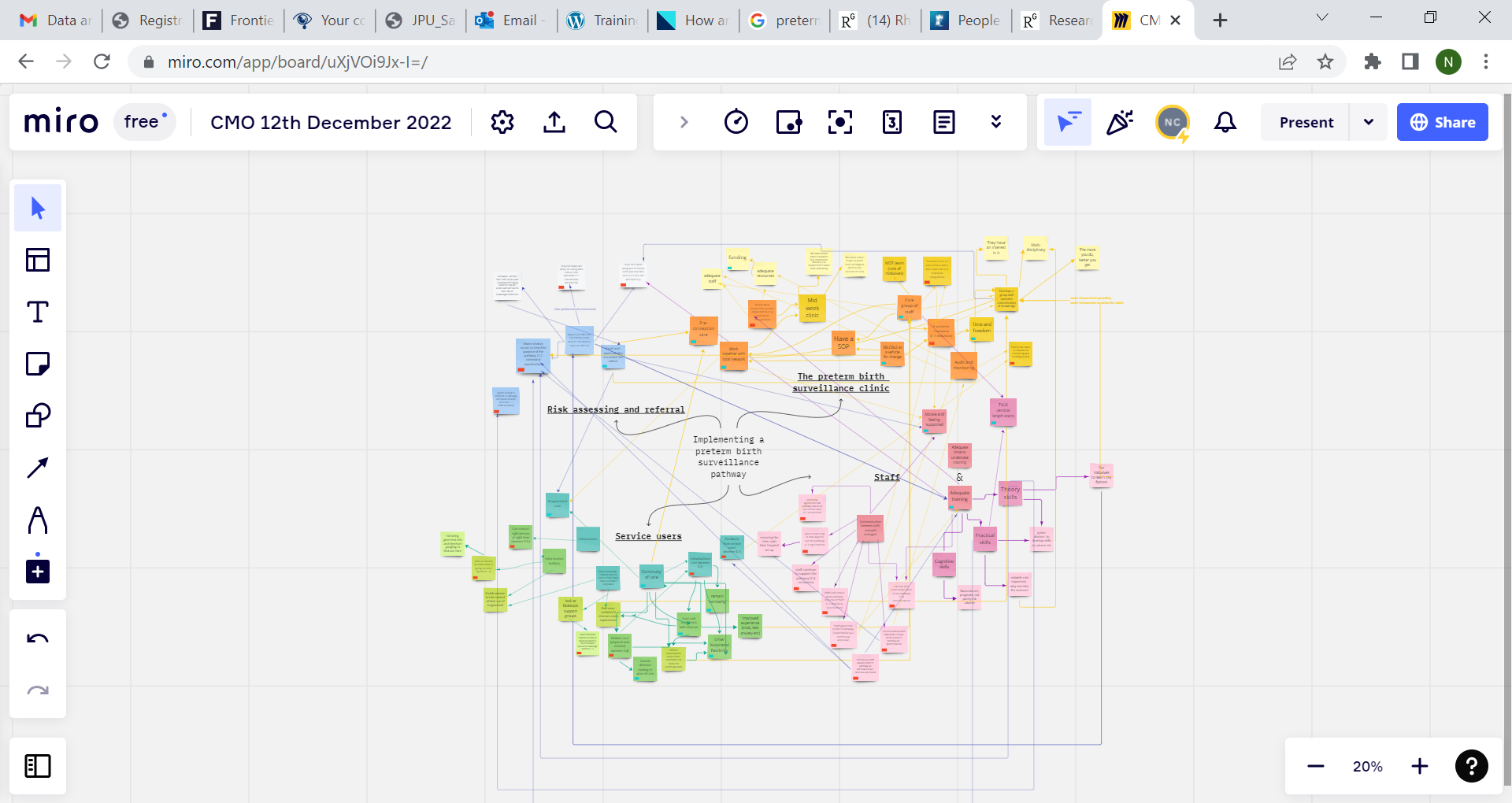


*Figure 3 Whiteboard 12^th^ December 2022*


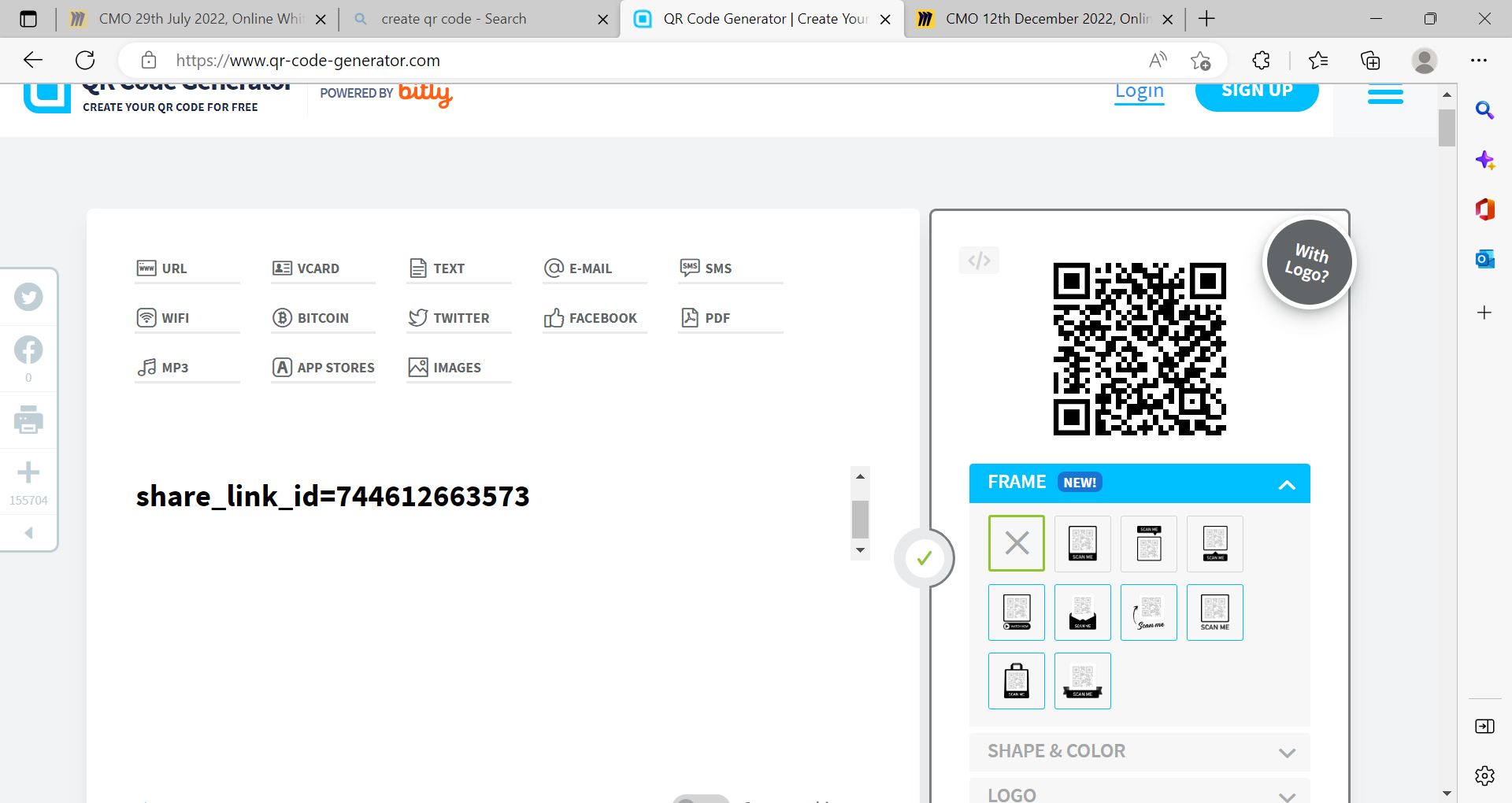


To view in detail please go to: <https://miro.com/app/board/uXjVOi9Jx-I=/?share_link_id=744612663573>

Or scan this QR code:


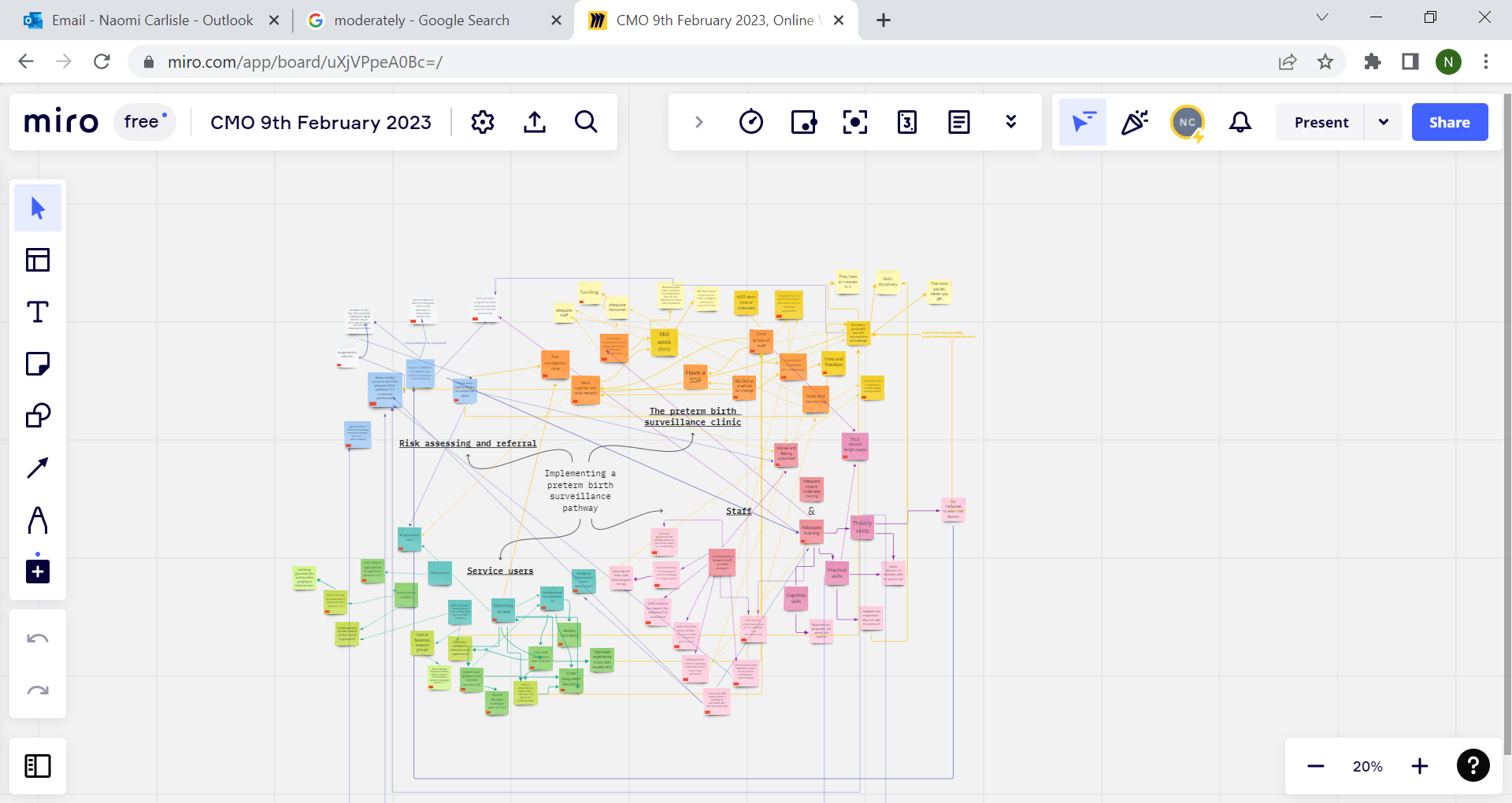


*Figure 4 Whiteboard 9^th^ February 2023*


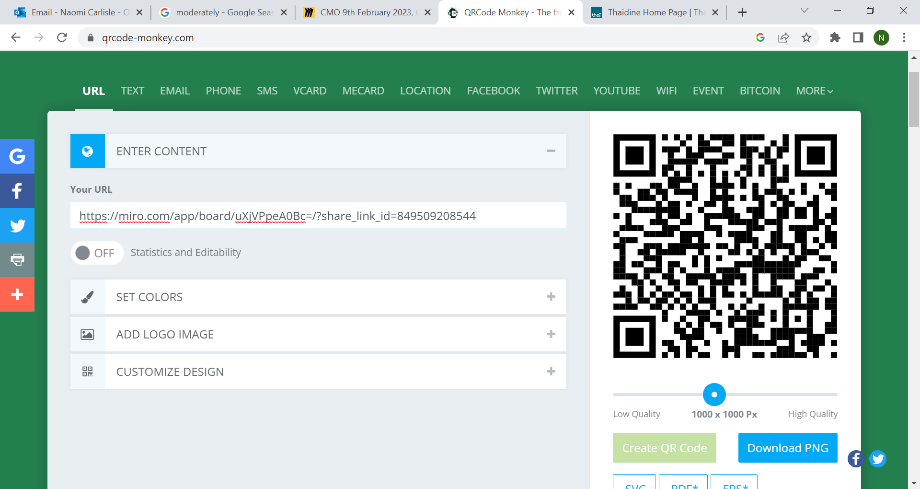
To view in detail please go to: <https://miro.com/app/board/uXjVPpeA0Bc=/?share_link_id=849509208544>

Or scan this QR code:

The online whiteboard helped to organise the data into four explanatory areas: risk assessing and referral; the preterm birth surveillance clinic; staff; service users. While undertones of Normalization Process Theory were embedded within each of these areas, organising the data as these areas rather than as a Normalization Process Theory format achieved greater coherence.

Over time, these merged into three areas: risk assessing and referral; the preterm birth surveillance clinic; women centred care. Staff members do not have their own separate area, as they are interlaced throughout all three areas.

## References

1. Finch TL, Girling M, May CR, Mair FS, Murray E, Treweek S, et al. Improving the normalization of complex interventions: part 2 - validation of the NoMAD instrument for assessing implementation work based on normalization process theory (NPT). BMC Med Res Methodol. 2018;18(1):135.

2. Pawson R, Tilley N. Realistic Evaluation. London: Sage; 1997.

3. Westhorp G. Development of realist evaluation models and methods for use in small-scale community based settings [Internet]. Nottingham Trent University; 2008 [cited 2023 Jan 31]. Available from: https://ethos.bl.uk/OrderDetails.do?uin=uk.bl.ethos.493092

4. Kahneman D. Thinking, fast and slow [Internet]. 1st ed. New York : Farrar, Straus and Giroux, [2011] ©2011; 2011. Available from: https://search.library.wisc.edu/catalog/9910114919702121

5. Howson C. Fitting Your Theory to the Facts: Probably Not Such a Bad Thing after All. In: Savage CW, editor. Minnesota Studies in the Philosophy of Science. Minneapolis: University of Minnesota Press; 1990. p. 224–44.

6. Haynes A, Gilchrist H, Oliveira JS, Tiedemann A. Using Realist Evaluation to Understand Process Outcomes in a COVID-19-Impacted Yoga Intervention Trial: A Worked Example. Int J Environ Res Public Health. 2021;18(17).

7. Burns E, Fenwick J, Schmied V, Sheehan A. Reflexivity in midwifery research: The insider/outsider debate. Midwifery [Internet]. 2012 Feb 1 [cited 2019 Apr 13];28(1):52–60. Available from: https://www.sciencedirect.com/science/article/pii/S0266613810001798
