# Supplementary material for: IMplementation of the Preterm Birth Surveillance PAthway: a RealisT evaluation (The IMPART Study)
Source: Implement Sci Commun. 2024 May 21;5:57. doi: 10.1186/s43058-024-00594-9 (PMC11110199; doi:10.1186/s43058-024-00594-9)
Supplement: Supplementary file 2 — Supplementary Material 2. [file 43058_2024_594_MOESM2_ESM.docx]

# Supplementary file 2: Data analysis and results

# Explanatory area 1: Risk assessing and referral

*Table S2.1 I**PT and questions asked of the data for 'risk assessing and referral'*

| **Explanatory**  **area** | **Programme Theory** | **Nodes incorporated** | **Original IPT(s)** | **Subsidiary questions** | **Refined Programme Theory** |
| --- | --- | --- | --- | --- | --- |
| Risk assessing and referral | Programme Theory 1: Knowledge of risk factors | - Aware of different risk factors and which risk factors require referral - Midwives’ knowledge - Training and education - Required tasks assigned to those with appropriate skills (3.3 skill set workability) - If not sure, aware of who to contact for advice - Inappropriate referrals | IF managers have the resources to ensure staff are allocated appropriate tasks (C), THEN implementation of the pathway is more productive (O), BECAUSE those staff members will have the correct skills to undertake their tasks effectively meaning they feel confident in their role (M) | 1. How do you think midwives are aware of the different risk factors and what ones require referral? 2. How do you think midwives feel in terms of confidence in risk assessing women? 3. In your opinion, have they had appropriate training on this? 4. Could you explain who you would contact for advice? | Refined Programme Theory 1 after data collection: IF leaders ensure staff have sufficient training and education when allocated the task of risk assessing and referring women (C), THEN appropriate referral to the preterm pathway is more likely (O), BECAUSE staff members will have the correct knowledge to be aware of which risk factors require referral and/or know where to gain this information (M). |
|  | Programme Theory 2:  Knowledge of pathology | - Aware of what is different in the pathway compared to other services (1.1 differentiation) - Aware of what occurs in the clinic/the purpose of the pathway (1.2 communal specification) | IF staff are given the time to undertake continual professional development training (education or exposure) to understand what the preterm pathway is and what it involves (C), THEN implementation of the pathway is more likely to be successful (O), BECAUSE staff will feel familiar and confident with distinguishing it from other services and therefore be aware of its distinct role (M)  IF leaders have taken the time to explain to staff so they are aware of why their site is implementing the preterm pathway and of its limitations (C), THEN implementation of the pathway is more likely to be successful (O), BECAUSE staff will be aware of the purpose of the pathway at their unit and feel satisfied with what is pragmatically possible (M) | 1. How do you think midwives understand what occurs in the clinic? 2. Could you talk to me about what you think midwives understand about the purpose of the clinic? | Refined Programme Theory 2 after data collection: IF leaders have educated staff on the pathology of preterm birth (C), THEN appropriate referral to the preterm pathway is more likely (O), BECAUSE staff will be aware of what is provided to women in preterm birth surveillance clinics at different timeframes and why this is important (M) |
|  | Programme Theory 3:  Practical  difficulties | - Required tasks can easily be integrated into current workload (3.1 interactional workability) | IF staff have had multidisciplinary discussions to recognize what tasks are required by the preterm pathway and how they can easily integrate them (C), THEN implementation of the pathway is more likely to be successful (O), BECAUSE they will be able to understand what tasks are not being undertaken and therefore correct this to ensure that they are performing these required tasks (M) | 1. In your opinion, how could midwives practically risk assess and refer women? | Refined Programme Theory 3 after data collection: IF recognition was given to the practical difficulties midwives undergo to refer women (C), THEN easy and appropriate referral to the preterm pathway is more likely to be successful (O), BECAUSE support and solutions can then be found to easily incorporate the required tasks into their workload (M) |

**Programme Theory 1 – Knowledge of risk factors**

Original IPT: IF managers have the resources to ensure staff are allocated appropriate tasks (C), THEN implementation of the pathway is more productive (O), BECAUSE those staff members will have the correct skills to undertake their tasks effectively meaning they feel confident in their role (M)

Refined Programme Theory 1 after data collection: IF leaders ensure staff have sufficient training and education when allocated the task of risk assessing and referring women (C), THEN appropriate referral to the preterm pathway is more likely (O), BECAUSE staff members will have the correct knowledge to be aware of which risk factors require referral and/or know where to gain this information (M)

In the refined Programme Theory 1, sufficient resources have been omitted to focus on knowledge of risk factors. However, the starting point should be whether staff are aware of how to assess and refer, then whether they have the resources to do so. Resource allocation and practical difficulties have therefore been explored, later in this chapter, during Programme Theory 3.

As data collection began it became clear that the original IPT (which questioned whether staff were allocated appropriate tasks) was not quite asking the right starting question. Even if risk assessing was deemed an appropriate task to be allocated to a midwife, if the midwife did not know or understand the risk factors, then this stage could not be implemented effectively. This was highlighted in early interviews (for example in the second interview undertaken for the IMPART study with participant HSI003 below), and led to the abductive development of the subsidiary questions seen in Table 1 when collecting subsequent data.

Midwife HSI003: *“I feel like sometimes I say things to women, and I honestly don’t know why I say it or the real rationale behind it, so I book a woman with like you say that bicornuate uterus or the LLETZ, and I’ll be like “I need to refer you to the Preterm Clinic”…”*

Abductive reasoning has been described as beginning “with an incomplete set of observations and proceeds to obtaining the likeliest possible explanation for the set” (Mukumbang, 2020, p. 6). In this case, Midwife HSI003 highlights that she does not understand the preterm birth risk factors, but the subsidiary questions helped define potential explanations for why this is. Retroduction, while analysing the data iteratively throughout, led to the refined theory.

The subsidiary questions were not utilised in every interview. Firstly, as they were developed with iterative data analysis, it meant some interviews would have been undertaken already before they were developed. Secondly, different programme theories require different respondents, so it may not have been appropriate or worthy to ask all respondents (Manzano, 2016; Pawson & Tilley, 1997). Finally, the subsidiary questions were asked in different formats to different respondents (as in, while the general gist may have been the same, the exact wording and phrasing was not). This was because the interviews were realist, semi-structured interviews, and therefore not conducted with a structured interview guide (Manzano, 2016)

Mechanism: Knowledge of risk factors

Across all three sites, a lack of midwifery knowledge on preterm birth risk factors was noted by midwives themselves, other members of the multidisciplinary team, and women.

In observing booking appointment 059 (where the woman being booked had a history of two previous first trimester miscarriages which as they were in the first trimester are not a risk factor for preterm birth), midwife HSO002 reviewed her notes before calling the woman into the room to begin the appointment and said to me: *I’m aware you’d refer to prem clinic if she had more than 3 miscarriages, so she doesn’t need referral.*

*

Interviewer (Naomi Carlisle): *“So, you had this early scan and then I am guessing you booked your pregnancy with the midwife and is that when you let her know about the bicornuate uterus?”*

Service user HWI001: *“Yeah. Yeah.”*

Interviewer: *“Yeah. Okay. And when you explained to her that you had this bicornuate*

*uterus, did she…”*

HWI001: *[sighs] “I do not think she had a clue what it was” [laughter].*

Interviewer: *“Ah okay. Interesting.”*

HWI001: *“Because I was like, ‘I have a bicornuate uterus.’ She was like, ‘Yeah. Yeah. Whatever. It is fine.’ And then, a few weeks later, she called me and she was like, ‘Oh, I signed you up for the preterm. They told me something, something. You need to come’”*

*

Midwife HSI003: *“…if you don’t read especially the preterm referral form, you wouldn’t even know that these things exist. The LLETZ, as you were saying, I believe it’s not just the LLETZ, it’s like I don’t know, because I remember referring a lady for that, and then the response was, well, I don’t know, it was something about the surgery and the type or whatever, and she didn’t need it.”*

Midwife HSO002 was therefore unaware that for a correct referral to preterm clinic, the risk factor associated with miscarriage would be the gestation that the miscarriage occurred at, not the frequency. Meanwhile midwife HSI003 knew that having a LLETZ procedure was a risk factor for preterm birth, but the nuances (around the frequency of procedures, and the depth of procedures) that warrant a valid referral were lost. The interview with HWI001 demonstrated a situation where the midwife who booked her was therefore initially unaware that a bicornuate uterus was a risk factor for preterm birth until hurriedly contacting her a few weeks later (when presumably this being a risk factor had been highlighted to her from another member of staff).

Even if midwives knew what the ‘tick box’ risk factors were that required referral, they did not know the aetiology behind why they required referral.

Consultant obstetrician HSI002: *“I would teach the midwives. I would make it mandatory for the midwives, once a month, an hour, just to understand what LLETZ is. Because they know these concepts, but they don’t… If you said, ‘What does LLETZ stand for?’ they will go, ‘It is a cervix thing, right?”*

***

Midwife YSO003 in Observation 076*: I only know of procedures like LLETZ because I had a LLETZ personally myself…otherwise I don’t really know why it’s important*

***

Lead midwife for antenatal clinic and day care YSI003*: “I know, especially when we have to get people referred for like, a section at full dilatation, I had a lot of midwives coming and saying, “Well, why? What implication has that got?” So I think the knowledge isn’t quite there”.*

Despite not being aware of the aetiology, participants thought it was important that they were aware of this knowledge so they would feel confident explaining the risk assessment to women.

Midwife YSO004 in Observation 077: *Being honest…no I wouldn’t know why a fully dilated c-section makes you high risk.*

When asked midwife YSO004 if it was important that she knew the aetiology behind this: yes*…because otherwise how do I explain to the woman if she asks me questions?*

***

Midwife HSI003: *“It definitely would improve our knowledge and even let us be a bit more confident when we relay a message to our clients.”*

While the focus here is on midwives as their risk assessment at booking and correct referral forms the start of the preterm pathway, it was noted how this lack of knowledge extended to junior obstetricians too.

Consultant obstetrician HSI002: *“I have had junior registrars who don’t know the difference between a LLETZ and a cone biopsy.”*

***

*Obstetric registrar LSI003: “And I think that’s with junior doctors as well, they see someone that’s had a 32-week delivery and think oh she needs to be seen in prem clinic but what they fail to realise is this baby was severely growth restricted and had a really poor placenta and actually we have a different pathway that that woman would benefit from rather… and it was a planned section for severe growth restriction.”*

Context: Sufficient training and support

Insufficient training was blamed for a lack of midwifery knowledge. This started with a lack of education at midwifery undergraduate level (before becoming qualified). One [HSO004] felt that being a nurse prior to becoming a midwife (rather than a direct-entry midwife) mitigated this somewhat due to their broader gynaecological and obstetric (rather than just obstetric) knowledge.

Consultant obstetrician LSI004: *“…we’ve failed to educate a whole cohort of midwives. And I work for HSIB [Healthcare Safety Investigation Branch] and I know that’s a national problem. And it’s the same with prematurity teaching, I think we have forgotten… And I don’t know if part of that is midwifery education at the university - do we so focus on normality? So I was asked to teach. I was actually doing a talk on prematurity, so prediction/prevention and treatment to York student midwives and they were like, “Oh this is amazing, we never get taught by an obstetrician, we never learn about this sort of thing,” and I’m like how can you not…? Afterwards I was thinking how can that not happen? I call it midwife school. Has midwife school almost forgotten about actually midwives, a lot of the time now they are looking after high-risk women and we can’t just allow them to focus on the normality.”*

***

Preterm birth midwife LSI001: *“I'm a direct-entry midwife, and I went what is a LLETZ? I don’t even know what that is. [laughter] … there is a lack of knowledge in a lot of areas… When I started the role [as a specialist preterm birth midwife] I realised how much I didn’t know, even about the pre-term birth stuff.”*

***

Midwife HSO004 in Observation 065: *Explained that as she was a nurse before becoming a midwife that she knew about what a LLETZ was and other gynae procedures were. She did not feel she had to learn all of that when the preterm birth guidelines came out.*

***

Midwife YSI003*: “I think putting the basics out there at unis and stuff because I don’t remember doing anything about stuff like that.”*

***

Midwife YSO004 and Midwife YSO005 in Observation 077*: Both felt preterm risk factors were not taught at university, or in mandatory training since.*

Insufficient training was also noted in a lack of mandatory training or post qualification updates.

Maternity care assistant HSI001: *“…it’s relatively new in the hospital so even for the midwives, sometimes I think it’s a bit of a grey area, they’re not really sure whether they should refer the patient to this clinic or they shouldn’t do it. So yes, there’s still a lot to learn about it, I think.”*

***

Consultant obstetrician HSI002: *“I think no one is teaching them [midwives]. I don’t know what it is like in your hospital, but in our hospital, midwives have a three-day mandatory training once a year. During those three days…it is about breastfeeding and carbon monoxide monitoring, all of that stuff. There is no mandatory training at all for them to learn anything. They don’t have a logbook. They don’t have to go to a webinar. There is nothing.”*

Some staff felt that the reason this training in preterm birth did not occur was due to time.

Midwife HSI003: “*We definitely need more time. It’s like one of the biggest issues, and even part of the Ockendon report on our visit was because we’re not getting time to train, to develop ourselves, we’re just learning as we go along.”*

***

Midwife HSI004: *“I do not think all midwives know about it [preterm risk factors] and because there is such a paucity of training and actually, you can barely get enough time to do your MMT [Mandatory Maternity Training], let alone a luxury of supplementary learning about things like semi-obscure gynaecological procedures.”*

Outcome: Appropriate referral more likely

Refined Programme Theory 1 after data collection: IF leaders ensure staff have sufficient training and education when allocated the task of risk assessing and referring women (C), THEN appropriate referral to the preterm pathway is more likely (O), BECAUSE staff members will have the correct knowledge to be aware of which risk factors require referral and/or know where to gain this information (M).

Two observations illustrating this full Context-Mechanism-Outcome triad are below:

Midwife LSO005 in Observations 079-082: *Booked service user LWO055 who had a previous spontaneous preterm birth at 21 weeks and 5 days the previous year. Midwife LSO005 did not initially refer her to a preterm birth clinic (OUTCOME), as she explained that she thought* ‘*it is a difficult gestation 21+5…and I am not even sure preterm clinic would see her because it’s too early isn’t it? Too early for preterm?’ (MECHANISM). Midwife LSO005 explained that she did not feel confident in knowing preterm birth risk factors as ‘there’s so much to know… you end up feeling comfortable with just not knowing stuff’, being newly qualified she felt she hadn’t ‘been taught any of it, especially not at university’ (CONTEXT).*

*

Midwife HSO004 in Observation 065: *Explained that as she was a nurse before becoming a midwife that she knew about what a LLETZ was and other gynae procedures were* (CONTEXT). *She therefore did not feel she had to learn all of that when the preterm birth guidelines came out* (MECHANISM). *Midwife HSO004 in Observation 068 did not refer service user HWO025 to the preterm birth clinic as knew that her endometrial cyst was not a risk factor for preterm birth (OUTCOME).*

When midwives were unsure what risk factors warranted referral, they sometimes undertook a ‘scattergun approach’ to referrals to err on the side of caution.

Midwife YSO004 and midwife YSO005 in Observation 077*: Both felt preterm risk factors were not taught at university, or in mandatory training since (CONTEXT). They said they would therefore not know if risk factors were risk factors for preterm birth (MECHANISM) but would know that these risk factors made them outside of normal. They would therefore refer these women to the obstetric team anyway, knowing that eventually the woman would wind up with the correct additional care (OUTCOME).*

***

Lead midwife for antenatal clinic and day care YSI003: *“I’ve always said it’s better to refer and then we don’t need to. So quite often they might just put a little comment on the referral form like, ‘query needs consultant, query this,’ just so that we know they are not really sure and then we can make that judgement.”*

***

Midwife YSO003 to service user YWO004 in Observation 075*: I will refer you and if its wrong they can refer you back.*

Midwife YSO003 later explained in Observation 075 that she: *would prefer to refer a woman to obstetrics and if it is wrong for the referral to be sent back, rather than get something missed.*

While the ‘scattergun approach’ to referrals initially seems harmless, it creates additional workload later for those screening through the appropriate and inappropriate referrals. It also demonstrates the lack of knowledge from those referring, meaning the referral process is flawed and open to error, leaving women vulnerable to not receiving appropriate care. This was demonstrated in an interview with the superintendent sonographer. At this particular site, the transvaginal ultrasound scans to measure the cervical length were always performed by the sonography department. Some of these cervical length scans were undertaken at the same time as the anomaly scan to not inconvenience the woman by attending two separate appointments. However, if this need for an additional scan was not flagged by the midwife at the booking appointment, it caused admin issues, despite the woman receiving the appropriate care in the end.

Superintendent sonographer YSIOO2: *“…if we could know in advance that that was likely, from their booking history, then it would be far easier to make the two together, because we do have to add the extra 15 minutes onto our anomaly scan, we can’t just incorporate it into the scan. And then because they’re booked in half hour slots, we then have to take somebody out and then push somebody back. So there’s a lot more admin side. Because if we knew in advance, and I think the majority of cases we would and should know in advance, then that would be helpful. … Because as I say, a card will follow on to say this is required, but we don’t know about it at that time of booking and then have to go back and adjust. I would say the vast majority, we don’t know”*

**Programme Theory 2 – Knowledge of pathology**

Original IPT: IF staff are given the time to undertake continual professional development training (education or exposure) to understand what the preterm pathway is and what it involves (C), THEN implementation of the pathway is more likely to be successful (O), BECAUSE staff will feel familiar and confident with distinguishing it from other services and therefore be aware of its distinct role (M)

Original IPT: IF leaders have taken the time to explain to staff so they are aware of why their site is implementing the preterm pathway and of its limitations (C), THEN implementation of the pathway is more likely to be successful (O), BECAUSE staff will be aware of the purpose of the pathway at their unit and feel satisfied with what is pragmatically possible (M)

Refined Programme Theory 2 after data collection: IF leaders have educated staff on the pathology of preterm birth (C), THEN appropriate referral to the preterm pathway is more likely (O), BECAUSE staff will be aware of what is provided to women in preterm birth surveillance clinics at different timeframes and why this is important (M)

During data collection it soon became apparent that rather than staff just being aware of *what* preterm birth risk factors were*,* they also needed an understanding of the pathology of preterm birth, to understand *why* particular care was offered at certain timeframes.

*Consultant Obstetrician HSI002: “I don’t think they even know what happens in it [the preterm birth surveillance clinic]”*

This subtle difference was important. Staff could be aware that the purpose (the *what*) of referring to the preterm surveillance clinic was to reduce preterm birth. However, by understanding the pathology (the *why)*, they could then understand the purpose and processes for correct referral. For example, if they understood that the clinic involved assessing cervical length then they would be aware of why a previous spontaneous preterm birth would be an appropriate referral, but a previous iatrogenic preterm birth would not be. Developing the refined Programme Theory 2 involved the merge of the IPTs associated with two of the first Normalization Process Theory constructs for ‘sense-making’ (1.1 differentiation and 1.2 communal specification).

Mechanism: Purpose of the pathway known

One consultant obstetrician felt that the lack of knowledge on the purpose of the preterm clinic was not limited to midwives, but extended to obstetricians and those from other units.

Consultant Obstetrician LSI004: *“…when I get referrals in from elsewhere, it’s very clear that they have no idea why this woman has delivered preterm before. They don’t understand.”*

Consultant obstetrician LSI004 goes on to explains how she feels this lack of knowledge on the pathology of preterm birth (CONTEXT) affected midwives’ ability to refer effectively (OUTCOME). In this case she explains that the mechanism behind this is because midwives acknowledge a woman is high risk, but struggle to differentiate between two specialist services (the rainbow clinic and the preterm surveillance clinic) and what these services can provide to women (MECHANISM).

*Consultant obstetrician LSI004: “…if it’s barn door placental pathology and absolutely no cervical pathology, then we will try and bypass that, but I don’t think midwives understand that. Likewise, I’ve had… so we have a rainbow clinic which is for previous stillbirths and I have a woman that had preterm rupture of membranes at 22 weeks and then she had a still birth at 25 weeks and they are referring to the rainbow clinic and not the prem clinic or they refer to both and I’m like, “Well we’ll look after in prems because the reason why the baby died is because of a prematurity issue not a stillbirth”. So there’s that sort of mix-up, if you know what I mean. I suppose what I’m saying is they don’t truly understand the pathology of preterm birth. So they can recognise that someone is high risk, which is fine, but they don’t necessarily know which pathway they should clearly go down.”*

One midwife was asked about her understanding of what occurred in the preterm birth surveillance clinic:

Interviewer: *“So, you refer your woman there. Do you know what actually happens behind the closed door?”*

Midwife HSI004: ***“****No, I do not. I think there is some transvaginal scanning that goes on. I think there is some mystical measurements of cervixes [laughter] and things like that. A lot of planning is for… discussing options. Yes. And possibly prescribing things. But yes, I think that is probably it. Assessment, timing and prescription.”*

Despite being broadly correct in her answer, she was still filled with uncertainty and doubt. This is highlighted through her use of the words ‘mystical’, possibly’ and ‘probably’. She went on to explain how not knowing what occurs in the clinic means she is unable to know when or why appropriate referrals should be made, and the effect this has on the woman’s care pathway.

Midwife HSI004: “*I do not really have any idea, I guess, of the prioritisation or the hierarchy of risk for all the preterm births, so I know that there are all these risks and I know that they all need to be seen, but I… when they go out to the receptionist or whenever I ask for them to be put onto the preterm birth clinic, but I do not really know if there are some that I should really try and expedite the speed of the...[appointment]… Because sometimes they are not available for many weeks depending on what is happening because the doctor might be away or something like that. I do not really know what the higher risk factors are and also, I do not really know what to do if the doctor is away, what else I could do to support the woman or the person to get the care that they need in the absence of that specialist input.”*

*

Obstetrician LSI004: *“So a prime example is in a trust elsewhere they had a woman that had had a LLETZ and they scanned her at 16 weeks and that was normal and so this staff grade discharged her and she came in at 23 weeks with ruptured membranes and was transferred…and I picked up this woman and could straightaway see that she has just been treated as a number not as an individual and they’ve not thought about why she may be at risk. So she’s had a scan, it was normal, but it was done at the wrong time, and there’s a reason why Saving Babies’ Lives says if you are intermediate have a scan, if you are only going to have one scan have it 18 to 22 weeks because we know that maximum pressure on the cervix is around that 20, 22 week mark, so if you scan them too early of course it’s going to be normal, there’s not enough pressure, and that is a prime example of they’ve just followed the guidelines - she needs one scan and if it’s normal she doesn’t need anything. And if you need to come back to the pathology of it, not just follow a guideline.”*

Midwife HSI004 highlights how a lack of knowledge of what occurs in the preterm birth clinic impedes her ability to undertake referrals confidently, as she does not know what an important timeframe is for arranging each woman’s appointment. Meanwhile consultant obstetrician LSI004 explains a case where a lack of pathology knowledge and subsequent timeframes for appointment affected a woman’s care.

These interview excerpts highlight that being solely aware of the risk factors for preterm birth is not enough to ensure appropriate risk assessing and referral. More knowledge on the pathology of preterm birth is required to determine if this corresponds to the woman requiring preterm birth surveillance care or another specialist service, and when appointments with this specialist care should be provided.

Context: Education on pathology

Education to junior doctors and midwives on the pathology of preterm birth so they could understand what is provided to women in preterm birth surveillance clinics and at what timeframes, was recognised as important by clinical leads (two consultant obstetricians and a lead midwife) at all three sites.

Consultant Obstetrician HIS002: *“But it was really interesting to me, again, just basic knowledge. If you explain what it is you are trying to avoid, rather than just randomly saying, ‘We are going to have a programme…’ Knowledge is power.”*

***

Lead midwife for antenatal clinic and day care YSI003*: “Or it could be that they [the midwives] are not confident enough to go, “Yes, you will definitely need to have a consultant appointment.” Yeah, they probably don’t know the pathway. Because obviously, we see these ladies a lot earlier than we see a lot of the other ladies. So in most cases we try and get them in 16 to 18 weeks for normal antenatal clinic, whereas obviously we want to get these ladies in for 12 weeks. So the fact that we are suddenly sending out appointments really quickly, that might be a bit of a worry if they haven’t been told. And I don’t know if the community midwives would know the difference timing-wise.”*

***

Consultant Obstetrician LSI004*: “I don’t think they truly understand what we do and who we do things to”*

However, while some knowledge would be beneficial, they were not expecting all staff members to be experts in preterm birth.

Obstetric registrar LSI003: *“I think they know that we’ll do some…scans. They know we offer some women sutures, some women are on progesterone. I think it’s just the very sort of nuance of exactly how regularly we do the scans, because I think…a better understanding that anyone has I think would be better, but I don’t think, if you’re not directly providing that service, you need to know the minute details of exactly who or what happened with regard to treatment. I’m not sure. If you’re not providing that treatment. But I think it’s really important that we all have a basic understanding of what happens. Because if you refer a patient to a service, you need to give them a heads up as to what to experience at the clinic, especially somewhere where you’re going to have an internal scan.”*

*

Consultant obstetrician LSI004: *“…it comes back to trying to educate them about the pathology of preterm birth and I think that’s all they need to know. I don’t think they desperately need to know every ins and outs of what we do in the clinic and when we treat someone and when we wouldn’t because that is nuance…”*

The clinical leads at each site had tried to educate midwives and junior doctors on the pathology of preterm birth risk factors to help them understand what is provided to women in preterm birth surveillance clinics and at what timeframes. However due to high staff turnover and fluctuating staffing levels, they now felt they were now back to square one.

Consultant Obstetrician LSI004 *“…likewise prematurity, they don’t understand the risk factors or why this woman may be more at risk than others. We did do some teaching, I’ve done annual teaching for the last couple of years with MAC [Maternity Assessment Clinic] about preterm labour symptoms almost, so talked about risk factors, so looking at twins, LLETZs, full dilatation, so making sure that they know who the people are to come in, to be seen in, because if someone rings up with a mucusy discharge, they might leave them at home, whereas if they’ve had a LLETZ procedure or previous full dilatation section they now know that actually that’s a woman to worry about. I think we’ve had good progress with that but that’s because we had dedicated MAC midwives as such that became experienced in that area, in the triage area, but because of staffing over the last year or so I think that’s got really worse again, so you are getting people that aren’t normally in triage triaging high-risk women again.”*

*

LMNS Maternity Transformation Midwife/Maternity Matron YSI001: “*I sent out the email, probably email with the guideline and then obviously the bits of what we were concentrating on and then, I haven’t done one for a long time but I do go along to the community meetings quite regularly. So I did a bit about it there. Probably need to do it again, to be honest, because we’ve had a few new midwives come in…* *we need to ensure that we’re making sure that they have some of the knowledge that we’ve already imparted. We can’t just do it once, can we? …we probably need to do an update with them, because we have had a few [new midwives] come out to the community recently.”*

Staffing levels are obviously a wider contextual issue that is currently affecting the whole of the NHS.

While it was clear that staff at sites do have a lack of knowledge, all sites had tried to navigate around this by double vetting the referral forms that midwives undertake. At two sites the consultant obstetricians triage the referrals to ensure lack of knowledge of what occurs in the clinic does not lead to inappropriate referrals, rather than ensuring all staff have that knowledge initially. At another the lead midwife triages the referrals.

Obstetric registrar LSI003: *“I don’t know how well their understanding of exactly what happens in the clinic is. In terms of who to refer, I think most have a decent grasp of that because it’s brought to light in the induction, brought to light in the guideline. I don’t know that they all know exactly what the cut off is. So they might get a few of them saying ‘oh this woman’s had a delivery at 36 weeks, do I need to refer that?’ But I think in terms of previous preterm deliveries, previous preterm SROM, that the more clear ones yes. I would like to think they know, like a uterine variant would need referral but maybe the more junior ones perhaps maybe not. But that’s partly why, it’s the more subtle things like that, it’s partly why I think a lot of the consultants are triaging the clinics now. So before they come to clinic, they’re triaging the notes and they’re trying to direct patients to the right clinic in the first place.”*

***

Lead midwife for antenatal clinic and day care YSI003*: “…when we brought in the whole triage thing, it did add more pressures to it because we actually have to log on and look at everyone’s referral form in quite a lot of detail and fill out one of our triage forms.”*

***

Consultant Obstetrician HSI005*: “…once they’re booked they [the midwives] actually fill the referral form and send it to a set Preterm Birth Clinic email. I vet it personally, and then ask our admin staff to book patients in.”*

***

Maternity care assistant HSI001: *“…the first point of contact is going to be the Consultant who is leading on this clinic, so she is going to get the referrals on the Preterm Birth Clinic email address and she’s going to vet that, so she’s going to have a look at who needs to come for the appointment and who is not eligible to have a Preterm appointment. And once she’s happy to see them, then she’s going to forward the referral to us, and that’s when we’re booking an appointment for the patient.”*

While educating junior obstetricians and midwives on the pathology of preterm birth takes time and effort for leaders, especially if this has not already been undertaken before they qualify, it should only need to be undertaken for each member of staff once. The double vetting of referrals takes time and effort that continues indefinitely. While it also prevents inappropriate referrals that have been made and are not required, it does not prevent referrals not being made even when they would be appropriate. When midwives are aware of double vetting, it also undermines them taking responsibility for knowing the pathology of preterm birth as they know they can rely on the safety net of an informed person checking their referral.

Outcome: Appropriate referral

Refined Programme Theory 2 after data collection: IF leaders have educated staff on the pathology of preterm birth (C), THEN appropriate referral to the preterm pathway is more likely (O), BECAUSE staff will be aware of what is provided to women in preterm birth surveillance clinics at different timeframes and why this is important (M)

Two observations illustrating this full Context-Mechanism-Outcome triad are below:

Midwife LSO005 in Observations 079-082: *Booked service user LWO055 who had a previous spontaneous preterm birth at 21 weeks and 5 days the previous year. Midwife LSO005 did not initially refer her appropriately to a preterm birth clinic, and referred her inappropriately to the rainbow clinic (O) whose referral criteria was for stillbirths after 24 weeks’ gestation. She explained after the appointment that she was not aware of what was provided by the specialist clinics, and she ‘felt a bit bad…I don’t even know what the rainbow clinic does’ (M). Midwife LSO005 explained that she did not feel confident in the pathology of preterm birth risk factors as ‘there’s so much to know… you end up feeling comfortable with just not knowing stuff’, being newly qualified she felt she hadn’t ‘been taught any of it, especially not at university’ (C).*

***

*Midwife LSO006 in Observations 083-084: Booked service user LWO056 whose obstetric history included a neonatal death at 29 weeks the previous year. The delivery was an emergency caesarean section due to small for gestational age and poor dopplers (i.e not a spontaneous preterm birth). Midwife LSO006 planned to refer her to both preterm birth clinic and the rainbow clinic, despite the fact her previous delivery was not a spontaneous preterm birth so did not meet the preterm birth clinic referral criteria. Midwife LSO006 seemed to be confused on her knowledge of what each specialist service provided. She seemed unaware of what was provided and the differentiation of each specialist service, explaining after the appointment how it was ‘a bit of a catch 22, she will be in rainbow and prem clinic’ and how ‘rainbow and prem can fight between themselves’ (M). After a few minutes she said LWO056 ‘is not strictly prem as she didn’t deliver spontaneously, she had an emergency caesarean. So I will refer to rainbow and then if they want to refer to prem they can do it’ (O). Midwife LSO006 explained that she did not learn about preterm birth risk factors or clinics during undergraduate training, but she has learnt a lot from working at this busy unit and actively asking questions (C), enabling her to ultimately make the correct referral.*

Midwife LSO006 made the correct referral in the end. After rethinking through the clinical case, she seemed to understand that referral to the rainbow clinic was correct due to the aetiology of what occurred. Interestingly she feels that her understanding was developed through her assertive searching and asking questions, rather than being taught either by leaders at her place of work, or at university.

**Programme Theory 3 – Practical difficulties**

Original IPT: IF staff have had multidisciplinary discussions to recognize what tasks are required by the preterm pathway and how they can easily integrate them (C), THEN implementation of the pathway is more likely to be successful (O), BECAUSE they will be able to understand what tasks are not being undertaken and therefore correct this to ensure that they are performing these required tasks (M)

Refined Programme Theory 3 after data collection: IF recognition was given to the practical difficulties midwives undergo to refer women (C), THEN easy and appropriate referral to the preterm pathway is more likely to be successful (O), BECAUSE support and solutions can then be found to easily incorporate the required tasks into their workload (M)

Programme Theory 1 ascertained how a lack of knowledge can lead to inappropriate referrals. Related to this, is that even if the knowledge is there, the system the midwives work in can be detrimental in itself to integrate tasks and make an appropriate referral. This again was highlighted in the second interview (undertaken for the IMPART study with participant HSI003 below), and led to the development of the subsidiary questions seen in Table 1 when collecting subsequent data.

Midwife HSI003: *“Even if it’s prompts, like something that would prompt us a bit more, because it’s so important when you think about it.”*

Context: Practical difficulties

Practical difficulties including computer systems, appointments being undertaken in the community and time were all highlighted as barriers to easy referral.

Midwife HSI003: *“…I feel like our system as well is just everywhere, where we’re handwriting the book, we don’t get to see scans because our thing doesn’t merge with the scans.”*

*Midwife HSI003 continues: “…the problem is some women just require different things. And where some of the boxes are compulsory, so if you don’t tick them you can’t move forwards, and I just feel like everybody keeps adding to it. So everything gets added but the time has not extended, and in the community you’re just by yourself. You can’t send the woman to the blood room next door, you can’t send her to the scan upstairs, so I think that’s where Community Midwives find it a little bit more difficult, as opposed to the Midwife sitting in the Antenatal Clinic.”*

*Midwife HSI003 continues: “I guess we’ve got our guidelines and stuff, but the problem with guidelines and our intranet is that if I’m using a computer that is not a [hospital trust] computer, which it’s not, [laughs] I can’t have that at a click of my finger. So I’m having to even say to women when I know, “Okay, I remember there must be something here,” I’ll be like “Can I get back to you a bit later to discuss further, because I’m not sure at present if this is one of the risk factors,” and I’ve done that.”*

***

Superintendent sonographer YSI002: *“I think it’s the templates people use. Because they’re going through a period of trying to go more electronic and I think there is so many different templates versions and then we have referrals from outside of our area, they’re not always on that template, so I think it’s just, if we had more of a standardised IT, then that would be helpful.”*

*

Midwife YSI003: *“Yeah, and it’s just like there’s lots of those sorts of day-to-day things that get in the way of even doing things like referrals. So even then, you’ve got that worry then that, oh I’m going to miss someone, that I haven’t got time to do those referrals, I haven’t got time to do that triaging. And then, yeah, it just adds on a little bit more as well to the pressure of it all.”*

*

Midwife in Observation 059: *They say these things [policy] but have they ever come and just spent a day observing us or seeing how it would actually work in practice.*

Mechanism: Support and solutions

Many of the practical issues relate to wider problems within the NHS, such as lack of funding and a lack of pragmatic and smooth electronic systems. While it is unfeasible for individual staff members to be able to completely change these, they tried to make the small improvements when they could.

Midwife YSO002 in Observation 070: *Had made her own small, laminated cards held together with a keyring for different risk factors that warranted referral, including one for preterm birth risk factors. She kept this in her bag and brought it out at booking appointments to double check when to refer a woman.*

The lack of wider, national support over the wider practical issues was raised as being particularly frustrating by one consultant obstetrician, and echoed by a midwife.

Consultant obstetrician HSI002*: “We have had an Ockenden review recently, I am sure everybody else has had it. Actually, what we wanted was, we want people to come and say, ‘You know what? You need investment in this. You need investment in that.’ It is not to portray a service where we have funding, because we don’t, for many things.”*

***

Midwife HSI003*: “I think we’re just so stretched on resources”*

Despite these wider contextual constraints, some units did manage to incorporate positive changes.

Midwife YSO004 and midwife YSO005 in Observation 077*: Explained how they used to have to know the preterm referral criteria themselves, but a few months ago their system updated, so now the risk factors are integrated into their ‘refer to obstetric team’ tab.*

Outcome: Appropriate referral more likely

Refined Programme Theory 3 after data collection: IF recognition was given to the practical difficulties midwives undergo to refer women (C), THEN easy and appropriate referral to the preterm pathway is more likely to be successful (O), BECAUSE support and solutions can then be found to easily incorporate the required tasks into their workload (M)

Three observations illustrating this full Context-Mechanism-Outcome triad are below:

Midwife HSO003 in Observation 064*: Explained how the preterm clinic obstetric lead sends through an updated preterm birth clinic referral criteria sheet regularly (C), so she keeps the updated preterm birth clinic referral list up on her computer screen (M) so when she is undertaking a booking appointment, she knows easily which women are appropriate to refer(O).*

***

Midwife HSO004 in Observation 065: *Explained how the preterm clinic obstetric lead sends through an updated preterm birth clinic referral criteria sheet and there are preterm guidelines on the intranet (C), so she keeps the updated preterm birth clinic referral list up on her computer screen before each booking appointment (M) so when she is undertaking a booking appointment, she knows easily which women are appropriate to refer (O).*

Despite midwife HSO004 having a solution described above, she did feel that this could still be improved.

Midwife HSO004 in Observation 065: *Felt that the referral process to the preterm birth clinic would feel more streamlined if it was integrated into the computer digital booking system, in the same way that eligibility for aspirin and small for gestation age risk factors are.*

# Explanatory area 2: The preterm birth surveillance clinic

Table S2.2 IPT and questions asked of the data for ‘the preterm birth surveillance clinic’

| **Explanatory area** | **Programme Theory** | **Nodes incorporated** | **Original IPT** | **Subsidiary questions** | **Refined Programme Theory** |
| --- | --- | --- | --- | --- | --- |
| The preterm birth surveillance clinic | Programme Theory 4: Concentrated knowledge and expertise | - Core group of staff - Maintain trust in each other’s work and expertise (3.2 relational integration) - Midwives - Who to contact for advice | IF staff are given space to develop respect and trust in each other’s work and expertise (C), THEN implementation of the preterm pathway is more productive (O), BECAUSE as a multidisciplinary team they are more likely to understand each other’s, and therefore work to each other’s, strengths (M) | 1. In your opinion, is there a benefit of having a core group of staff? 2. How do you think a core group of staff develop a specialist concentration of knowledge? 3. Could you explain if the core group of staff be multidisciplinary? | Refined Programme Theory 4 after data collection: IF there is a core group of multidisciplinary staff specialising in preterm birth surveillance (C), THEN successful implementation of the preterm pathway is more likely (O), BECAUSE they can develop concentrated knowledge and expertise (M) |
|  | Programme Theory 5: Transvaginal scanning skills | - TVUS - SBLCBv2 as a vehicle for change | Not developed prior to data collection. | 1. Could you explain the importance of undertaking your own TVUS (rather than sonography department)? 2. How do you think this affects how you care for these women? | Refined Programme Theory 5 after data collection: IF the staff specialising in preterm birth surveillance have transvaginal scanning skills (C), THEN women are more likely to get nuanced, individualised care(O), BECAUSE staff have the skills to understand and explain appropriate care planning (M) |
|  | Programme Theory 6: Work together with local network | - Work together with local network - Modify their work in response to monitoring (4.4 reconfiguration) | IF staff feel comfortable to question modifying the preterm pathway as a result of their appraisals with their colleagues and managers (C), THEN implementation is more likely to be successful (O), BECAUSE they are likely to redefine the pathway to be more suitable and realistic for their hospital unit (M) | 1. What do you think is the effect or working together with your local network? 2. Are there any help/resources that can be given by others in the local network? 3. How do you think this modifies how you implement the preterm pathway at your unit? | Refined Programme Theory 6 after data collection: IF sites work together with their local network (C), THEN there are likely to be less variations in care for women within the region (O), BECAUSE they will then have the resources to redefine the pathway appropriately at their local unit (M) |
|  | Programme Theory 7: Supported adequately | - Adequately supported by host organisation (3.4 contextual integration) - Finances | IF management ensure that the preterm pathway is adequately supported with adequate finances and resources (C), THEN the pathway is more likely to be implemented successfully (O), BECAUSE staff will not have to worry about these periphery issues (such as funding, managerial support, time etc) and therefore can focus on delivering the pathway, feeling motivated and encouraged by their managers (M) | 1. How does your unit feel adequately supported? 2. Could you explain to me how this makes you feel? I was thinking it means you stop worrying/makes them feel supported…? | Refined Programme Theory 7 after data collection: IF management ensure that the preterm pathway is supported with adequate finances and resources (C), THEN the pathway is more likely to be implemented successfully (O), BECAUSE staff will feel supported in delivering the clinical care that is required (M) |

**Programme Theory 4 - Concentrated knowledge and expertise**

Original IPT: IF staff are given space to develop respect and trust in each other’s work and expertise (C), THEN implementation of the preterm pathway is more productive (O), BECAUSE as a multidisciplinary team they are more likely to understand each other’s, and therefore work to each other’s, strengths (M)

Refined Programme Theory 4 after data collection: IF there is a core group of multidisciplinary staff specialising in preterm birth surveillance (C), THEN successful implementation of the preterm pathway is more likely (O), BECAUSE they can develop concentrated knowledge and expertise (M)

As data collection began it became clear that the original IPT was not quite right. It had established the importance of multidisciplinary team, but this sat better as a context rather than its original label as a mechanism. The preterm pathway was not more productive, or more likely to be successfully implemented, simply because the team was multidisciplinary. The mechanism that created that outcome was their concentrated knowledge and expertise, which was gained through having a multidisciplinary team with a varied skill set of knowledge and expertise. This led to the development of the subsidiary questions seen in Table 1 when collecting subsequent data.

Context – Core multidisciplinary group

Where there was a core multidisciplinary group of staff specialising in preterm birth surveillance, the team fully appreciated each other’s strengths, and trusted in each other’s work.

Maternity care assistant HSI001: *“I think it’s all about teamwork, isn’t it? So if we wouldn’t be respecting each other then the whole thing just wouldn’t work really nicely. Personally, I wouldn’t like to work with someone who thinks that just because I’m a Maternity Care Assistant I’m not on the same level as them, or just do my job and just leave them alone kind of thing, it isn’t going to work, it’s not giving a good atmosphere around the clinic. So, it’s really important that she thinks that we are a great help; she’s always saying thank you, she’s always appreciating what we’re doing for her. And it’s the same way around; we think that she’s a great doctor, she’s doing her best, and that makes us think the whole thing, even the clinic, me personally, I like this clinic, I’m looking forward to doing it…”*

***

Consultant obstetrician LSO003 in Observation 038: *Explained how he feels it helps now having specialist preterm midwives working in the team which they did not have before. He feels that they can focus on the midwifery elements which the preterm clinic did not focus on before. It also means the preterm midwives can continue to see women once they have been formally discharged from having cervical length scans.*

***

Preterm specialist midwife LSI001: *“No, I think they appreciate the midwifery input, so from their point of view, they’ve done this job for a long time in a successful clinic but felt like they needed some ownership in midwifery care for these women. They're complex, often emotional and psychological, issues. And in how midwifery is, there's a lot of clinic cover within the GP clinics. They appreciate our input and ability to support these women. There's a natural respect in that they see our role and appreciate it’s something they’ve wanted for a while. For their breadth of experience and knowledge and how dedicated they are, we really respect them. So the communication is very good between us.”*

She continued: *“we play to each other’s strengths…definitely.”*

She explained: *“you definitely have women [at risk of preterm birth] who are very worried [laughs], and that’s why a midwife is needed in that kind of speciality.”*

***

Preterm specialist midwife LSI002*: “I think it definitely helps running a… multidisciplinary clinic because [the obstetricians] are fantastic in what they do, but they are obviously not midwives and obstetricians tend to be more path focused so midwives can look more at the holistic picture. And [the obstetricians] knew that was what was missing from the clinic and that is why they wanted midwives to be there because they wanted more holistic care for the women who were in the clinic and that is what midwives are great at [laughter]”*

She continued: *“I think they [the obstetricians] do trust us to do our role, I guess. We were both community midwives before so they know that we can deliver antenatal care and I think they trust us to know the limits of our knowledge as well”*

She explained: *“[as specialist preterm midwives] we give a lot of emotional support, but then it is typical things like social services or housing or smoking cessation or BMI that they need referring for a Healthy Start programme or… all the things that a midwife does in the clinic every day, but because the preterm clinic is so specific, it is like…* i*t is a very specific part that we can take into account everything and a lot of preterm births, it is like modifiable risk factors, so giving more of the holistic care is going to improve outcomes.”*

***

Consultant obstetrician LSI004*: “..we just have that same ethos. And I don’t know, [laughs] we wind each up other and we’ll nag each other but ultimately, I think we just respect each other’s decision making which is how it works. And I think we both have the same work ethic. So when I look at teams that don’t function as well as ours, there’s often a worker and then someone that’s a bit slack [laughs]. …[We] will both stay late if we have to. We both care for the patients in the same way and I think we are both there to care for the women as opposed to some people that just rock up to work and want to go home and don’t want to do anything above and beyond. Whereas if a patient needs something we will do it. And that’s probably more fool us but that’s just probably our personalities and I don’t think you can change that in people necessarily, can you?”*

***

Consultant obstetricians LSO002 and LSO003 in Observation 011: *Asked specialist preterm midwife LSO001 how the women seemed during her preterm midwifery appointment which she had just had, before calling her in for her preterm cervical length check. This demonstrates how they respect and trust the specialist preterm midwife’s assessment to warrant asking.*

***

Obstetric registrar LSI003: *“I think the women feel, they’ve often been through a loss or traumatic experiences of losing babies early and to have that regular meeting with the same person and that continuity of care with a preterm midwife, the continuity of care is proven to reduce preterm outcomes, isn’t it? And it gives them that reassurance.”*

She continued*: “Well it’s just in terms of their [the specialist preterm midwives] understanding of our guidelines and education and who we care for, so their communications with the patients are more sensible, because they understand exactly what’s happening in the clinic [compared to midwives who are not specialist in preterm birth]. It’s again continuity of care and their caseload are all higher risk women. So it’s that continuity of care massively for the patients and ensuring all of our investigations, like cultures and swabs and things are all followed up…the smaller things, like ensuring the younger women are screened and stuff like that. I know it should happen in a general clinic but it’s just all stuff that can easily get missed if it’s not one of the top things that you’re looking to.”*

Some sites did not have a multidisciplinary specialist preterm birth team but appreciated the benefits it could bring.

Consultant obstetrician HSI002: *“…if you think about it, cervical scanning, there is no reason why a midwife couldn’t do that… it is very much doctor-led, and that can only go on for so long”*

***

Midwife HSI004*: “…a really helpful thing would be actually is if there were a contact midwife who is… it probably would not be a full-time job obviously, but I guess a link midwife, a preterm link midwife, who would be a midwifery contact for… about preterm birth.”*

***

Consultant obstetrician HSI005: *“So that is also the next step, whether we have the Specialist Midwife who I train to do cervical lengths.”*

*She continued: “I think multidisciplinary in today’s world is the key to everything good about patient care.”*

*She explained: “I think it is having someone else [a specialist preterm midwife] who is part of the team, where it will help her grow as I have grown in this clinic. Plus also for the patients it is very important because it becomes a very more personal care. …I think it’s the teamwork which will help, and also it will help having a Specialist Midwife because I feel they’re the main gatekeepers for the right people to come into the clinic as well.”*

However, where there is not a large team, and only one preterm birth specialist can undertake necessary requirements of the pathway such as transvaginal scans or cerclages, complications can arise.

Consultant obstetrician HSI002: *“And of course, she [the consultant who runs the preterm birth clinic] is one person, right? So, if she gets sick, God forbid, or she is away, the service stops.”*

***

Consultant obstetrician HSI005*: “However, currently it’s a one-woman show and that is always a problem. I pretty much run the clinic with just the help of admin support, an [maternity support worker] who actually just helps me with the blood pressure, but the rest of the job is all done by me. …it’s not enough. …So that is also the next step, whether we have the Specialist Midwife who I train to do cervical lengths. Because currently where I am, even I have to choose my annual leave around the Preterm [laughs] Birth Clinic, and what worries me is I’m a one-woman show where also… it’s become quite niche”*

Meanwhile at larger sites which could afford a larger team, they appreciate that they did not have these issues.

Consultant obstetrician LSO003 in Observation 038: *Thought it helped having two specialist preterm birth consultants as it meant they could provide cover 52 weeks of the year.*

Mechanism – Concentrated knowledge

By having a core group of multidisciplinary staff specialising in preterm birth surveillance, it meant they could develop concentrated knowledge and expertise in the field of preterm birth. This allowed them to hone skills in nuanced decision making, while also meaning they became experts in undertaking practical skills such as transvaginal cervical length scans and cerclages.

Obstetric registrar LSI003: *“having someone, people…who are extremely knowledgeable in their subjects and regularly doing these kinds of surgeries, that experience I think is invaluable. And I think if these women are in the mix of a general clinic, with a consultant who doesn’t have a special interest in it, then there are certain subtleties and certain differences that won’t get picked up on”*

***

Consultant obstetrician LSI004: *“…if someone showed me a scan and it said a cervical at 25, or 24, what does that mean? Well it means different in everyone, doesn’t it? If at 24 we’ve massive funnelling and I can see the cervix is under real strain then I’m likely to think oh she’s 20 weeks, she’s delivered at 24 weeks before, she needs a stitch, but if it’s 24 and strong and she’s had a couple of LLETZ procedures, well actually that’s fine, that might be all she needs. She might not need anything, that’s normal. I know the national guidance talk about 25 as a cut-off, but it’s 25 to consider treatment, it doesn’t mean you have to do something. But that nuanced care only comes with experience and this is what we get from elsewhere when we get referrals in, it’s ‘oh well the cervix is 22, so it says we need to do something’. Well not necessarily… but that definitely comes with experience…”*

Outcome – Implementation

Refined Programme Theory 4 after data collection: IF there is a core group of multidisciplinary staff specialising in preterm birth surveillance (C), THEN successful implementation of the preterm pathway is more likely (O), BECAUSE they can develop concentrated knowledge and expertise (M)

All of the sites where data was collected were able to implement a preterm pathway that fitted with the requirements of Saving Babies Lives Care Bundle Version 2. Having a core group of staff with concentrated knowledge and expertise seemed to lead to more sustainable implementation of the preterm pathway through in numerous different ways. Firstly, it helped to reduce variation in care for women access care within that hospital.

Consultant obstetrician HSI002: “[having a set group of people providing preterm birth surveillance care] *I think it would reduce variation in care”*

***

Lead midwife ANC clinic and day care YSI003: *“I think it’s just that they literally will become very skilled in that one area and especially if they’ve got an interest. I think that really shows if they’ve got a particular interest in something…And also it helps us, we know that on those days these women will need that little bit more input. It will just help the women, I think, they’ll be getting the care that they should be getting.”*

***

Obstetric registrar LSI003*: “In terms of planning appropriate follow-up and the optimum management plans will get missed. Perhaps certain things like making sure that the urine cultures are sent off, that the test…are done for infections, stuff, smaller things like that, if you’re in a general clinic are more likely to get missed, I think.”*

***

Obstetric registrar LSI003*: “we get told by so many patients, ‘we’ve been told different things by different doctors and different midwives, and different things by different nurses’. And having a clear, consistent message to the patient I think is really helpful for them. Rather than confusing things.”*

***

Consultant obstetrician LSI004*: “..he taught me all my scanning and the theory of how we practice our prematurity risk factors and causes and so we both practice the same way and so if one of us is away we know we can leave… I think it works good for us as well because we trust each other, do you know what I mean, so if [he] is away he knows I’m going to look after the women the way that he would want them to be looked after. And likewise if I’m away, I know that he would. So I think it works both for the team and for the patient.”*

***

LMNS Maternity Transformation Midwife/Maternity Matron YSI001: *“what we did with our pathway was made it very prescriptive that those women would be seen by just two of our consultants. … And the reason why we went with just two consultants is then because we felt that we were more likely to ensure that the women who were booked on to that pathway followed the pathway robustly, whether we had just booked them into any of our consultants. It wouldn’t have been the same. I feel that we felt that we needed to be able to have a little bit of control over it, but not having a clinic but having just two clinicians that saw all of those women, if that makes sense.”*

She continued: *“…it’s a bit more robust than when it’s just ad hoc people coming in, because they might not be familiar with exactly what the guidance is. They might not be familiar with the pathway that we’re trying to follow and the reasons why”*

***

Lead midwife ANC clinic and day care YSI003*: “But if they go to everyone, everyone’s maybe doing something slightly different, which is not the ideal – we want everyone to be getting that same experience. So I think, just having one or two, they’ll be doing exactly the same things for the women.”*

Secondly, having a specialist team meant it was easier for the specialist team to impart their knowledge to junior members of staff so that juniors could learn how to undertake transvaginal scans and cerclages. This would help to ensure successful implementation and sustainability at the sites.

Consultant obstetrician HSI002: *“…those people who could then teach. At the moment what is happening, and I am sure it is the same everywhere, is that a variety of different random consultants are teaching. So, I will do a stitch with somebody, and I will go, ‘Who taught you? No, no, that’s not…’ I just can’t be... So, I think it is better.”*

***

Obstetric registrar LSO004 in observation 025: *She was struggling to get a clear cervical length measurement through the transvaginal scan. She asked if one of the specialist preterm birth consultants could come and help, without seeming embarrassed at seeking help. When one of the consultants came in, they did not sigh or seem unwilling. They allowed obstetric registrar LSO004 to still lead the appointment but were there for support when required.*

***

Obstetric registrar LSI003: *“Them [the specialist preterm consultants] trusting my scanning and everything has built up as they trained me. So they used to sit and help me with every scan, which is understandable because I was learning, but now it’s more of an indirect supervision and I guess because I’ve learned through them over the last year, they’re happy that I’m making appropriate plans because I’m learning from how they do it.”*

An example of this is highlighted in the context-mechanism-outcome triad below.

Consultant obstetrician HSI002: *“…you just become good when you are doing something all the time. I think then that would allow somebody to develop their career. I think it’s the idea that people become consultants and that’s it, it seems mad to me. You need to be better, continuously.”*

She continued: *“Another concern I had, and I know Andy Shennan talks about it, is that cervical suture is done by a variety of different people. We have people who don’t do any obstetrics, who just do a course, and they suddenly go and put a stitch in somebody. Or we have an ST3 somehow is doing a cervical suture on a woman. (C) Then when it fails (O), then you don’t actually know. You think, well, was it because the ST3 was doing something that maybe…? You don’t know, right?...* *I was suggesting that the preterm consultant actually consolidates the sutures, and does most of them herself, so she can then get more experience. (M) She can then teach.”*

Thirdly and finally, having a specialist team meant it was easier for other members of staff know who to contact for specialist preterm advice (for example midwives who have a question regarding referring a woman to the preterm birth clinic). This would make successful implementation of the preterm clinic more likely.

Midwife HSI004: *“For example, we have a preterm birth clinic and we also have a prebirth specialist consultant and I feel like she is always the person… she runs the preterm birth clinic, so I feel like I can always go to her with questions about preterm birth issues, if you like, queries. So, that is tremendously helpful.”*

***

Consultant obstetrician LSI004*: “if a midwife thinks ooh I think she should have probably been seen at prem clinic then they quite frequently will email and just ask us to have a quick look at her notes”*

***

Midwife HSO003 in Observation 064: *Feels that it is good to have a named preterm birth specialist as she then knows who contact with questions.*

## **Programme Theory 5 – Transvaginal scanning skills**

Programme Theory 4 highlighted the importance a core group of multidisciplinary staff specialising in preterm birth surveillance who can develop concentrated knowledge and expertise. However, during data collection it became clear that one area that required more in-depth consideration was that of transvaginal scanning. Sites could have a named consultant for preterm birth surveillance care. However, if their transvaginal cervical length scans were undertaken by the sonography department instead of by them, then this caused different issues. The preterm pathway could still be successfully implemented (as per Programme Theory 4), but that nuanced and individualised care was missing. This led to the development of Programme Theory 5 and the subsidiary questions in Table 1.

Refined Programme Theory 5 after data collection: IF the staff specialising in preterm birth surveillance have transvaginal scanning skills (C), THEN women are more likely to get nuanced, individualised care(O), BECAUSE staff have the skills to understand and explain appropriate care planning (M)

Context – Transvaginal scanning skills

Staff at all the three sites agreed on the importance of transvaginal cervical length scanning techniques.

Interviewer: *How important do you think it is that the Obstetrician making the plan is the same Obstetrician who’s undertaken that scan, rather than it being done by a Sonographer or done by someone else? Do you think that’s important when it comes to preterm birth care?*

Consultant obstetrician HSI005: *I certainly think it’s not only important, it should be mandatory. [Laughs]*

However, barriers were identified as to why many clinicians are not trained in transvaginal cervical length scanning.

Consultant obstetrician HSI005: *“…there are two reasons; one is historical and one is training. Historically, scanning was not viewed as a very sexy part of being a Gynaecologist, until the late 1990s and 2000s. So if you look at it for a Gynaecologist, knowing what a fibroid looks like and how to cut it is very important. But if you don’t do that scan yourself, it’s like asking a Cardiologist to use your stethoscope by a General Medical Physician to listen to that murmur and then prescribe the medication; do you see what I mean? …So historically, that training hasn’t been because everything has been quite surgical. Until it was introduced, the Royal College didn’t have it as a curriculum induction until mid-2000s… “If you can’t have a Cardiologist without a stethoscope, why would you expect to be a Gynaecologist without knowing how to know your diagnostic pathology via a scan in a patient?” …But obviously listening to a heart is very different from learning how to scan because it takes years to learn how to scan plus how to be. But cervical length certainly because it is not part of our curriculum.”*

***

*Preterm midwife LSI001: “I think that is probably what I've noticed really, being on the local LMS calls about what our local trusts are doing. They don’t seem to have the allocated time or staffing, and they have a lack of confidence I think in managing these women. I don’t understand how they get trained to be able to do that really. That is one of the questions I've often asked, why can't whoever does the cervical scan, or if consultants do the cervical scan, do the sutures? But how is a smaller trust expected to be able to deliver that if they’ve not got the training?”*

***

Consultant obstetrician LSI004*: “And the thing is, things like this, you’ve got to be doing all the time to get that nuanced care. There is no way that I or now [the current preterm registrar] could have got any of that experience without taking time out of general training and focusing on that sort of area of our care. You couldn’t just pick it up in a general clinic, there’s not enough time, there’s not enough time in your job because you are obviously covering on-call work and things like that. So these sort of specialist roles but from a doctor point of view, I think they need time out of their training to get that experience because you’ve got to be doing it all the time.”*

However, many thought that while learning transvaginal cervical length scanning skills took time, it was not a particularly difficult skill to learn.

Consultant obstetrician HSI005: *“It is tricky to learn the full scope of gynaecological scanning, and I’ve done it for coming up to four years [laughs] so I know to keep up that high standard of Level 3 scanning it takes a lot of effort. But trans-cervical scanning, pretty much if I see 12 patients and if a trainee comes and scans five of those patients and keeps up with that for the next three months, it is a skill learnt.”*

***

*Consultant obstetrician LSI004: “And if you are doing it all the time, cervical scanning is quite easy.”*

A sonographer did not mind who undertook the scans, as long as they had the correct qualifications.

Superintendent sonographer YSI002: *“Well as long as they have the same level of qualification. For the midwives, as long as they have the same level of postgraduate qualification as the sonographers, then I would fully support it. I mean, as a consultant that they do their [training] through the RCOG [Royal College of Obstetricians and Gynaecologists], they’ve got their sign off that they would have had as registrars and…consultant level, then it’s their responsibility to maintain those levels of competency. But in terms of, I’m not supportive of anybody picking up a probe and thinking that they can scan.”*

She continued: *“As long as they’ve got that postgraduate qualification. Because as I say, it’s not just the recognising of the [cervix], …it’s learning how to drive the machine. It’s knowing the physics involved and how to improve an image. So we don’t have any scanning midwives at present but I am quite open to the fact that I’m happy to train midwives, but it would need to be done through the same university as our sonographers go through.”*

Staff working in a unit where a dedicated preterm birth surveillance team did not undertake ultrasound scans recognised the benefits it could bring.

Superintendent sonographer YSI002: *“I think ideally one-stop is probably better for the patient. But the way that we’re set up…We would not have that …capacity… If we’re supporting Saving Babies’ Lives, it’s very difficult to then mix the clinics. If we were giving 45 minutes to an anomaly and a cervical length, in the middle of an antenatal clinic, one we don’t have that option and two we would be displacing two growth scans so then they wouldn’t be having a one-stop service.”*

*

Obstetric Senior House Officer YSI004: “*Yes, I think it would be better. Not just in preterm…but in general I think it would be better if registrars were able to do ultrasounds and not wait for the morning scans that we usually do. …So I think it would be better if registrars were able to do scans.”*

However, despite recognising this, implementing a dedicated preterm birth surveillance clinic was difficult for smaller sites. The size of their unit would affect if they could assign a clinician to be dedicated to preterm birth, and to ensure they developed the required skill set such as transvaginal scanning.

LMNS Maternity Transformation Midwife/Maternity Matron LSI001: *“I think it was probably the consideration of the document really, because everywhere in Saving Babies’ Lives, it was a prem birth clinic and it took a long time for us to get assurance back from national, regional people that it actually didn’t have to be a clinic, if that made sense. It could be a pathway. So yes, that in itself I think it didn’t really, I probably think that’s true with a lot of documents that come out, that they don’t pay credence to smaller sizes or maybe different areas, rurality and urban. They’re different, aren’t they, they are different things and setting something down in stone that you have to do, that might work in central London for 5000 women doesn’t work in rural Somerset for 1500 women. So I think that’s the difference. I think a lot of obviously, of course it would come from more central places, wouldn’t it? But sometimes it doesn't appreciate that other places have got their own problems that aren’t around being big and central, in an urban area really. So yes.”*

As staffing issues affect the whole of the NHS, sites could end up training clinicians how to scan and then they leave.

Midwife in observation O82: *‘You teach people and then they leave’.*

Mechanism – understand and explain appropriate care planning

Despite the barriers to learning how to undertake transvaginal scanning, it was felt that this skill enabled clinicians to understand and explain appropriate care planning.

Consultant obstetrician HSI005: “*I think the reason it should be mandatory [that specialist preterm clinicians know how to scan rather than use the sonography department] is as I’m growing in the clinic as well, I’m learning a lot of things. Because I think it’s very important to know if you’re the Lead Consultant of your patient, what their cervix looks like to make a decision on whether she needs a stitch or she doesn’t. Scans per se have interpersonal variations. And if you are reliant on somebody else’s measurements or scan or anything else, then you are…because it’s not very difficult to learn doing a cervical length.”*

She continued: *“I think from just overall being in charge of patient care. …the fact that the Obstetrician who makes the plan shouldn’t be dependent on the Sonographer doing the cervical length.”*

***

*Consultant obstetrician LSI004: “So, like my clinical director will frequently say, “Oh well do you not think the sonographers can just do the scans and you can just do the clinics?” and I think, number one, it wouldn’t help us because we actually do part of our chat whilst we are scanning, as you saw. It wouldn’t speed us up someone else doing the scan but also, again, if someone showed me a scan and it said a cervical at 25, or 24, what does that mean? Well it means different in everyone, doesn’t it? If at 24 we’ve massive funnelling and I can see the cervix is under real strain then I’m likely to think oh she’s 20 weeks, she’s delivered at 24 weeks before, she needs a stitch, but if it’s 24 and strong and she’s had a couple of LLETZ procedures, well actually that’s fine, that might be all she needs. She might not need anything, that’s normal. I know the national guidance talk about 25 as a cut-off, but it’s 25 to consider treatment, it doesn’t mean you have to do something. But that nuanced care only comes with experience and this is what we get from elsewhere when we get referrals in, it’s ‘oh well the cervix is 22, so it says we need to do something’. Well not necessarily. I’ve got women walking round with a cervix of 17mm that I’ve not done anything to but it looks nice and strong and tight, so yes she’s probably still going to have a preterm birth but it’s probably going to be 34 weeks. Putting a stitch in is probably not going to gain anymore. What I wanted to make sure is we are not going to have a really preterm birth, so it might be I need to increase her monitoring or maybe think about progesterone or a stitch but if it’s strong then actually she’s not got a problem at the moment.”*

***

Consultant obstetrician HSI005: *“And I think it’s the Obstetrician who actually makes the plan for whether this patient is for progesterone or suture, and also it’s a personal kind of preference as well because my emergency cerclages, I do them under ultrasound guidance. So I would always scan them before putting the stitch because one, apart from seeing what I see, I have a mental picture in my head how far my needle has to go so as to not rupture. So for me, it’s very important and if you’re the person making that decision for that emergency cerclage to be, I think it’s an important [laughs] skill as well.”*

Outcome – Nuanced, individualised care

Refined Programme Theory 5 after data collection: IF the staff specialising in preterm birth surveillance have transvaginal scanning skills (C), THEN women are more likely to get nuanced, individualised care(O), BECAUSE staff have the skills to understand and explain appropriate care planning (M)

This was highlighted in three context-mechanism-outcome triads below.

Consultant obstetrician LSO003 in observation 032: *Undertook a transvaginal cervical length on a woman (C). Her cervical length measurement was 20mm, however he feels it is V shaped not U shaped funnel, that does not slide over each side under a pressure effect, (M) and therefore explained that ‘I don’t feel we are at a critical level but you could do with some support. Its creaking but hasn’t given way completely yet’. He was therefore happy recommending progesterone and surveillance, rather than a cervical cerclage (O).*

*

Obstetric registrar LSI003: *“Yes. Yes, I think to actually understand the scan and the images in real time, rather than just from a report that someone else has done (C), and it allows you to counsel the patients better (O). The patients better understand as well, rather than, you can point at the screen instead of explaining it from a written report on the table. (M)”*

***

Consultant obstetrician LSI004: *“But the biggest thing is I’ll say, “Can you send me the image?” because to me a scan report of 24mm means nothing to me, I want to see the picture and therefore that’s why I think it’s really important for me to be doing the scan. And if someone has got that, the first thing I’ll do is repeat the scan myself. If they can’t send me an image, I’ll say, “Okay, send it to me and I’ll want to do the scan myself,” (C) because ultrasound, ultimately, is a dynamic process, isn’t it? It’s still images of rubbish really. You need to see exactly what’s happening. (M) And so unless it’s very clear on the image what I’m seeing, I will scan them myself. So I don’t think it saves us much time if a sonographer was going to do the scans. And also I think the women, we talk them through the scans, we show them what we are seeing, they get that reassurance from the scan as well because they start to understand what we are seeing (O) and I’m not certain the sonographers would do that as much. They are very much technicians, aren’t they, they don’t talk that much to the patient and so they’d still need to come through, I’d still need to get the picture up of the cervix and show them…so I may as well just do the scan myself. As you’ve seen, there’s some mornings where we will do 20 odd women. Well the sonographer is not going to do that, are they, and so actually what benefit are they going to get?*

An oppositional triad was also identified below.

Obstetric registrar YSO001 in observation 001: *Saw service user YWO001 in the antenatal clinic, who had just had a cervical length scan undertaken in the sonography department. Obstetric registrar YSO001 read the sonography scan report which reported a cervical length of 30mm (C). While checking the hospital preterm birth standard operating procedure guidance (which states ‘if cervical length is >25mm and no other risk factor continue as low risk care’) (M), obstetric registrar YSO001 explained that her measurement is over 25mm, and she therefore does not need further cervical length scans (O).*

One registrar felt that care was less individualised when standard operating procedure guidance is followed in this black and white manner, as illustrated in the above observation. If one does not undertake the scan themselves, they cannot give nuanced and individualised care.

Obstetric registrar LSI003: *“I would say the care is much less individualised when I’ve worked in places where it’s just, there’s no preterm clinic itself, there’s just a general antenatal clinic. This is the structure with scans, and this, this, this and that’s it. And 25 is the cut off exactly. Not, you’re looking at scan reports instead of the pictures, which are very different.”*

**Programme Theory 6 – Work together with local network**

Original IPT: IF staff feel comfortable to question modifying the preterm pathway as a result of their appraisals with their colleagues and managers (C), THEN implementation is more likely to be successful (O), BECAUSE they are likely to redefine the pathway to be more suitable and realistic for their hospital unit (M)

Refined theory Programme Theory 6 after data collection: IF sites work together with their local network (C), THEN there are likely to be less variations in care for women within the region (O), BECAUSE they will then have the resources to redefine the pathway appropriately at their local unit (M)

An early interview with a woman highlighted that she felt it was not just having a named specialist preterm clinician, but also down to learning from other units that perhaps have more knowledge and experience, to reduce variations in care for women like her within the region.

Service user LWI005: *“That everyone would have that specialist knowledge of that specialist consultant in their hospital and I suppose each member of staff would be able to learn from maybe the different patients because I suppose [the smaller local unit] are never going to get better with preterm if they do not experience it or have a specialist person. They might have a specialist person, but I just have never…I just feel like all… could what happened to [my baby] have been picked up earlier …if they would have had the awareness? But then it might not have been, but I just think if they had… they [the smaller local unit] will never experience someone like me …. because I have then just gone straight to [the larger teaching unit].”*

As LWI005 highlighted the importance of working together with your local network, this led to the development of the subsidiary questions seen in Table 1 when collecting subsequent data.

An early interview with a consultant obstetrician demonstrated that staff at sites were aware of what those in their local network were doing, even if they had not formally reached out to each other yet.

Consultant Obstetrician HSI002: *“So, when I came here, we were clearly the outlier in London, because we didn’t have foetal fibronectin at all. Even though we did look after 6,000 women, we only have a 9-bed antenatal ward… But because we were a level 3 natal unit, it always felt quite uncomfortable that we didn’t have this fibronectin, but everybody else around us did.”*

Context – Local network

Working with your local network was highlighted as important at three sites. However, some things helped and hindered sites working with their local network. This included the personalities of those asking for assistance, with some perhaps feeling unconfident asking for support.

Consultant obstetrician HSI002: *““I don’t know if this was egos, I don’t know what it is, but people are so obsessed by not picking up the phone. I have never understood that. I do maternal medicine. Even before these maternal medicine networks were established, we had each other’s phone numbers. You would pick up the phone. You worried for the patient, right?”*

She continued: *“…one is ego, and I think the other one, actually, is people are just scared, because they go, ‘Oh, but this professor in this ivory tower, I don’t know them. I’m a junior consultant.’ I think, we’re all just people doing exactly the same thing. I was always told that I was a junior of my professors. But actually, when you become a professor, it means you know very little about the world, but you know a lot about one tiny thing. [laughing]”*

Meanwhile, some felt confident in seeking support.

Consultant obstetrician HSI005: *“…what also was a big help for me was seeking help from UCLH…But through that I got in touch with Anna David… And Anna David actually helped me quite a lot because for her, it was helping me set up the Homerton to a set standard, and with me also she said there are few units which asked for help…”*

***

LMNS Maternity Transformation Midwife/Maternity Matron YSI001: *“So probably the only one is our neighbouring [hospital]…and they’re obviously bigger than us so they do have more of a clinic, rather than just a pathway. So I spoke to them about how they were going to be setting up their clinic, if they were setting up a clinic or whether they were going along a pathway. So I spoke to them over there.”*

The personalities of those at the larger, tertiary, teaching units, who were asked for help were also important. If they responded warmly, it was easy to develop a connection.

Consultant obstetrician HSI005: “*But most importantly I think it’s the personality, and the team were very keen to help. So she was very open with sending me all the [laughs] guidelines and even how to keep an audit, and I still actually once in four months catch up with her, so I would go to a Wednesday morning clinic and sit with her, and I’ve actually learnt a lot doing that so I must have had around three sessions still now which I do over a period of four months. I think it was the fact that they were keen to help.”*

Some felt the onus was on those at the larger units with more experience to make more effort to share their expertise.

Consultant obstetrician HSI002: *“And I think, for example, if a preterm [expert]… said, ‘Do you know what? We are going to have training, an hour talk on preterm labour. It is going to be done twice a year.’ In the evenings I think would work best, because most people can go to them, if they are only an hour. It is very difficult during the day, I have to be honest. But if they are recorded, then people can tap in. You just need one person to tap in, and they go, ‘I have heard this,’ and then start a conversation.”*

Practical issues also helped and hindered working with their local network. This included simple scheduling such as the day of the week that the clinic was held.

Consultant obstetrician HSI002: *“I always thought, at least if your preterm clinic was on the same day as somebody else’s preterm clinic in London, then you could just pick up the phone and say, ‘I have got this woman, I don’t know what to do. What would you do?’ That would make sense… So, then I said to them, ‘now everybody works on a Wednesday’.”*

Some highlighted the importance of maintaining positive relations with the smaller local units when caring for women. This continued when they were undertaking shared care with a local site, and when women transferred all their care.

Service user LWI001: *“I think they once mentioned something about discharging me back to Calderdale, not doing it then but just saying it, and I think I literally pooed myself. I was like, ‘Oh no!’ and I suppose I am dreading that day because that could happen.”*

*

Consultant obstetrician LSO003 in observation 012: *Explained how he encouraged the women to ‘kiss and makeup’ with their local units, even if they previously had a poor experience there and do not provide preterm surveillance care, because ‘ultimately that is where the ambulance is going to take them’.*

*

Consultant obstetrician LSO003 in observation 016 and 017: *“…nobody wants you to have a bad experience, the local team are also on your side. And if there is something they don’t understand or is too complicated then…you know how to come back and get in touch.” He then discussed putting their [the tertiary units] contact numbers, the operation notes from the cerclage, and the instructions for removal at the local unit at the front of the woman’s notes so the local unit can easily see them. The woman seemed happy with this plan saying ‘I can’t thank you enough’.*

*He explained that this way of working allowed the local unit to develop and reach out for help if they need it, which of course they will then provide.*

This positive relationship with local smaller sites was maintained to the women, even in cases where potential mishandling had occurred.

Service user LWO044 in observations 041 and 042: Service user LWO044 *attended her local unit at around 15 weeks’ pregnant, and they told her that her cervix was already short. Service user LWO044 came to the larger tertiary unit crying at 16 weeks’. Here she was scanned, and her cervix was found to be 23mm in length. Consultant obstetrician LSO003 said: “it’s a bit longer than what they said at [local unit], there’s no crisis here - it’s a bit shorter than we’d like but it’s not giving way just yet”. Service user LWO044 explained that the local unit said that if she didn’t immediately put a cerclage in, then they would have to admit her to hospital. Consultant obstetrician LSO003 said “I don’t think we need to admit you” and that they could either put a cerclage in now, or watch and wait.*

While the final outcome (what gestation she eventually delivered) of this case is unknown, a 23mm length cervix at 16 weeks’ gestation is not an acute emergency that requires hospital admission. Clinicians self-assured in delivering preterm birth surveillance care (such as consultant obstetrician LSO003) are confident managing this appropriately.

Mechanism – Resources to redefine their pathway

Once sites were in touch with others in their local network, there were many resources that their local network could provide. These resources included larger more experienced sites offering their guidelines, introducing them to others/networking and helping them with shadowing/training to develop confidence in clinicians offering preterm surveillance care in their local network.

Consultant obstetrician HSI005: *“So, a lot of my help came from UCLH [University College London Hospital] because I had access to their guidelines, I had access to how they ran their clinic, and knowing that they are the model people for Saving Babies’ Lives which we model ourselves into, a lot of my development of the clinic was done in the format of how the UCLH clinic is run”*

HSI005 continued: *“And as part of that [contacting UCLH] I got a lot of connections about the Preterm networks and all that which obviously I wouldn’t have known otherwise, and I didn’t know about [before]”*

HSI005 continued: *“**So she was very open with sending me all the [laughs] guidelines and even how to keep an audit, and I still actually once in four months catch up with her, so I would go to a Wednesday morning clinic and sit with her, and I’ve actually learnt a lot doing that so I must have had around three sessions still now which I do over a period of four months.”*

Resources also included direct help on specific clinical cases.

Consultant obstetrician HSI005: *“I was left with the situation where I had a fetal fibronectin of 166 and with a long cervix, and didn’t know what to do. So we did a bit of calling around but then settled on the fact that “Just start on the progesterone and I’ll see you in a week.” So I’ve just seen her on Tuesday but again, on one angulation if you actually look it feels like it’s funnelling with 15mm, so I don’t know whether the cervix is strange or she’s someone who’s got thyroid cancer in the past which took much, whether there’s something in the anatomy I genuinely don’t know. But the decision came out to me, just keep her on progesterone and scan her weekly, which I’m doing now…And then I think [colleague] called around a bit as well, I think she tried to get in touch with a couple of your Clinical Fellows and they said “Well, if the fetal fibronectin is high and the cervical length is normal then the chances are low,” so that’s how we manage. [Laughs]”*

***

Lead midwife ANC clinic and day care YSI003: *“No, I mean we’ve got contacts in the two local hospitals that are slightly bigger than us. So we can always contact them, and just go, “What about this?” And then obviously, if they are really high risk, we tend to refer up to Bristol. And that’s quite easy and we do that on a regular basis. And there’s quite a good relationship there, we can phone them up and stuff. So yeah, I think it works okay.”*

Others highlighted what sites were missing from not having these resources, despite perhaps already being in touch with their local network.

Specialist preterm midwife LSI001: *“I think that is probably what I've noticed really, being on the local LMS [local maternity system] calls about what our local trusts are doing. They don’t seem to have the allocated time or staffing, and they have a lack of confidence I think in managing these women. I don’t understand how they get trained to be able to do that really. That is one of the questions I've often asked, why can't whoever does the cervical scan, or if consultants do the cervical scan, do the sutures? But how is a smaller trust expected to be able to deliver that if they’ve not got the training?”*

Even if they did not need help on specific cases, ensuring smaller individual sites felt supported and not isolated was highlighted as important.

Consultant obstetrician HSI002: *“But it was quite difficult to get people to understand that this person needs… I think when women have lost babies, it is such a traumatic thing. It seems outrageous to me that they would have some kind of follow-up here, but then nobody would write to Andy [Shennan] or Anna David and say, ‘I’ve got this, what else would you do?’ Sometimes there is nothing else you would do. But it is very difficult when it is just you working in silo, and you haven’t got a team of people to support you.”*

***

Consultant obstetrician LSI004: *“These strange cases, sometimes we’ll [LSI004 and the other preterm consultant] look at each other and go, “Am I being stupid?” and they’ll say, “Well I would have stitched that,” or, “I would have done that,” and it’s having… And I think that’s really hard if you work in a smaller unit, you’ve maybe not got that phone-a-friend person. And [the other preterm consultant] and I probably turn out to be their phone-a-friend, so we get lots of phone calls or just questions.*

Feeling supported by those with specialist preterm surveillance knowledge and the resources they could provide (through help with guidelines, networking, shadowing, training, asking questions and/or referring patients), meant smaller units were able to develop confidence in caring for these women at risk of preterm birth, and women would receive less variations of care.

Outcome – Less variations in care for women within the region

Refined theory Programme Theory 6 after data collection: IF sites work together with their local network (C), THEN there are likely to be less variations in care for women within the region (O), BECAUSE they will then have the resources to redefine the pathway appropriately at their local unit (M)

If sites work with their local network, then there will be less variation of care for women within that region. An interview with a consultant obstetrician illustrated this full Context-Mechanism-Outcome triad is below:

Consultant obstetrician HSI005: *“Yes, and I think also, in the long run they [UCLH] were very clear that the world we are going to be working in is a networking world, it’s not that [our unit] can stay a satellite zone and do its own thing (C) and actually show numbers that we are doing amazingly because we’ve not had an SI [serious incident] on one of our patients because we are so good. It’s not that world anymore, it’s about co-ordinating with each other. And hence I found that co-ordination quite helpfully done with UCLH, because I think the whole team was quite open to helping and sharing things (M), which was great…* *And I think my aim always was to reduce the variation (O) between how we do things compared to others…whereas that was one of my main things when I came in, that I can’t run a clinic which runs so very different from the unit next door.*

She continued: *“…at the end of the day, it’s the patient. And in today’s world, a patient of [our unit] may not stay at [our unit], so she might end up going to …UCLH, and whatever we can do in our own capability to reduce the variation, and it’s the stress factor, so I think that was my main aim.”*

In the same interview the consultant obstetrician gave another full Context-Mechanism-Outcome triad example. In this example, by working with her local unit, she had the resources of their audit which she could model her own on, to demonstrate that their referral criteria could change to match that of Saving Babies Lives Care Bundle Version 2 (therefore reducing variations of care).

Consultant obstetrician HSI005: *“I think with the audit we have done, we’ve shown that me…not seeing single LLETZs has worked because all the single LLETZs have pretty much delivered…at term (O) – so I think that was one of the main reasons why I was quite drive to start that stage of the audit…I think that culture change took a bit of standing up to do [laughs], because you know when you’re the new person who comes in and things were done a certain way for more than a year – because that clinic started in January 2020, I took over in April 2021, so almost 16 months of it – and you’re like “No, well this is the feeling maybe…” So it was just to prove that however having said so, I saw the audit from UCLH (M) and that actually helped me quite a lot (C).”*

An interview with another consultant obstetrician illustrated the oppositional theory of Programme Theory 6 with a full Context-Mechanism-Outcome triad below:

*Consultant obstetrician LSI004: “…my big concern is… I think we are fine here, but it’s these referrals, and I know there are people slipping through the net, so I think it’s really important for us to try and sort out our LMS and our regions (C). I think people are not… They don’t have named people or if they do they are not given time to do it and they don’t understand… So I want to do a big teaching thing for our LMS preterm leads (M), because although they are saying they are preterm birth leads, they are absolutely clueless of the pathology of preterm birth and I think that’s where our big thing is really. So, as I say, I’m quite happy with what we’ve got in these and we are really lucky, but I know that women out there are not getting the care that they should be getting (O).”*

**Programme Theory 7 – Adequate finances and resources**

Original IPT: IF management ensure that the preterm pathway is adequately supported with adequate finances and resources (C), THEN the pathway is more likely to be implemented successfully (O), BECAUSE staff will not have to worry about these periphery issues (such as funding, managerial support, time etc) and therefore can focus on delivering the pathway, feeling motivated and encouraged by their managers (M)

Refined Programme Theory 7 after data collection: IF management ensure that the preterm pathway is supported with adequate finances and resources (C), THEN the pathway is more likely to be implemented successfully (O), BECAUSE staff will feel supported in delivering the clinical care that is required (M)

Programme Theory 7 only required slight tweaking from the original IPT. This included changing the mechanism from ‘worry’ to ‘support in delivering the clinical care’ required. No participants in interviews used the word ‘worry’, although they did use the word ‘support’. While these initially seem similar, they do differ in that worrying can take up excess energy. Meanwhile support doesn’t do the opposite and provide additional energy, it simple doesn’t deplete what one has.

Context – Adequate funding and resources

Staff at all the three sites felt the pressure of lack of funding and resources in the current NHS.

Consultant obstetrician HSI002: *“It is difficult, the NHS, for all of us at the moment. It is a complete nightmare.”*

***

Midwives talking amongst themselves in observation 082: *Saying how they would want to ‘I’d work anywhere that’s not clinical’, highlighting the stress of clinical practice being so short staffed that they would rather work outside of it.*

***

Consultant obstetrician HSI002: *“We had our Ockenden review, like I said, recently, and we didn’t know when they were coming. They told us 4 days before they were coming, and half of us were on leave that week. I wrote a whole spiel about what we needed…I gave it to a colleague of mine who I share an office with. I said, ‘Would you mind going and talking about this to them?’ He very kindly did. But no one asked me, managers or anyone, saying, ‘What do you think you need so that we can let them know?’ I found that really interesting. I am sure it is the same in most units across the country. I don’t know. So, if we want that national conversation that we need and we need investment in our maternity services, it has to start on the shop floor.”*

***

Superintendent sonographer YSI002*:* “*I suppose I’m mindful of the waiting lists that we’ve got elsewhere. So any spare capacity we do try to use... So it’s that constant juggling. …I say that it’s just, you just have to have that scrutiny. I do scrutinise our workload a lot. And I will do it the week before to make sure things are mapped in for the following week.”*

Some cases highlighted specific areas (equipment for example) that was missing or broken. While they could undertake their job in the preterm surveillance clinic, it meant it was a more difficult place to work. Jobs took longer than they would have otherwise done, and/or staff stretched themselves to ensure that the women did not miss out.

Consultant obstetrician LSO003 in observation 012: *Explaining to service user LWO010 that the printer is broken so will have to write in her hospital notes by hand, rather than be able to print out a preterm surveillance report.*

***

Consultant obstetrician LSI004: *“I think we need a second machine. I think it would work better if me and [the other specialist preterm consultant] ran separate rooms because you wouldn’t feel as stre… we are so overbooked but we’ve only got one machine, so you are sort of running from room”*

Some felt that this was compounded by the priorities of managers in their organisation.

Consultant obstetrician HSI002: *“I think in our board, it is very much, ‘What is the latest thing handing down from Ockenden or NHS Resolution or NHS England that we need to tick? So, let’s do that, because we have just been told we have to do it,’ rather than proactively saying… Our hospital is very unique. It is a very small DGH. … So, we punch above our weight. We are not a teaching hospital. All the other level 3 units are at teaching hospitals. It is a very unusual set of circumstances. “*

Mechanism – Feel supported

In some sites, management felt there was little leeway in controlling resources to enable staff to feel more supported.

LMNS Maternity Transformation Midwife/Maternity Matron YSI001*: “I think it depends what [the member of staff said] they wanted to change. If it was just tweaking the pathway to, I don’t know… It would depend. I mean we would definitely listen, but if it was that they wanted a set [preterm surveillance] clinic or they wanted more midwifery time, it would depend what it was, because obviously we’ve only got the resources that we’ve got.”*

However, if management mitigated the effect of the lack of finances and resources, it meant staff felt supported in delivering the clinical care that was required.

Consultant obstetrician HSI005: *“And instead of the 0.2%, the Head of Midwifery said “No, I think it’s better if we advertise [for a specialist preterm midwife] at 0.4 because if you look at it, the clinic will expand and then it will be much easier and quicker to get someone,” so it’s all gone straight through so it has been very, very well supported.”*

*

Consultant obstetrician LSI004: *“When we wanted the [specialist preterm] midwives, we went to our head of midwifery and at first we were like mmm. They were very supportive and, as I say, they are really sweet in that they’ll say, “Oh this is such good preterm birth care,” and they know that we are well respected around the region, that we get referrals in, but it was just literally we didn’t have enough midwives on the shop floor for the general stuff so how could they free up specialist midwives to do other things? But as Covid passed on, they managed to get some funding from the LMS [Local Maternity System], I think. I think from the Preterm Birth Fund. Because [the other specialist preterm consultant] and I were already in post, they didn’t have to give us that, so they could give that to… whereas other units that didn’t have preterm birth clinics, I suppose the first thing that they need to do is get someone to have time to do that. So because we were already better off, we were already sort of an established clinic, it didn’t matter so much.”*

***

Preterm midwife LSI001: *“It’s very much a luxury. Because we already had the pre-term clinic up and running successfully, the money that’s been earmarked from Saving Babies’ Lives, we've been very fortunate that it can be used for our [specialist preterm midwives] hours.”*

Sometimes, management did control resources to support staff in one aspect, but were then aware that this then created issues elsewhere.

Superintendent sonographer YSI002: *“…the only way to sort of bring in new practice is make things work is to rob Peter to pay Paul…I’ve literally moved one problem to another place, but it has meant that we haven’t had to go through business cases, that we haven’t had to delay anything, it’s happened very quickly.”*

Outcome – Successful implementation

Refined Programme Theory 7 after data collection: IF management ensure that the preterm pathway is supported with adequate finances and resources (C), THEN the pathway is more likely to be implemented successfully (O), BECAUSE staff will feel supported in delivering the clinical care that is required (M)

Three interviews with consultant obstetricians and two different sites illustrated full Context-Mechanism-Outcome triads below:

Consultant obstetrician LSO003 in observation 038: *Explained that their preterm surveillance clinic was set up how they wanted it to be, and they were not told how to set it up (M). This meant it was flexible to meet their demands and needs (O). When asked how they achieved this, he joked that they ‘bullied managers into this’ arrangement! (C)*

*

Consultant obstetrician HSI002: *“She was given the Friday afternoon slot (C), which I immediately said, because I saw we have a new consultant, a new thing, and we are doing it on a Friday afternoon. It is the most ridiculous thing. And also, what will happen is, she will pick up someone who needs a suture, it’s a Friday afternoon. So, she will end up having to wait the weekend, or being done on the weekend by some random person who has no interest in preterm labour (O). It took ages to finally, now, I think she is doing it on a Wednesday afternoon.”*

She continued: *“Yes, it’s insane. Every time someone… We are now appointing another consultant and then they are operating this on a Friday afternoon. I don’t know why we do this. Every time a new consultant comes, they have to be operating or doing something on a Friday afternoon, when no one is here to support them (M). …I always thought, at least if your preterm clinic was on the same day as somebody else’s preterm clinic in London, then you could just pick up the phone and say, ‘I have got this woman, I don’t know what to do. What would you do?’ That would make sense.”*

***

Consultant obstetrician HSI005: “*No, I’d say anything to introduce has been a challenge. For me, obviously it was a challenge because it was a learning curve for me. I’d never done a business case, I’ve learned doing a business case and for the first time ever, I found out how difficult it was to get money off the purse strings. [Laughter] So there were multiple [laughs] versions of the business case, and I found it very challenging because our levels of management were changing quite rapidly over the first six months of me being a Consultant. And that was one of the key delays because every new person who was coming was telling me a different thing to do. But the business case changed into an IPT, and then that went round the Head of Midwifery and a few of the managers, but also the key people involved in implementation of this all changed. The Head of Midwifery who was dealing with it when I started left; the new Head of Midwifery took over so there was some confusion how much work had been done, how much hadn’t been….Plus the Finance …also changed, so I think that was the main challenge. So I have to say I found it fairly challenging, but I was very determined for it to happen, so yes.”*

She continued: *“So I wish there was a set pathway of something that “Okay, once it’s been approved by this Finance Manager and this person, the next person you go to is the POCT Team, or the next person you go to is the Procurement Team,” so I think for me that didn’t happen, it was all happening collaterally and all happening a bit in “Go to this person, go to that person,” but I didn’t know who the next port of call was and I think that would be a nice thing to have.”*

She continued: *“No, I have to admit I’ve felt very supported (M) because every time I went to them with a problem relating to the implementation, it was never that “Okay, we don’t know what to do, and go away,” it was always like “Okay, leave it with us, and I’ll find the next person who can deal with it.”(C) That sometimes did lead to the confusion but I have to say overall they were very supportive.”*

*She continued: “…but it just happened to be luckily dealt with, otherwise we wouldn’t have been able to launch [using fetal fibronectin after getting the business case approved] (O) on 2nd of September either.”*

Meanwhile, some consultants felt very clear about their remit of providing clinical care, and purposefully did not involve themselves in aspects of funding and resources which would affect them providing the preterm pathway care they desired to women.

Consultant obstetrician LSO003 in observation 012: *Explained how he doesn’t encourage women to book at their hospital just so that they get the tariff payment. He said this was partly due to their geography, and they have women who come to this clinic who can live a long way away. It was also because finances are not their remit or concern, they are there to provide clinical care.*

***

Consultant obstetrician LSI004: *“We have the odd… again, when a new CD [clinical director] came on, the first thing, they were like, “All these stitches that you are doing for out of area are we getting the funding for?” and questions like that, but again we were like, “Oh well we’ll look into it,” and over time they just forget about it and they are like, “Yes, whatever”. Because ultimately it all comes from the same pot. The NHS only has so much money and these women need that care. If the trust really, really wanted to claim that back and make sure they were doing it then they need to sort out the processes, don’t they?”.*

There are similarities between Programme Theory 7 above, and Programme Theory 3 in Chapter 6. While Programme Theory 4 focused on how resources could affect suitable risk assessment and referral to a preterm birth surveillance clinic, Programme Theory 7 highlights how appropriate resources ensures the preterm surveillance clinic is implemented effectively for those women who have been referred.

# Explanatory area 3: Women centred care

*Table S2.3 IPT and questions asked of the data for ‘women centred care'*

| **Explanatory area** | **Programme Theory** | **Nodes incorporated** | **Original IPT** | **Subsidiary questions** | **Refined Programme Theory** |
| --- | --- | --- | --- | --- | --- |
| Women centred care | Programme Theory 8: Continuity of care | - Continuity of care - Fragmented care | IF patients have continuity of care, THEN they will not have to repeat their medical history at each appointment. This means that they feel listened to and that they are receiving personalised care, which encourages trust and patient satisfaction with clinicians. (Continuity) | 1. What makes you feel you are receiving fragmented care/ continuity of care? 2. How do you think this impacts you? | Refined Programme Theory 8 after data collection: IF women have continuity of care with the preterm specialist team (C), THEN they will feel reassured in the preterm care they are receiving (O), BECAUSE they have developed a relationship with their preterm clinicians (M) |
|  | Programme Theory 9: Additional support | - Trust and confidence with clinician - Specialist knowledge (1.1) | IF a woman is labelled as high risk of preterm birth, THEN she may feel relived as the label offers her a sense of reassurance with more frequent clinical assessment, which therefore reduces her anxiety. (Women’s views, needs and perspectives) | 1. Could you explain how being given the label of being high-risk is important? Could you explain what you feel this represents (their history)? 2. What ideally does this clinical assessment provide? I was thinking it may provide reassurance/feelings of listened to/not neglected? | Refined Programme Theory 9 after data collection: IF clinicians ensure they are aware and attentive of a woman’s history (C), THEN women will feel more confident in their pregnancy (O), because they will be conscious that the risks to their pregnancy are not being neglected (M) |
|  | Programme Theory 10: The woman behind the risk | - Remain ‘normality’ | IF clinicians acknowledge that women are likely to have different/mixed feelings about being high risk of preterm birth, THEN they are more likely to offer individualised care, which improves their maternity experience. (Women’s views, needs and perspectives) | 1. Could you describe how women are not consumed by their risk status? | Refined Programme Theory 10 after data collection: IF clinicians ensure ordinary aspects of a woman’s pregnancy are not overshadowed (C), THEN women are more confident in their pregnancy (O), because they feel as though they are not just defined by their risk status (M) |
|  | Programme Theory 11: Time and freedom | - Individualised care (3.2) - Time - Holistic care (3.4) - Time and freedom | IF women are given the space to be offered individualised and holistic care (C), THEN they will feel reassured (O), BECAUSE their care will feel more personalised and inclusive, and they are therefore more likely to have trust in the clinician looking after them (M)  IF clinicians allow women remain in the clinic for emotional and mental support after they have been clinically discharged (C), THEN the service will be affected for other women who are coming through the clinic (O), BECAUSE resources of the clinic will be stretched too thinly (M) | 1. How do you offer individualised and holistic care? | Refined Programme Theory 11 after data collection IF clinicians have the time and freedom in preterm clinic appointments (C), THEN women have a more positive experience (O), because clinicians are able to offer individualised care (M) |

## **Programme Theory 8 – Continuity of care**

Original if-then-because statement: IF patients have continuity of care, THEN they will not have to repeat their medical history at each appointment. This means that they feel listened to and that they are receiving personalised care, which encourages trust and patient satisfaction with clinicians. (Continuity)

Refined Programme Theory 8 after data collection: IF women have continuity of care with the preterm specialist team (C), THEN they will feel reassured in the preterm care they are receiving (O), BECAUSE they have developed a relationship with their preterm clinicians (M)

While an if-then-because statement regarding continuity of care was initially created, it was not decided to bring it forward for initial testing during data collection. However, an early interview with a woman re-highlighted the importance of continuity.

Service user LWI001: *“…. she has been there consistently…so that has been reassuring that it is the same person every week. And we have built up a bit of a relationship.”*

This led to the subsidiary questions in Table 1 regarding continuity being asked in subsequent interviews and data collection. Aspects of the initial if-then-because statement were correct but required expanding. For example, rather than the mechanism of continuity being because clinicians know a women’s medical history, it seemed the important aspect was the whole relationship between woman and clinician. Being aware of their prior medical history was one part of that relationship.

Context – Continuity of care

If there is a small group of multidisciplinary specialists in preterm birth at a unit, it means that women attending the preterm birth clinic will have continuity of care at these appointments, and therefore the positive benefits that continuity brings.

Some felt that for women who were at risk of preterm birth, it was the antenatal continuity to get them through those tricky gestations where they may be at risk of delivering early, was more important than continuing and also offering intrapartum continuity.

Consultant obstetrician LSI004: *“There’s obviously some evidence of continuity of care can reduce preterm birth and I particularly believe in that antenatal setting. I’m not too bothered about the intrapartum continuity, it’s about the antenatal continuity. There’s a massive cohort of women, deprivation is linked along with social stresses, so all that’s sort of more psychological, emotional support is a massive player on cause of prematurity and I think having that same team and continuity helps with that.”*

However not all women felt they experienced continuity of care with their specialist preterm appointments.

Service user HWI001: *“I do not think I have any relationship with her [the specialist preterm obstetrician], to be honest. Yeah. We saw each other for fifteen minutes in the last two months or something, so I do not think there is a relationship there. Yeah. That is it [laughter]”*

***

Service user YWI002: *“I think I’ve got different people, different consultants. I think I’ve got one which is supposed to be mine, but there’s so many consultants and doctors through these weeks.”*

Alternatively, some felt that even if they had continuity of care for their specialist preterm appointments, care felt disjointed between the other departments and clinicians seen during their antenatal period.

Service user HWI002: *“I just feel like I talk to her [my midwife] about different things. Like she talks to me about the baby’s movements and informing me about breastfeeding and different topics, whereas with the consultant, I talk about my specialist care.”*

She continued: *“Yes, and I think because of the patient I guess I am, you can appreciate each area, like I really appreciate my midwife, and my consultant, and my preterm specialist and what they’re doing. But it just does feel so disjointed…”*

***

Service user HWI004: *“I felt that they were quite separate in the sense that my midwife wouldn’t, as far as I’m aware, she wouldn’t have had the information that came out of my preterm appointments unless I told her them, unless I told her what had happened.”*

***

Service user HWI005: *“Because I’m high risk, yes, it is confusing, to be honest. I had the preterm birth appointments but then I also had and I am having checks with a doctor. But I’m confused because I know that there’s two sections, isn’t there, when you go to have a scan. I don’t know what the difference is. One is just a regular scan and then the other bit is, I don’t know, is it consultants or something and also the preterm birth clinic, is that right?”*

***

Service user LWI002: *“…the care that I’d received in all of the separate departments has been really good. But I do think I would have felt better informed if the communication between the different departments was better.”*

While women preferred the idea of continuity of care, the current constraints within the NHS were given for reasons as to why women did not always anticipate or expect it.

Service user HWI003: *“I think there’s always a nice idea generally with healthcare, like a very old-fashioned idea, that it would be nice to have your one doctor who knows you and knows everything, and doesn’t need to be filled in. But I think realistically we know that that’s just not the way things work [laughs] in the NHS these days, so I wouldn’t really expect it. But yes, I think as much consistency as possible would be really good…”*

***

Service user HWI001: *“I have been reading about continuous care. It is the same with the midwives because it is not going to be the same midwife who is going to be in my labour and it is… it can be the same doctor that has followed me or not. It does not really… so it is what it is. It is free healthcare, but it would have been nice if there was a continuous follow-up.”*

Mechanism – Developed a relationship

For certain women, continuity of care meant that they developed a relationship with the clinicians caring for them.

Service user LWI001: *“…. she has been there consistently…so that has been reassuring that it is the same person every week. And we have built up a bit of a relationship.”*

***

Service user HWI004: *“Yes, it was helpful because I feel like she remembered… I mean, she remembered me and my partner each time and we were able to build up a bit of a… Even though it’s a very short appointment, like I think you can build up a bit of a rapport and a bit of a relationship with that person. So I do think it was helpful to see the same person, yes.”*

This continuity brought numerous benefits to clinicians and women. These included women not needing to unnecessarily repeat their medical history, consistency (including clinicians utilising uniform language and devising consistent management plans), and an environment where openness was valued. These are outlined below in further detail.

One benefit included women not needing to re-explain their medical history to different clinicians at each appointment.

Service user LWI003: *“I think it helps that I don’t have to explain things every time. For one thing, it helps to keep appointments shorter because it’s giving updates and adding information rather than having to go through my whole back story. I think that’s the main thing really, and knowing them and knowing what they’re like, and having a sort of relationship if that makes sense. I don’t know if relationship is quite the right word, but something like that.”*

*

Service user HWI005*: “I feel like she knows everything and she’s just well informed about every aspect of my pregnancy.”*

***

Obstetric registrar LSI003: *“Yes, because we remember the patients, we remember their story. Obviously we read the notes every time but you’re not just coming at it from trying to make a whole new plan with a new patient every time. And from a job satisfaction point of view, I much prefer it because you get that immediate feedback from your patient, you get that follow through. In general antenatal clinic, you see women, you make a plan, you may or may not see them ever again or speak to them ever again in the pregnancy.”*

This contrasted to the times where women did not have continuity with their clinicians, and felt they did have to re-explain their medical history.

Service user LWI001: *“And when I sat down [at her local unit, instead of the tertiary specialist preterm clinic], she [the doctor] went, ‘How come you are here today?’ I was like, ‘How come I am here today?’ She was like, ‘Let me just have a quick peek on the notes.’ She was like, ‘Oh yeah, I can see why you are here today’ and it was just that one sentence that stuck with me saying, ‘How come you are here today?’”*

*

Service user LWI004: “*Because you don’t always like to mention it at every appointment you have, there’s always something that you want to bring up like if it’s something that you hope maybe the person you’re seeing has read your notes or taken the time just to look over. Like for instance the other day I went just for a 16 week scan, it was the last one I had actually with my Community Midwife and she had a student with her. And she asked the student to just put the – not the scanner but just so you can hear the heartbeat – machine, and she was busy behind the curtain was the main Midwife. And this poor student, she spent a long, long time trying to find a heartbeat [laughter]. You know you need to hear the heartbeat straight away and it’s the most important thing in the world just to hear it, and it just felt like an eternity. And I could see her sort of panicking, and again it was coming up to the time, it was 16, 17 weeks when we had our loss, and I thought if the Midwife had just thought about it or just helped her a little bit rather than just leaving us both there panicking a little bit, and obviously it was fine and she found it eventually, but I think it’s things like that that trigger things rather than day to day stuff. I guess it’s being in that environment and not hearing a heartbeat straight away, where I think when you do have someone specialist looking after you, like the first thing they’ll do is check that you’re okay before they even start talking to you. You don’t want to have a chit-chat before you hear the heartbeat, you want to hear the heartbeat and then have a chit-chat and I think they understand that, but it’s so important to put you at ease straight away. And that’s certainly what I got from [the specialist preterm midwife] …but just, yes, just mental health, just [laughs] I feel like she [the specialist preterm midwife] just understood, she knows what women are going through, she knows what they need to hear, and it’s always nice to be asked how you are. And the fact that she did know it was coming up to this time, it really meant a lot, so I think it’s really important.”*

Women also appreciated the consistency that the same person caring for them brought.

Service user HWI002*: “Well I think because I’m seeing a midwife, I’m seeing a consultant, you go to so many people, so it’s just quite comforting to know that you’re seeing the same person every time, and they can relate to your progress, and I guess your specific profile. I think as well, because, just as a person that has seen a lot of doctors in the past for various different things, I guess consistency just creates comfort.”*

This consistency included the uniform language and management plans that occurred with continuity of clinician.

Service user HWI003*: “I think it’s nice to see a friendly face. It’s also good that – how do I put this – she will phrase things in the same way in each time. She’s not going to suddenly use a different phrase or different terminology and throw us off course. I think it’s good to know that she will… Even just little things, like in the past obviously I had one person tell me I had a subseptate uterus, and I had somebody else say it was bicornuate, and that kind of inconsistency I think can be quite confusing, so it’s good to see the same person, because you know that they will use the same terminology probably, they have the same outlook, the same manner, and it just keeps things a little bit more consistent, so you’re not going to have to go, “Hang on, sorry, does that mean the same thing as what the last person told us, or…?” So yes, I think that’s good. And also just that sense that there are consistent staff members in the hospital. I think sometimes you worry that they’re so understaffed that maybe there’s just random people having to pick up when other people are off sick, and not really knowing necessarily knowing what’s going on with each person, so yes, I think it helps you feel like you’re in safer hands.”*

***

Obstetric registrar LSI003: *“But I think, like I say, in general having that bit more knowledge with preterm birth care, patients are often told, we get told by so many patients, ‘we’ve been told different things by different doctors and different midwives, and different things by different nurses’. And having a clear, consistent message to the patient I think is really helpful for them. Rather than confusing things.”*

***

Service user YWI002: *“They’ve [having different clinicians caring for you] got slightly different points of view sometimes.”*

Women described feeling more comfortable with clinicians who they had seen before and had some continuity with and were therefore more open with them.

Service user HWI005: *“Yes, definitely, I think it definitely affects that. Definitely helps just to feel comfortable and if I have any questions I can email her. Yes. I can tell her anything really, any aspect of my care, whether it’s something to do with the physical pregnancy or mental health and I just feel like comfortable.”*

***

Service user HWI002: *“Yes, I think you’re more comfortable, like when they ask you, “Oh, are you okay?” it’s like everyone asks you, “Oh, are you okay?” and it’s like, “Yes, I’m fine.” But if you’re seeing the same person, it’s like, “Oh well, this kind of came up, should I be concerned?” I just feel a bit more comfortable talking about things, or they know that you’re progressing and they’re sort of happy with how you’re going. So I just feel little bit more comfortable asking them questions or telling them things, or if they have to do something that’s a bit more uncomfortable, because they can’t get the right image or whatever. You just feel a little bit more at ease if it’s the same person, because you know they know you.”*

***

Service user LWI001: *“Yeah, I feel like our relationship does not change at the next appointment, whereas if I had got in touch and said this, I am not embarrassed at the next appointment because I can just tell that nothing has changed. I do not feel bad for getting in touch. He remembers at the next appointment whatever I have been in touch for the previous week or if we have discussed anything, he remembers next time and I do not think the relationship has changed.”*

Likewise, clinicians felt they could be more open with women.

Consultant obstetrician LSI004*: “No, I think it is easier. I mean, obviously if it’s bad news it’s awful because you almost feel like you are telling it to a family member or a friend, don’t you. But I think because you’ve already got that rapport, they trust you and they tell you more than what they would tell someone else. So yes, like the domestic violence situation, I would be probably more confident in saying, “Is everything okay? We’ve not seen such and such for a while,” and they do openly say that… It’s hilarious. We will get young girls who have got a boyfriend, they are like, “Oh we split up,” and then the following week they are there and we’re like, “Ooh what’s happened here?” [Laughs] But you can sort of have that laugh with them or you probably can delve deeper because you know their family dynamics. They come with their mum, you get to know their mum. Yes, definitely, you almost become part of their family, or you know what their family dynamics are so it’s much easier.”*

***

Consultant obstetrician LSO002 to service user LWO037 in observation 035: *‘will you come to your sugar test next week, cos you’ve missed quite a few haven’t you?’, and the woman replying ‘I know I know, I will’. The conversation did not come across as authoritative but direct, and the relationship they have does not make it awkward.*

***

Consultant obstetrician LSI004: *“And likewise, if they are not seeing the patient regularly… You know when a woman is worried if you have seen her numerous times, don’t you, whereas if you’d never met her before you don’t know if this is normal for her or not.”*

Outcome – Reassured in their care

Refined Programme Theory 8 after data collection: IF women have continuity of care with the preterm specialist team, THEN they will feel reassured in the preterm care they are receiving (O), BECAUSE they have developed a relationship with their preterm clinicians (M)

This was highlighted in the context-mechanism-outcome triad below.

Consultant obstetrician LSI004: *“But certainly for the high risk ones, women who have had losses, who have had very extreme prematurity, that anxiety is so high and seeing the same people each week, be it me or [the other specialist preterm consultant] I, or both for each visit, you can see the reassurance (O), they feel that they are being listened to, they feel like we know their story, they don’t need to repeat their story and so that reduces their stress but it also means they are probably more… I think they are more likely to voice their concerns because they don’t feel silly, because they know us and we get a really good relationship (M), with them and so these subtle sort of symptoms, change in discharge or this subtle pain that they might lie awake worrying about, they have the confidence to voice that to us because they trust us and because they know us.”*

Seeing familiar clinicians brought reassurance to woman that they were being cared and looked after, which is especially important for this group of high-risk women.

Obstetric registrar LSI003: *“I think the women feel, they’ve often been through a loss or traumatic experiences of losing babies early and to have that regular meeting with the same person and that continuity of care with a preterm midwife, the continuity of care is proven to reduce preterm outcomes, isn’t it? And it gives them that reassurance.”*

***

Service user HWI002: *“I think as well, because it’s like the one scary thing that is in your pregnancy. So if you’re seeing different people each time, you just feel like you’re going through the system, rather than being cared for.”*

*

Service user HWI005: *“I don’t know. It just doesn’t really make sense to me. I don’t know. It almost feels like it’s like a conveyor belt, you don’t really feel like you are being looked after properly. When you get to know someone, you can kind of build a bond with them. Yes, you don’t have to keep explaining and stuff, they know what’s going on.”*

***

Service user LWI001*: “…that has been reassuring that it is the same person every week”*

She continued: *“…he [the preterm specialist consultant] is so reassuring.”*

***

Service user LWI004*: “…I think for me it’s just, yes, it’s the reassurance that everything is okay and it is as it should be, and I think knowing that you can go to [the specialist preterm team]… and just get that reassurance. And maybe because of loss it shouldn’t be this way, but you feel like … you deserve [laughs] those extra checks and that extra bit of support and reassurance.”*

Service user LWI004 is aware she cannot get a guarantee on when she will exactly give birth, or the outcome for her baby, but still places emphasis on the reassurance that she receives in her pregnancy from being cared and looked after by familiar, specialist clinicians.

## **Programme Theory 9 – Additional support**

Original if-then-because statement: IF a woman is labelled as high risk of preterm birth, THEN she may feel relived as the label offers her a sense of reassurance with more frequent clinical assessment, which therefore reduces her anxiety. (Women’s views, needs and perspectives)

Refined Programme Theory 9 after data collection: IF clinicians ensure they are aware and attentive of a woman’s history (C), THEN women will feel more confident in their pregnancy (O), because they will be conscious that the risks to their pregnancy are not being neglected (M)

While an if-then-because statement regarding being labelled as high-risk was initially created, it was not decided to bring it forward for initial testing during data collection. However, an interview highlighted that it was not the label of being high-risk that was seen as important to women for clinicians to be aware of, but more recognising the history that the label represented.

Service user LWI004: *“…you’re just not another pregnant mum, you’ve got a little bit of history and maybe you might need a little bit more [laughs] TLC…”*

This led to the subsidiary questions in Table 1 being asked in subsequent interviews and data collection.

Context – Aware of their history

Women appreciated it when clinicians were aware of their clinical history.

Service user LWI004: *“And when I went to see her [the preterm midwife] last week, she’d clearly read all my notes and she knew it was about 17 weeks and she knew this time of the pregnancy was difficult for me because it was the time that we had our loss. So as soon as we got in there she acknowledged that, and she made me feel just really important – not that you need [laughs] to feel important – but listened to, and just I felt like she had all the time in the world for me.”*

*Later in the conversation she continued: “…they’re all such wonderful people that know about your loss because obviously you’re there usually for a reason because you’ve had a loss before, and just the general care has just been phenomenal.”*

*

Observation 019: *Consultant obstetrician LSO003 acknowledged that service user LWO032 had a poor experience last time, and how that will cross her mind in this pregnancy.*

*

Observation 021: *Partner of service user LWO027 to clinician said ‘thank you, we really didn’t want what happened last year to happen again’.*

Mechanism – Not feeling neglected

While regular clinical appointments with the specialist team in itself may be helpful to women, it was not the regularity of the appointments but the acknowledgment of their clinical history within those appointments that was important as this enabled the mechanism of ensuring women did not feel neglected.

Service user LWI001: *“I was still worried that I could get an infection, but it reassured me. Every time I did a urine sample in [the tertiary hospital], it was in a red pot and they sent it off, whereas… and they might have done that in [the local district general hospital], but I just did not see it, whereas I saw in [the tertiary hospital] them bagging it up ready to be sent to the lab. I just think [the local district general hospital], a lot of time, they did swab testing and said, ‘Oh yeah, it is clear,’ but I think because of everything that happened with [my daughter] and we did not know why she came early, I was still like, ‘Could you still send it off just in case?’ but they were like, ‘There is no protein, there is nothing.’ But I was like, ‘But could you still send it off?’ So, it was a lot more clarity knowing that [the tertiary hospital] just did that as a given.”*

***

Service user LWI004: *“And I think it was just that feeling that you were going to get supported, not just for the stitch but you were going to get looked after a few weeks after that, and right up until whenever you decided to stop really. And it just felt like you had your own sort of [laughs] private, personal doctor, even though I know obviously he sees hundreds and hundreds of patients. I think it was just that feeling that someone was there for you 24 hours a day and it didn’t feel like a battle to ever get hold of him, like if I had a daft question he would never make you feel like it was a daft question even though [laughs] he must repeat himself thousands of times a day with different women. But I think, yes, it was just that feeling of being looked after and knowing that someone cares…”*

***

Service user HWI002: *“Yes, I was quite happy with it, the additional care and how immediately it was offered put me at ease…”*

***

Service user HWI003*: “I think [because of my bicornuate uterus] she [the booking midwife] probably just said, “Oh right, okay, well that’s something that we will keep an eye on.” And she definitely would have been the person I guess who referred me to the* *Preterm Birth Clinic, so I think overall, when I came out of that booking appointment, my impression was definitely that I was being taken really good care of. I felt like, because she also referred me to maternal mental health support as well at that point, because I’ve had anxiety and phobias and stuff in the past. Yes, so I definitely felt like, “Oh good, okay, I’m definitely getting extra care.”*

***

Service user LWI002: *“Because I guess to say, oh you’re going to be referred to a clinic, sounds quite daunting, but if they were saying, for example, you’ll be referred to a clinic for these couple of extra checks, following your treatment [on your cervix], then probably more at ease.”*

Being in a hospital where they felt the clinicians had experience and a reputation of looking after women with a similar medical history also helped.

Service user LWI001: *“Yeah, I felt like if there was a problem, they were more likely to have experienced it in [the tertiary hospital] than in [the local district general hospital]’’.*

***

*Service user LWI004: “I think as well because again, obviously I’d never really come across [the specialist preterm consultant] before, but when I did and they recommend that you join social media groups, [laughs] there are a couple of doctors but it sounded like [the specialist preterm consultant] was up there on [laughs] a pedestal when it comes to helping women with cervical stitches.”*

*Later in the conversation she continued: “So I think the fact that he has got such good reputations…it feels like he goes the extra mile for anybody…”*

***

*Service user LWI003: “And then also, when I got the appointment letter to see which Consultant I’d have, I did do a big of Googling and looked them up and saw their experience and qualifications, and that helped as well.”*

*She continued: “Yes, yes, and any questions I’ve ever had, they’ve always answered very thoroughly as well. I don’t think I’ve ever thrown them with anything I’ve asked.”*

*Later in the conversation she continued: ““I mean to be fair, everyone was lovely at [the local district general hospital] [laughs] as well but I don’t know, we just felt a little bit safer at [the tertiary hospital].”*

Outcome – Confidence in their pregnancy

Refined Programme Theory 9 after data collection: IF clinicians ensure they are aware and attentive of a woman’s history (C), THEN women will feel more confident in their pregnancy (O), because they will be conscious that the risks to their pregnancy are not being neglected (M)

This was highlighted in two Context-Mechanism-Outcome triads below.

Service user LWI001: *“I had my first appointment at [the local district general hospital], one of my first scans, and I literally left it crying. I was like, ‘Why the hell have we done this? Why are we pregnant again? Why are we doing it?’ whereas when I went to [the tertiary hospital], I felt like the way [both specialist preterm consultants], they had seen it one hundred and one times, there was nothing to worry about. It was literally like I was just going for a walk in the park. (C) They were not concerned. Whereas in [the local district general hospital], I felt like… I could just tell the anxiousness. And yeah, they put me at ease. They were like, ‘We could do this or we could do this.’ They gave me loads of different options. (M) So, yeah, I felt so much better at [the tertiary hospital]. (O)”*

***

*Service user LWI004: “And maybe because of loss it shouldn’t be this way, but you feel like you’re allowed to see people because you’ve had a loss before (C) and you know what it’s like and you’d never wish that on anyone else, so you just feel like you deserve [laughs] those extra checks and that extra bit of support and reassurance (M). To be fair, I’d have scans every day [laughs] if I could. It’s just so nice to see and it makes you so happy to see the baby; I think because you have lost you know how precious it is and you’d hope it won’t be the last time that you would see your baby, but every time I go for these scans I really make sure I take it in because you don’t know what’s round the corner, and obviously being really lucky with our second pregnancy that it resulted in our little boy, but I think you just don’t take anything for granted so just to have these appointments and have these scans, yes, it’s just reassurance, it’s a relief and it gets you through the next week or the next two weeks until your next one (O). Yes, it’s so important [laughs].*

An oppositional triad was also identified.

Observation 080: *Service user LWO055 asked the booking midwife LSO005 ‘what might be different for us this pregnancy? Before [at the local district general hospital] our community midwife talked about having regular cervical length scans from 16 weeks’. She also talked about progesterone too, [although] the GP said no. But I don’t know if I can have that?’ (M) Midwife LSO005 replied ‘that is something the… [specialist team] … deal with so they can deal with that once you are referred’ (C), to which service user LWO055 asked ‘how long will that be?’ Service user LWO055’s voice seemed nervous, and you could visibly tell that she was anxious from continually looking at her partner (O). Midwife LSO055 replied ‘about 2 weeks?’.*

## **Programme Theory 10 – The woman behind the risk**

Original if-then-because statement: IF clinicians acknowledge that women are likely to have different/mixed feelings about being high risk of preterm birth, THEN they are more likely to offer individualised care, which improves their maternity experience. (Women’s views, needs and perspectives)

Refined Programme Theory 10 after data collection: IF clinicians ensure ordinary aspects of a woman’s pregnancy are not overshadowed (C), THEN women are more confident in their pregnancy (O), because they feel as though they are not just defined by their risk status (M)

While an if-then-because statement regarding risk status was initially created, it was not decided to bring it forward for initial testing during data collection. However, an early interview (with LSI001, a preterm specialist midwife) highlighted normalising care for high-risk women. This was reiterated in early observations (e.g. observation 009), and led to the subsidiary questions in Table 1 being asked in subsequent interviews and data collection.

Preterm midwife LSI001: “…*but also to normalise things for the women…”*

*

Consultant obstetrician LSO003 to service user LWO015 in observation 009: *‘It is important for you to feel as normal as possible, not in this medical bubble’*

The initial if-then-because statement was not correctly focused. Instead of the attention being on women’s mixed feelings around being labelled high-risk, the refined CMI011 centred on clinicians ensuring they did not overlook the other aspects of a woman’s pregnancy.

Context – Ordinary aspects not overshadowed

If clinicians in the preterm surveillance clinic ensure women are not fully focused on their risk status, then they can begin to normalise their pregnancy.

Preterm midwife LSI001: *“My part, really, that I’d like to expand over a few years is if we could as midwives do more home visits, to normalise things a little bit more. Because I worked in the mental health and twins team before, we used to do the routine antenatal care at home and it helped with that relationship,* *but also to normalise things for the women, because it’s very high risk but they do still need to have those conversations about labour and birthing in a relaxed environment.”*

This was aided by acknowledging that they were experiencing a pregnancy at high risk of preterm birth but ensuring that this did not overshadow the ordinary aspects of their pregnancy.

Preterm midwife LSI002: *“…if you just put them in a normal antenatal class, they would not be able to relate to everyone there. We want to make an antenatal class for high-risk, preterm women.”*

***

Consultant obstetrician LSO003 to service user LWO014 in observation 010*: ‘We’ve had that slight anxiety at the beginning…without denying elements that make you special. Now it is time to be normal!’*

Sometimes women felt that they achieved this sense of normality from seeing their midwife, rather than the specialist team they were under.

Service user YWI001*:* “*I ended up seeing my midwife so infrequently in comparison to how much I was seeing the consultants. The midwife felt like I got a chance to speak about how I was feeling about things and stuff like that more than necessarily the growth of him. So it was quite nice to have that where I could talk about it and give my perspective to the midwife kind of thing.”*

*

Service user HWI005: *“I feel like she [my midwife] knows everything and she’s just well informed about every aspect of my pregnancy.”*

She continued: *“I can tell her [my midwife] anything really, any aspect of my care, whether it’s something to do with the physical pregnancy or mental health and I just feel like comfortable.”*

She continued: *“I don’t know, it just felt like a very quick [my preterm surveillance appointments] … It was very quick and no it didn’t really feel in-depth. Yes, completely different to my midwife appointment.”*

*

Service user HWI002: *“…I think she’s [the midwife] great, but I just feel like I talk to her about different things. Like she talks to me about the baby’s movements and informing me about breastfeeding and different topics, whereas with the consultant, I talk about my specialist care.”*

Mechanism - Not defined by their high-risk status

If clinicians ensure ordinary aspects of their pregnancy were not overshadowed, then this enabled the mechanism of women not solely being defined by their ‘high-risk’ status.

Service user LWI002: *“The preterm birth clinic, I’m under the impression that I’m actually a low risk pregnancy, rather than high risk.”*

***

Consultant obstetrician LSO003 to service user LWO015 in observation 009: *‘It is important for you to feel as normal as possible, not in this medical bubble’*

*

Consultant obstetrician LSO003 to service user LWO007 in observation 014: *‘Your midwife will talk about what’s normal for any mum. Sometimes it’s difficult when you had issues in the start of the pregnancy to then remember to focus on the usual pregnancy things.’*

***

Consultant obstetrician LSO003 to service user LWO003 in observation 016 to 017: *‘…she [the local midwife] will help with things to do with normal pregnancy, preparing for normal pregnancy, things that normal people think about when having a baby.’*

In some cases, this led women to feel they had agency over their care and the difference choices available to them.

Service user HWI003: *“And yes, the main thing really I think was that I was sort of, for the last few years knowing that I had this heart-shaped uterus, I had been kind of assuming that I would probably have to have an elective C-section, if a maybe, so I’d been telling (unclear 0:07:39.4), kind of prepared it in my head, I’d been very much like, “Okay, well, that choice is taken out of my hands, I’ll just have a C-section, fine.” And so it was that appointment, actually no, I think she did mention it at the 8-week scan as well, she said it didn’t look particularly pronounced, and she couldn’t really see a reason that I wouldn’t be able to have a vaginal birth. But yes, the consultant at the Preterm Clinic said the same thing, was very much like, “We don’t see it as any obstacle to having a…” I can’t remember how they phrase it, I don’t think they’d say ‘normal birth’, but you know what I mean. And so I was slightly like, “Oh, okay, so I’ve got to make a choice, have I?” [Laughter] I was sort of looking forward to just having it taken out of my hands, so I didn’t need to weigh up the pros and cons myself, or make any kind of decision. But she made it very clear that their job was to support me whatever kind of birth I chose. But yes, no, I’d say it was a really positive experience, yes.”*

In several instances, the language utilised could do the opposite. In the below example the reasons why the woman wished to give birth in the birth centre, or how this could potentially be replicated in the delivery suite was not discussed. The consultant obstetricians authoritative reply of ‘yes’ in response to the woman’s question provided no leeway. Here the woman seemed defined by her risk status.

Consultant obstetrician HSO001’s conversation with service user HWO010 in observation 053: *Consultant obstetrician asked if she had any questions, to which she asked about whether she would be able to give birth in the birth centre.*

*Consultant obstetrician HSO001 replied ‘well you have a uterine abnormality so there’s a chance of rupture – the first sign would be baby’s heart rate so in the birth centre we don’t continually monitor you’. Service user HWO010 then asked ‘so I’ll have to be continually monitored?’, to which consultant obstetrician HSO001 simply replied ‘yes’.*

The outcome regarding the birth preferences and ultimate delivery of service user HWO010 and her baby are unknown. However, what is important here is the lack of discussion the consultant obstetrician gave around birth plans, risks and benefits of different options, and of the woman’s wishes and choice. The conversation, and ultimately her as a woman, were defined by her risk status.

Outcome – Confidence in their pregnancy

Refined Programme Theory 10 after data collection: IF clinicians ensure ordinary aspects of a woman’s pregnancy are not overshadowed (C), THEN women are more confident in their pregnancy (O), because they feel as though they are not just defined by their risk status (M)

By not being defined by their high-risk status, it meant women were more likely to have a positive experience and feel confident in their pregnancy. This was highlighted in the context-mechanism-outcome triads and dyad below.

*Service user LWI001: “I always come away from my [preterm clinic] appointments feeling like I am just a normal pregnant person (O) even though everything that has happened (C), still at the end of the appointments, it is just like everything is reassuring (M)…. Yeah.”*

***

*Interviewer: “…do you think it would make you feel less confident in the pregnancy?”*

*Service user LWI004: “Oh, I think definitely, I think because yes, maybe even if Mr Simpson didn’t scan you that day, even if it was just ringing and hearing his voice and him reassuring you that actually that’s quite normal at the moment at this stage in pregnancy (O), just someone to say “You’re okay,” (M) I think is really important.*

***

*Service user HWI001:* “*So, because I have the bicornuate uterus…she examines my cervix and… on the second consultation, which was just before twenty weeks or just after… I do not know. I cannot remember. But I asked something about if everything is okay and I do not know how it got there, but it got to the point where we were talking about birth. …And I was like, ‘Okay. But do I need to choose a c-section? Can I have a normal birth?’ and she was like, ‘Oh no, you can have a normal birth, but you should research on emergency c-sections, and we can talk about your birth plan on the next appointment’ (C) and I went home and I started looking for emergency c-sections and…I did ask her for more information and literature on normal delivery and bicornuate uterus and what are the numbers, if there are any numbers or anything, and what is the rate of complications, so she said she was going to research and have that ready for me for the next appointment (M).”*

*She later continued: “I am pretty anxious about… or I was pretty anxious when I found out about the bicornuate uterus, but with all the reactions that I have had around me from the GP, the midwives, even the obstetrician, it is making me much more relaxed because no one is really too concerned about it, or it feels like no one is really concerned about it, so I am like, ‘Okay, if no one is concerned, they should know,’ so why bother? Yeah. (O)*

The confidence this gave women enabled them to explore external activities, such as aquanatal classes, without being discouraged by the requirement of an obstetric note due to their high-risk status*.*

Service user LWO019 in observation 027: *Asked obstetric registrar LSO004 if she could get a note as she would like to start aqua-natal classes, but she is finding that they won’t let her start until she has a letter from the doctor.*

## **Programme Theory 11 – Time and freedom**

Original IPT: IF women are given the space to be offered individualised and holistic care (C), THEN they will feel reassured (O), BECAUSE their care will feel more personalised and inclusive, and they are therefore more likely to have trust in the clinician looking after them (M)

Original IPT: IF clinicians allow women remain in the clinic for emotional and mental support after they have been clinically discharged (C), THEN the service will be affected for other women who are coming through the clinic (O), BECAUSE resources of the clinic will be stretched too thinly (M)

Refined Programme Theory 11 after data collection: IF clinicians have the time and freedom in preterm clinic appointments (C), THEN women have a more positive experience (O), because clinicians are able to offer individualised care (M)

An early interview highlighted that while continuity could lead to women developing a relationship with their clinician and therefore feeling reassured about their care, it did not guarantee reassurance. If women felt that their appointments were rushed, then they would still not feel reassured even if nothing clinically was amiss, and even if they were having continuity of care, and being seen by the same specialist preterm clinicians for each appointment.

This was highlighted in an interview with service user HWI001.

Service user HWI001: *“[laughter] So, I have had two appointments [at the preterm surveillance clinic] so far and the first one was a bit too late. It was a bit delayed. I had to wait quite a long time, which is not usual. It is fine, but then I felt like it was a bit rushed, the whole appointment. And obviously, it is a doctor, so… I do not know. I guess I was expecting a bit more guidance or questions or explanation, but it was just very rushed, I think. In less than ten minutes, I was in and out. Yeah. She was like, ‘Everything is okay? You are okay. Bye. See you in three weeks or something’ [laughter]. I was like, ‘Okay.’ But it was alright in the sense that if everything is alright, I am okay with it. It is NHS. I do not want to demand too much. But obviously, if you are in a preterm birth clinic, maybe some reassurance or explanation of what is going on. Yeah. It would be nice [laughter] I do not know.”*

Programme Theory 8, Programme Theory 9 and Programme Theory 10 focus on the structures required to ensure a consistent overall baseline of care in a preterm birth surveillance pathway. Programme Theory 11 highlights that within these structures, clinicians require time and freedom to provide flexible and individualised care.

Context – Time and freedom

Both Site H and Site L had a specialist preterm birth clinician(s) who provided continuity to the women they saw in the clinic. Site L held a preterm clinic twice a week and was better staffed with a group of multidisciplinary specialist preterm clinicians. This often meant that two clinicians trained in transvaginal cervical length scans would be present in each appointment.

Consultant obstetrician LSI004: *“Some weeks are clinics are totally overbooked but we just deal with it and that’s where we often double up and it’s just one scan, one type and it’s done. Now you could say do the managers say, “What’s the point in having two consultants in a room, that’s a total waste of resource?” and I totally agree, why should a consultant be typing away? But if you are on your own, there is no way you would get through that many people and also I don’t think if I had, say, a midwife to type or a support worker [who was not trained in cervical length scans] to type I wouldn’t be as quick because I’d have to be telling them what to write, whereas again it comes back to that respect that me and [the other preterm consultant] have, if he’s talking, he will trust… he doesn’t check the scan report that I’ve written for him, do you know what I mean?”*

Appointments at Site L felt relaxed, with time for lengthy appointments and freedom for flexibility during the clinic (for example, colleagues making each other cups of tea (observation 013)).

Meanwhile Site H was run by skeleton staff (one consultant obstetrician with the help of a maternity support worker). The fledging clinic ran once a fortnight. During observations of this clinic, it was noted how despite offering women appropriate screening tests and treatment options, the appointments seemed rushed, and staff seemed busy (observation 008). While the allocated appointment length may not have been any shorter than those at Site L, the appointments felt shorter due to their rushed nature. While the consultant seemed just as friendly and approachable as those at Site L during one-to-one conversations before and after clinic began, her body language did not signal this during the frantic rush of the clinic. The tight schedule and heavy workload meant she did not have time or freedom for flexibility during the clinic.

If women felt that that clinicians were rushed during appointments, then this affected the care that they felt they received.

Interviewer: *“Do you feel that the care you received by her [the specialist preterm consultant] was personalised care?”*

Service user HWI001: *“In a sense, I guess, yes. Yeah. Yeah. Very rushed. That is all. I do not know. Because with the midwife, I still have more time with the midwife than with the OB [obstetrician] and I understand that they have a lot to do, but it does feel like an assembly line and they are just turning people [laughter]”*

***

*Service user HWI004: “I wouldn’t say I lost trust, but it makes you just feel like a number in the system and not like a human being. There’s an element of this is my first baby, I’m quite nervous because I’ve had spotting and things before and from a personal point of view. It’s a bit of an empathy thing. I appreciate you’ve got loads of people to see but also this is really important to me and it’s only going to take an extra 30 seconds for it to be the right experience for me.”*

Some clinicians were happy to provide additional time to women.

Consultant obstetrician LSI004: *“And I think we [both specialist preterm consultants] both have the same work ethic. So when I look at teams that don’t function as well as ours, there’s often a worker and then someone that’s a bit slack [laughs]. So [the other preterm consultant] and I will both stay late if we have to. We both care for the patients in the same way and I think we are both there to care for the women as opposed to some people that just rock up to work and want to go home and don’t want to do anything above and beyond. Whereas if a patient needs something we will do it.”*

However, these clinicians often had the flexibility and freedom to provide this sort of care and the additional time it required.

Preterm midwife LSI002: *“Yeah, I think definitely time. Also, we have the freedom… they were very clear in the interview when we got the role. They just said, ‘This is your job role. You have to develop it how you want,’ so we had the complete freedom to build it how we wanted to build it.”*

Mechanism – Individualised care

When the preterm clinic appointments were rushed, details were not explained.

Service user HWI005: “*I don’t know, it just felt like a very quick… It was very quick and no it didn’t really feel in-depth.*”

She continued: *“I think she said the [cervical length] measurement was fine, but I don’t really remember her going into detail. And in general, I felt like it was rushed. It felt really quick.”*

*

Observation 004: *As service user HWO007 was already lying on the bed, naked from the waist down, about to have the probe inserted she was confused about what was occurring and asked ‘with the internal scan, we are trying to see…?’ to which consultant obstetrician HSO001 replied ‘how long the neck of the womb is’.*

*

Observation 005: *Service user HWO004 asked to reconfirm what the consultant obstetrician HSO001 was actually looking at with the internal scan, despite the fact the internal scan had now already finished.*

*

Observation 006: Service user HWO003 went behind the curtain as instructed then asked behind the curtain ‘sorry what am I doing?’ to which consultant obstetrician HSO001 replied ‘removing your knickers’.

*

Observation 006: Consultant obstetrician HSO001 undertook an abdominal scan on service user HWO003. When the scan was finished, service user HWO003 asked ‘there is a heartbeat isn’t there?’, and consultant obstetrician replied, ‘oh yes, sorry didn’t you see it?’

*

Observation 052: Abdominal ultrasound undertaken on service user HWO010. Heartbeat heard for 2 seconds, then consultant obstetrician HSO001 immediately began the transvaginal ultrasound scan.

*

Observation 058: Consultant obstetrician HSO001 explained fetal fibronectin (‘it’s a test to predict preterm birth’) to service user HWO015, and then left the room to find a speculum. Service user HWO015 went behind the curtain as previously instructed and asked me [who was observing] ‘what am I meant to do behind here? Am I meant to remove something?’.

Often the resulting confusion and then explanation from not initially clarifying key details seemed to take more time than if it was clarified originally. Meanwhile in appointments where clinicians had more time and freedom, details were explained from the outset.

Observation 037: *Consultant obstetrician LSO003 explaining the cervical length scans they undertake in the clinic to a new service user. ‘They are perfectly safe in pregnancy. This is the probe [shows her the probe]. Just this section goes in between the labia, then after we can have a scan on the tummy and see the baby if you want?’*

*

Observation 010: *Consultant obstetrician LS0003 introduced himself to service user LWO013 as they had only met the other specialist preterm consultant obstetrician before. Explained how they would ‘check the cervix with an internal scan’ and to ‘…take off your lower garments and go under the sheet [on the couch], while we will go outside [until she is ready]’.*

*

Observation 025: *Obstetric registrar LSO0004 to service user LWO023 ‘excuse me while I turn my back to you and type – do feel free to ask me questions though.’*

*

Observation 019: *Consultant obstetrician LSO003 to service user ‘just give me a minute to tap into the machine [computer] and then we will have a chat’*

In appointments where clinicians had more time and freedom; it was easier to offer individualised care.

Observation 011: *Consultant obstetrician LSO003 told service user LWO012 ‘Don’t worry what appointment time they give you at the front desk, come at 8am and we will see you then as the appointment will be quicker and less wait time’.*

***

Observation 035*: Consultant obstetrician LSO002 told service user LWO036 to ‘make an appointment for 4 weeks’ time, but we are here every Tuesday morning, so if there is any problem do just rock up on a Tuesday morning!’*

***

Observation 014*: Consultant obstetrician LSO003 explaining the cervical length scan to service user LWO006, that it is the same as before, there is sufficient length there and the current treatment plan is good. Then undertakes an abdominal scan so she can see the baby and he pointed out a ‘chubby cheek’ and how the baby is looking ‘absolutely beautiful’.*

*

Observation 026: *Obstetric registrar LSO0004 was aware service user LWO021’s husband was not at the appointment today so said ‘Do you want to hear baby’s heartbeat? You can record it if you want?’*

Outcome – Positive experience

Refined Programme Theory 11 after data collection: IF clinicians have the time and freedom in preterm clinic appointments (C), THEN women have a more positive experience (O), because clinicians are able to offer individualised care (M)

This was highlighted in the context-mechanism-outcome triads and dyad below.

Service user HWI001: *“So, I went there [to the preterm birth clinic] two times and this last time, she offered… she said, ‘I can release you now or you can come another time’. (C) I was like, ‘I will come another time’ [laughter] so yeah, it was good that she gave me the option to choose (O), but she was like, ‘You are fine. You are going to be fine, but we can still see you anyway,’ (M) so I was like, ‘Yeah, I will see you.’”*

***

Obstetric registrar LSI003: *“Well I guess, because the scans are done by us and not through the main scanning department, I guess that gives us the freedom (C) to individualise care. Whereas in the majority of other units, I mean things might have changed since I’ve been in them, but you refer them to the general scanning service and then they come to clinic with their scan. You can’t really justify that service, can you… Because that service provider hasn’t seen the patient and won’t be able to really individualise that care…You probably noticed in the clinic that we’re quite keen to scan women quite frequently around the time that they lost their baby before (M) or had an early… which certainly wouldn’t fit in that kind of strict criteria.”*

***

Service user LWI004: *“I think when we first met him [the specialist preterm consultant], he’s a lovely natured man anyway, but he really took time to explain (C) what might have happened, particularly with the post-mortem; he was extremely sensitive in his language and how much he wanted to tell us and how much we wanted to realistically really know. And I think it was just that feeling that you were going to get supported, not just for the stitch but you were going to get looked after a few weeks after that, and right up until whenever you decided to stop really. And it just felt like you had your own sort of [laughs] private, personal doctor (M), even though I know obviously he sees hundreds and hundreds of patients. …if I had a daft question he would never make you feel like it was a daft question even though [laughs] he must repeat himself thousands of times a day with different women. But I think, yes, it was just that feeling of being looked after and knowing that someone cares (O), and I think just little things like yourself today, like referring to baby by its name and not just ‘he’, ‘she’, ‘the baby’, just little things like that make such a difference.*

An interview with service user illustrated the oppositional theory of Programme Theory 11 with a full Context-Mechanism-Outcome triad below.

*Service user HWI005: “I don’t know, it just felt like a very quick (C)… It was very quick and no it didn’t really feel in-depth.”*

*She continued: “I think she said the [cervical length] measurement was fine, but I don’t really remember her going into detail (M). And in general, I felt like it was rushed (O). It felt really quick.”*

Lack of time and explanation meant women had a more negative experience of the preterm birth surveillance clinic. Sometimes women were not sure what was the point of the preterm clinic appointments.

Service user HWI001*: “Well, to be honest, I am not really sure what happens in the preterm birth clinic apart from I do know that I have to pee into a cup and they see my cervix, so I am not sure there is a lot that needs to be transferred to the midwife on that point unless it is something serious, so I do not know what to tell you if there is a transmission of information or not. I do not really know [laughter] “*

This also led to women not being sure what their plan of care was for the future.

Service user HWI001: *“think it is more… it is down to time again. I do not have the option to ask all the questions that I want. … it would have been nice to have a bit more time to discuss what she thought was the best plan for me so I could consider it and I do not think there was… although I do understand women are supposed to be the ones choosing, it is nice to hear medical opinions too. But yeah, it is more down to that than actually being ashamed of the questions; it is more I just do not have the time to think about them.”*

If women were more alert in these rushed appointments, it was often due to their own prior research and specific questioning.

Service user HWI004: *“And also, I think, to be fair, I did quite a lot of reading on pregnancy in general and hospital appointments and things like that before we even got pregnant and we’ve got a lot of friends who have had children so they’ve got information and we were part of an NCT group and all of these things, so I think we were already quite well informed and we knew the kind of questions that we needed to ask, whereas I’m guessing a lot of people wouldn’t know some of those questions. So yes, I do think that information does… you need to spend that extra couple of minutes before or after an appointment to explain all of those different things that you are looking for, how many appointments will it be, if you’ve got any concerns this is where you can go, these are the statistics. I think that’s really important.”*

Meanwhile if clinicians had more time and freedom, women had a more positive experience, with women described feeling educated about their medical history and pregnancy.

Service user LWI001: *“Well, they made me feel more educated about my own life and my own history and everything that had happened… and then all my first appointments, [the preterm consultant] was like, ‘They have called it this, but in today’s day and age, we would call it this.’ And she did drawings and then [the other specialist preterm consultant] has done little drawings…”*

***

Service user LWI002: *“I think in itself, the preterm birth clinics seem to run really well. The midwife was really helpful, really informative, and then so was the consultant that I saw. He kind of explained to me the measurements that he’d taken, what the measurements were, what that meant…”*

***

*Service user LWI003: “Every one [cervical length scan] I’ve had, I’ve got to have a look at it on screen. I must admit, I don’t know what I’m looking at [laughter] but the Consultant is always very confident, so he shows me and he tells me how long it [the cervix] is, and yes, very detailed.”*

## References

Manzano, A. (2016). The craft of interviewing in realist evaluation. *Evaluation*, *22*(3), 342–360. https://doi.org/10.1177/1356389016638615

Mukumbang, F. C. (2020). The need for retroductive thinking in implementation sciences. *Research Square*, 1–24. https://doi.org/https://doi.org/10.21203/rs.2.24309/v1

Pawson, R., & Tilley, N. (1997). *Realistic Evaluation*. Sage.
